# Beyond years of schooling: Genetic associations across educational milestones in two Norwegian cohorts

**DOI:** 10.1371/journal.pgen.1012310

**Published:** 2026-09-17

**Authors:** Eirik Haugland Kvalvik, Yunpeng Wang, Kristine Beate Walhovd, Torkild Hovde Lyngstad, Ole Røgeberg

**Affiliations:** 1 Center for Lifespan Changes in Brain and Cognition, Department of Psychology, University of Oslo, Oslo, Norway; 2 Department of Sociology and Human Geography, University of Oslo, Oslo, Norway; 3 Ragnar Frisch Centre for Economic Research, Oslo, Norway; Yale University, UNITED STATES OF AMERICA

## Abstract

Although educational attainment is heritable, years-of-education (EduYears) measures are not designed to show how genetic associations vary across discrete educational milestones. In the Norwegian Mother, Father and Child Cohort Study (MoBa; N = 120,527) and the Norwegian Twin Registry (N = 8,910), we combined genome-wide association studies (GWAS), polygenic indices (PGIs), and twin models to characterize genetic associations with high-school completion and bachelor’s, master’s, and doctoral attainment. In transition-specific analyses conditional on prior attainment, observed-scale common-variant heritability (h^2^_SNP_) and PGI discrimination followed an inverse-U pattern, peaking for progression from high-school completion to attainment of at least a bachelor’s degree (HS → BSc + ; h^2^_SNP_ ≈ 0.14; R^2^_Tjur_ ≈ 0.05) and declining at postgraduate transitions. Genetic correlations (r_g_) with large-scale GWAS of EduYears (EA4) and intelligence were high at earlier transitions but lower at later ones (for EA4, r_g_ ≈ 0.92 at HS → BSc+ and ≈ 0.38 at MSc → PhD). In cumulative analyses of attained status in the full cohort, the point-estimate gap between twin- and SNP-based heritability narrowed from ≈ 0.38 at high-school completion to ≈ 0.11 at PhD, while genetic overlap was lower for distant than adjacent milestones (r_g_ ≈ 0.71 for high-school completion versus PhD and ≈ 0.92 for adjacent milestones). Sensitivity analyses using an alternative intelligence GWAS and additional PGIs showed similar transition-specific trajectories. At the doctoral transition, estimates were obtained among individuals who had attained at least a master’s degree, so attenuation there must be interpreted in light of selection and range restriction. Because EduYears converts distinct credentials and progression steps into a single numerical scale, estimates expressed per additional year should not be interpreted as though one year has the same meaning across the educational sequence. Milestone-preserving analyses make that hidden structure visible and sharpen the interpretation of education GWAS and PGIs.

## Author summary

Genetic studies of education usually summarize attainment as years of schooling. Yet people do not simply accumulate years; they progress through credentials such as high school, bachelor’s, master’s, and doctoral degrees. We analyzed these milestones in two Norwegian cohorts containing more than 120,000 genotyped adults and nearly 9,000 twins. Among high-school graduates, analyses based on common DNA variants and genetic scores showed the clearest signal when comparing those who attained at least a bachelor’s degree with those whose highest credential was high school. Signals were weaker at the ends of the sequence, but those comparisons also had different structures: high-school completion is nearly universal in Norway, while doctoral progression is assessed among people who have already passed several earlier milestones. Twin analyses produced their highest heritability estimate for high-school completion, although uncertainty was substantial. These findings show that genetic results depend on the educational contrast being studied and on who is included in the comparison group. A single years-based measure compresses these differences, and one additional year should not be assumed to represent the same educational step throughout the sequence. Treating education as milestones provides a clearer basis for interpreting genetic studies of education.

## Introduction

Educational attainment (EA) is a well-established predictor of adult health [1,2], economic productivity [3] and overall well‑being [4]. It therefore features prominently in social science research as an outcome, a control variable, and an explanatory factor. In recent years it has also become one of the most widely studied social phenotypes in behavioural genetics. Twin and molecular studies show that the customary measure, years of education (often termed “EduYears” in the literature), is substantially heritable [5–7]. Yet a simple year‑count overlooks a basic structural fact: progress depends on clearing discrete milestones, such as finishing high school, earning a bachelor’s degree, completing a master’s, or obtaining a doctorate, each with its own demands. Major demographic surveys, including the American Community Survey and the European Social Survey, reflect this categorical reality by asking, “What is the highest degree or level of school you have completed?” rather than “How many years did you attend school?” [8,9] Even researchers using EduYears implicitly acknowledge this milestone-based structure, as they typically assign “normed years” based on credentials earned rather than actual time spent in the educational system. This structure has long been the explicit focus of sociological models of stratification, which treat schooling as a sequence of decision points [10–13].

While these models have mapped social and institutional influences on progression, they have paid less attention to genetic contributions to the same sequential process, including whether observed genetic signals vary when educational attainment is modeled as a sequence of milestones. Such heterogeneity in observed signals is plausible, as the educational journey likely demands a shifting portfolio of cognitive and non-cognitive skills [14–16], is shaped by complex gene-environment interactions [17], and involves selection processes that progressively narrow observed variance among those who advance, which can attenuate associations within selected groups [18]. This issue has gained urgency as access to higher education expands globally and as technology increases demand for high-skill work [19,20]. The tools used in genetic research, however, have not fully kept pace. Molecular efforts have culminated in progressively larger genome-wide association studies (GWAS) of EduYears, collectively termed EA1 through EA4 [21–24]. The polygenic indices (PGIs) derived from these studies have relied on this continuous metric, without accounting for the discrete, hierarchical nature of educational progression. Conceptually, this means that conventional EduYears GWAS collapse attainment onto a single observed-scale continuum and thereby impose both qualitative uniformity, by treating the same aggregate genetic association signal as applying across stages, and quantitative linear uniformity, by modeling attainment as equal additive increments. This may lead to misconceptions about the uniformity of genetic associations across the educational sequence and about the applicability of years-based genetic measures to specific educational milestones [25]. We address this gap by leveraging the Norwegian Mother, Father and Child Cohort Study (MoBa; 120,527 genotyped adults) and the Norwegian Twin Registry (NTR; 8,910 twins). Across four educational milestones, we characterize genetic patterns using two complementary frameworks.

First, using a transition-specific framework that conditions on prior attainment, we estimate genetic associations with conditional progression at each sequential step. After conducting a GWAS for each milestone transition, we examine external genetic correlations (r_g_) with large-scale meta-analyses of EduYears (EA4 [24]) and intelligence [26], estimate their single nucleotide polymorphism (SNP) heritability (h^2^_SNP_), and test the predictive power of an EduYears-based PGI (EA4-derived). Additional sensitivity and supplementary analyses extend this framework to alternative intelligence measures [27], GWAS-by-subtraction cognitive and non-cognitive traits [28], educational-field axes [29], MiXeR overlap decomposition [30], lead-locus overlap annotation [31], and external-trait PGIs [32].

Second, using a cumulative framework, we model genetic liability associated with attaining each milestone status in the full cohort. This approach enables us to assess the genetic overlap between the milestones themselves and to contrast common-variant heritability (h^2^_SNP_) with the broader biometric estimate of additive genetic variance from twin models (h^2^_twin_), which were only feasible under this framework.

Our findings show that analysing credentials instead of years reveals association patterns that are obscured by a purely time-based approach and refines the interpretation of PGIs now widely used in sociogenomic research. Rather than replacing years-based approaches, we demonstrate the substantial information gained by recognizing education’s milestone-based structure, where success is measured by credentials earned rather than time invested.

## Results

**Fig 1:**
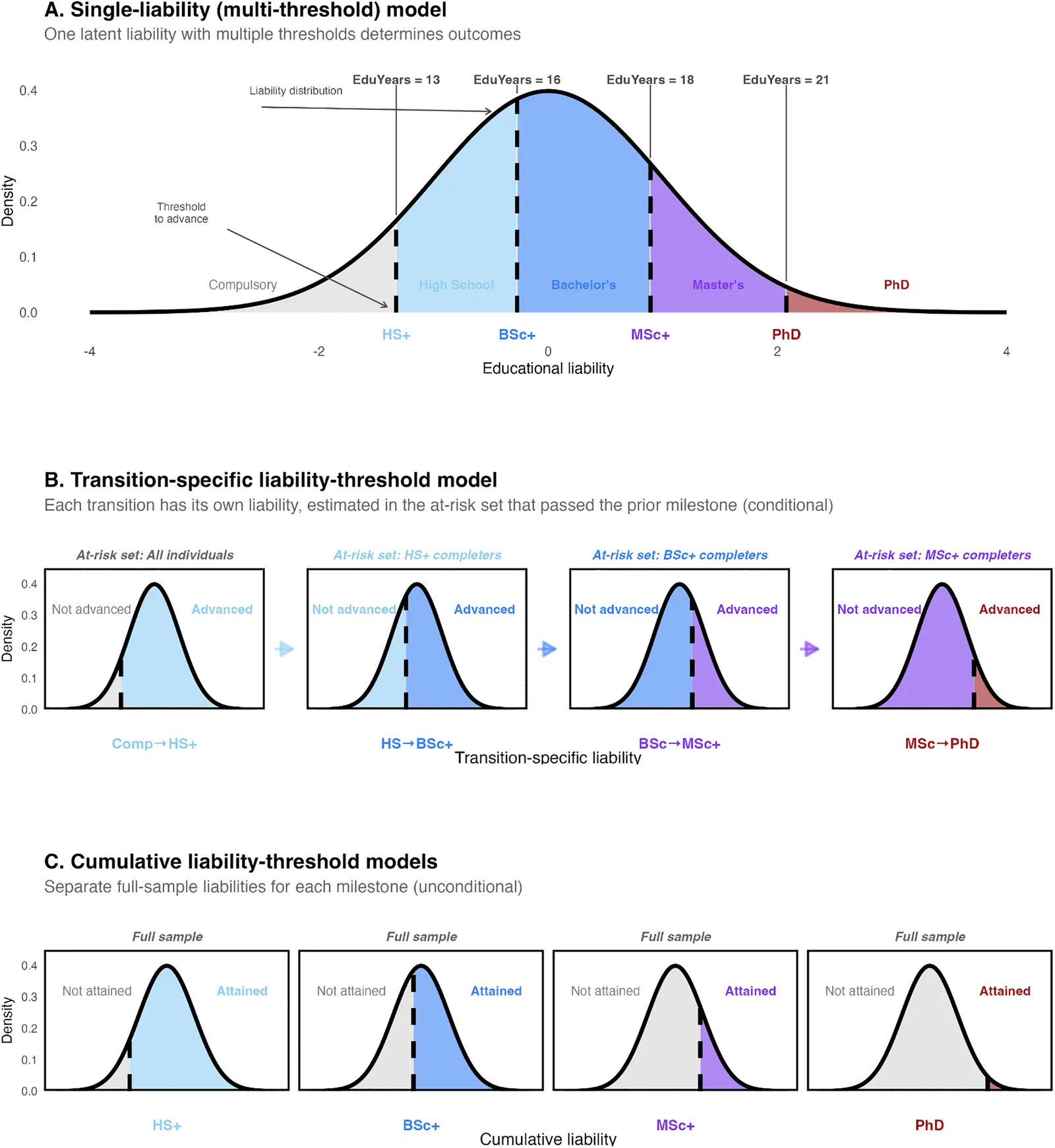
Conceptual models for genetic analyses of educational attainment. **A, Single-liability threshold model.** A single latent liability with multiple thresholds determines educational outcomes. This framework assumes qualitative uniformity, in that the same underlying liability is relevant across the educational sequence, while allowing quantitative non-linearity through unevenly spaced thresholds. It is more structured than conventional EduYears GWAS, which collapse attainment onto a single observed-scale continuum, thereby treating differences between credential-coded EduYears values as additive. The illustrative correspondence between EduYears values and educational credentials shown here is anchored to the Norwegian educational system and should not be assumed to map identically across countries. **B, Transition-specific liability-threshold model.** Each milestone transition is modeled conditionally in the at-risk subset that has already cleared the prior milestone. This framework allows both qualitative heterogeneity, in which the genetic contributors associated with progression may differ across transitions, and quantitative heterogeneity, in which the magnitude of genetic associations may vary across transitions. At the same time, because later transitions are estimated among increasingly selected at-risk groups, observed differences across transitions must be interpreted together with selection and range-restriction effects. **C, Cumulative liability-threshold models.** Each milestone is modeled unconditionally in the full cohort as attainment of that milestone or higher versus all lower levels. This framework captures genetic associations with cumulative attained status rather than conditional progression. Except for the first milestone, the shaded “Advanced” area in each transition-specific panel is larger than the corresponding “Attained” area in the cumulative panel because the transition-specific proportion is calculated within the relevant at-risk subset rather than the full cohort. Accordingly, in MoBa, transition-specific case proportions are higher than cumulative case proportions for BSc+ (66.9% vs. 60.7%), MSc+ (30.7% vs. 18.6%), and PhD (10.1% vs. 1.9%; Table 1).

To address the limitations of treating educational attainment (EA) as a continuous measure, we reconceptualized it as a sequence of discrete milestones. Fig 1 contrasts a conventional single-liability view of attainment with the two complementary milestone-based frameworks used throughout the study. The transition-specific framework estimates genetic associations with progression to the next milestone conditional on having reached the previous one, whereas the cumulative framework estimates genetic associations with attaining a given milestone in the full cohort. This distinction allows us to distinguish genetic associations with conditional educational progression from genetic associations with attained status. We denote “at least high school” as HS + , “at least bachelor’s” as BSc + , “at least master’s” as MSc + , and PhD as doctoral completion; Comp indicates compulsory education or below. We first present the transition-specific results.

### Transition-specific genome-wide association analyses of educational milestones

Following our transition-specific framework (Fig 1B), we conducted genome-wide association analyses in the MoBa genotyped adults (N = 120,527). Specifically, we modeled four sequential transitions: Comp→HS + , HS → BSc + , BSc → MSc + , and MSc → PhD. For each analysis, cases were individuals who attained the later milestone or higher, and controls were those whose highest attainment was the immediately preceding level. This approach estimates genetic associations with progression at each conditional transition among individuals who have reached the preceding milestone, rather than associations with cumulative attainment across all prior milestones. After standard quality‐control (QC) filtering (e.g., imputation quality, minor allele frequency, Hardy–Weinberg equilibrium), we fit generalized linear mixed models with fastGWA-GLMM, adjusting for sex, birth year, genotyping batch, and PC1-PC10. Genome‑wide significance was set at two‑sided 𝑝 < 5 × 10^-8^. Calibration from LD score regression (LDSC) was acceptable across milestone transitions (intercepts ≈ 0.99-1.06; attenuation ratios ≈ 0.13-0.17 for the first three transitions, with Ratio < 0 at MSc → PhD; Table A in S2 Appendix), indicating that most inflation reflects polygenicity rather than residual stratification [33]. These transition-specific GWASs provided the inputs for the subsequent cross-trait LDSC r_g_ and h^2^_SNP_ analyses. For transition phenotypes, we report LDSC h^2^_SNP_ on the observed scale rather than applying a liability-scale transformation, because such transformations would require assumptions about population prevalences for conditional at-risk transitions. Genetic correlations (r_g_) are reported on the LDSC scale; because r_g_ is invariant to linear rescaling of the traits, these estimates do not require the same observed-to-liability transformation.

### Genetic correlations: Early transitions align with educational attainment and intelligence, while overlap attenuates under later conditional progression

**Fig 2:**
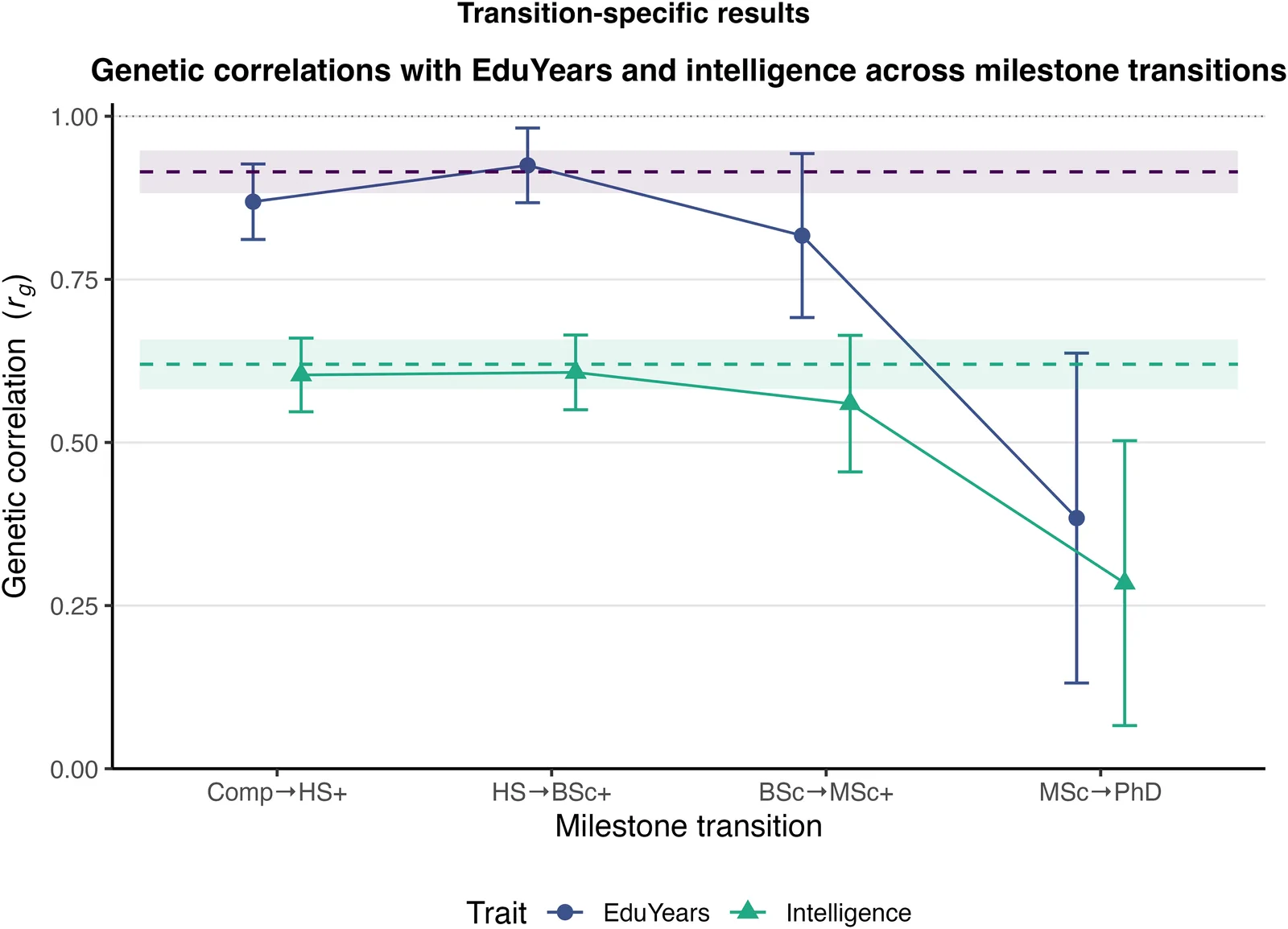
Genetic correlations between transition-specific milestone GWASs in MoBa and external GWAS meta-analyses of EduYears (EA4) and intelligence. LDSC genetic correlations (r_g_) between each transition-specific milestone GWAS (Comp→HS + , HS → BSc + , BSc → MSc + , MSc → PhD) and external GWAS meta-analyses of EduYears (EA4 [24]; navy circles) and intelligence (Savage et al., 2018 [26]; teal triangles). Error bars indicate 95% confidence intervals (CIs). Horizontal dashed lines with shaded 95% CI ribbons show benchmark correlations from the continuous MoBa EduYears GWAS, indicating the correlation of MoBa EduYears with EA4 (purple) and intelligence (Savage et al., 2018) (teal). The grey dashed line indicates r_g_ = 1.0 for reference. The plot shows high cross-trait genetic overlap at earlier milestone transitions and lower estimates at postgraduate transitions. Results using the intelligence GWAS from Lam et al. (2021) [27] are shown in Fig AA in S1 Appendix.

Building on these GWASs, cross-trait LDSC r_g_ estimates showed that transition-specific GWASs shared substantial genetic signal with broad measures of schooling (EA4 [24]) and intelligence (Savage et al., 2018) [26] up to the entry into higher education, with weaker observed overlap at postgraduate transitions (Fig 2). A sensitivity analysis using intelligence GWAS from Lam et al. (2021) [27] yielded nearly identical results across transitions, with the same early high-overlap pattern and attenuation at MSc → PhD (Fig AA in S1 Appendix).

To contextualize our findings, we first established reference benchmarks using the continuous EduYears GWAS in MoBa, serving as empirical ceilings for genetic overlap with EA4 [24] and intelligence (Savage et al., 2018) [26]. The r_g_ between MoBa EduYears and EA4 indicated near-complete overlap (≈ 0.93, 95% CI [0.90–0.96]). Similarly, the r_g_ between MoBa EduYears and intelligence (Savage et al., 2018) established the expected relationship between educational attainment and cognitive ability (≈ 0.63, [0.58–0.67]).

Against these benchmarks, early milestone transitions showed very high genetic overlap with EA4 (Comp→HS + : r_g_ ≈ 0.87 [0.81–0.93]; HS → BSc + : r_g_ ≈ 0.92 [0.87–0.98]) and substantial overlap with intelligence (Comp→HS + : r_g_ ≈ 0.60 [0.55–0.66]; HS → BSc + : r_g_ ≈ 0.61 [0.55–0.66]), a pattern that closely mirrored the benchmark relationships of MoBa EduYears with EA4 and intelligence. The pattern attenuated at postgraduate levels. BSc → MSc+ showed lower correlations with EA4 (r_g_ ≈ 0.82 [0.69–0.94]) and intelligence (r_g_ ≈ 0.56 [0.45–0.66]), though these remained substantial. The observed overlap was weakest for MSc → PhD, with lower correlations with both EA4 (r_g_ ≈ 0.38 [0.13–0.64]) and intelligence (r_g_ ≈ 0.28 [0.07–0.50]). These estimates were also markedly less precise, consistent with the substantially smaller effective sample size at MSc → PhD (N_eff_ = 8,185; Table 1). Because this transition is estimated within an already highly selected MSc+ at-risk group, the lower correlations should not be interpreted as showing that education- or intelligence-related traits matter less for doctoral attainment. Selection into this at-risk group may restrict the residual variation in education-related characteristics available to distinguish further progression.

**Table 1:** Sample sizes and effective sample sizes (*N*_eff_) for educational milestone analyses.

Cohort	Approach	Milestone	*N* (Cases)	*N* (Controls)	*N* (Total)	*P* (Case proportion)	*N*_eff_ (Effective Sample Size)
**NTR**	Cumulative	HS+	7,782	1,128	8,910	87.3%	3,941
		BSc+	4,770	4,140	8,910	53.5%	8,865
		MSc+	1,470	7,440	8,910	16.5%	4,910
		PhD	79	8,831	8,910	0.9%	313
**MoBa**	Cumulative	HS+	109,398	11,129	120,527	90.8%	40,406
		BSc+	73,188	47,339	120,527	60.7%	114,983
		MSc+	22,464	98,063	120,527	18.6%	73,109
		PhD	2,277	118,250	120,527	1.9%	8,936
**MoBa**	Transition –specific	Comp→HS+	109,398	11,129	120,527	90.8%	40,406
		HS → BSc+	73,188	36,210	109,398	66.9%	96,899
		BSc → MSc+	22,464	50,724	73,188	30.7%	62,276
		MSc → PhD	2,277	20,187	22,464	10.1%	8,185

The “*N* (Total)” column lists the full cohort size for cumulative outcomes and the at‑risk set (those who cleared the prior milestone) for transition‑specific outcomes. Effective sample size (*N*_eff_), a key indicator of statistical power for binary traits, is calculated as *N*_eff_ = 4/(1/*N*_cases_+1/*N*_controls_) = 4*NP*(1 − *P*) [60], where *P* is the case proportion. Because power scales with *N*_eff_, information is maximized near 50:50 case-control balance and declines as prevalence departs from 0.5. For the first milestone, cumulative (HS+) and transition‑specific (Comp→HS+) definitions coincide. In MoBa, cumulative definitions yield larger *N*_eff_ than transition-specific ones at BSc + , MSc + , and PhD (≈ + 18.7%, + 17.4%, + 9.2%), while NTR PhD has a very small *N*_eff_ (313), implying wide confidence intervals.

For EA4, formal block-jackknife comparisons showed that all three milestone-transition contrasts involving MSc → PhD were FDR-significant at q < 0.001, and HS → BSc+ also differed from BSc → MSc+ at q < 0.05. For intelligence, the same three milestone-transition contrasts involving MSc → PhD were FDR-significant under the Lam et al. (2021) [27] sensitivity reference (q < 0.01) (Fig AB in S1 Appendix and Tables D–F in S2 Appendix).

Supplementary analyses with additional external traits extended this pattern. LDSC analyses showed that the transition into higher education broadly followed the same attainment-related pattern for CogSub and NonCogSub, the GWAS-by-subtraction [28] components indexing cognitive and non-cognitive contributions to EA, whereas the educational-field PCs [29], indexing technical-versus-social and practical-versus-abstract propensities beyond educational level, showed a different profile across milestones (Figs AC–AF in S1 Appendix). Supplementary MiXeR [30] likewise highlighted the transition into higher education as the clearest overlap signal; among milestone transitions, HS → BSc+ yielded the most interpretable overlap decompositions, whereas Comp→HS + , BSc → MSc + , and MSc → PhD were less well identified and are therefore interpreted more cautiously. MoBa EduYears provided the more stable overall benchmark across reference traits in the supplementary MiXeR analyses (Figs AG–AK in S1 Appendix and Tables J–M in S2 Appendix).

### Observed‑scale SNP heritability follows an inverse‑U across milestone transitions

**Fig 3:**
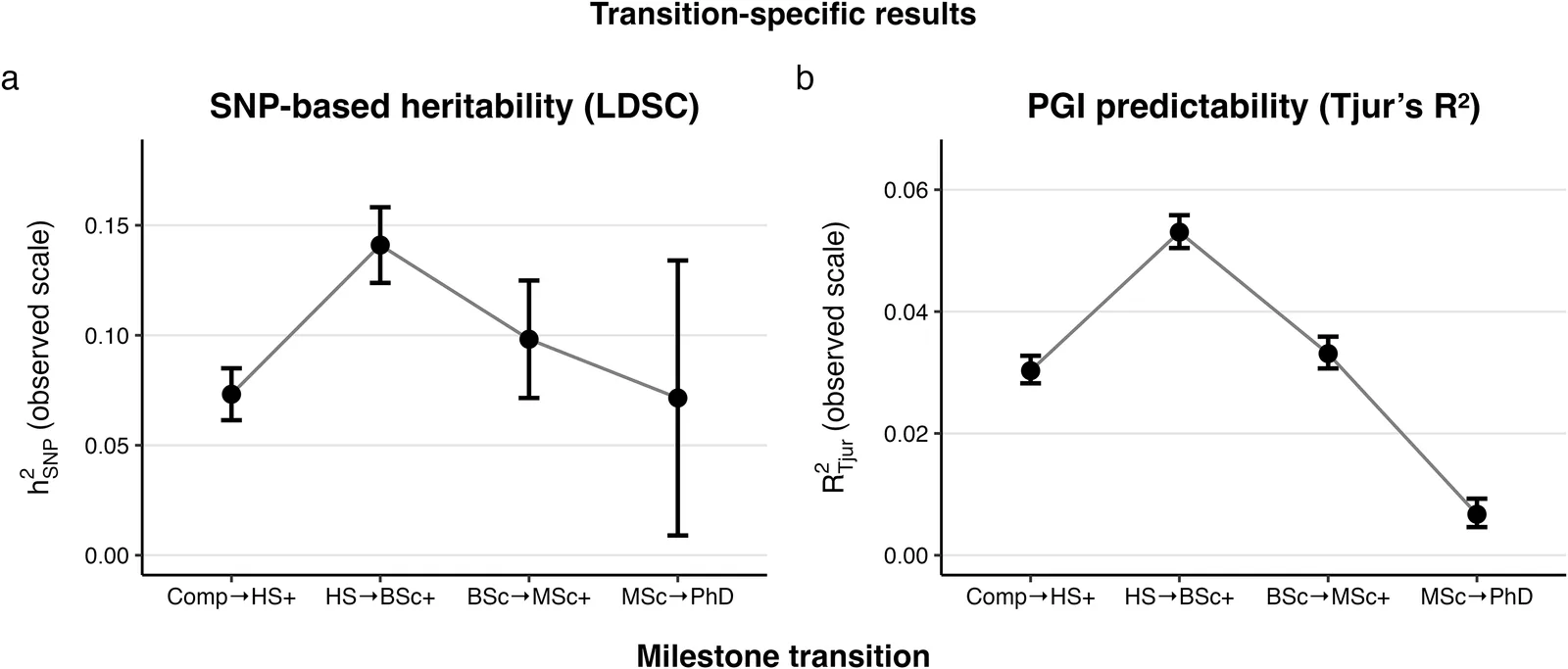
Common‑variant signal and PGI discrimination at successive educational transitions. **a,** Observed‑scale LDSC estimates of SNP‑based heritability (h^2^_SNP_) for each transition: Comp→HS + , HS → BSc + , BSc → MSc + , MSc → PhD. Points show estimates; vertical bars show 95% CIs. Observed-scale heritability is highest at HS → BSc+ and lower at postgraduate transitions. **b,** Predictive performance of an EA4‑derived polygenic index (PGI) for each transition, summarized by Tjur’s R^2^ from adjusted probit models; 95% bootstrap (1,000 resamples) CIs shown. Discrimination follows an inverse-U pattern, with a marked drop at MSc → PhD. Both panels show observed-scale quantities; no liability-scale transformation was applied to the transition outcomes.

Following the patterns of genetic overlap, the magnitude of the common-variant association signal also varied across milestone transitions. Observed-scale LDSC estimates of SNP-based heritability (h^2^_SNP_; see Methods) followed an inverse-U pattern (Fig 3A): estimates were modest for Comp→HS+ (h^2^_SNP_ ≈ 0.07 [0.06–0.09]), highest at HS → BSc+ (h^2^_SNP_ ≈ 0.14 [0.12–0.16]), and lower at the postgraduate transitions (BSc → MSc + : h^2^_SNP_ ≈ 0.10 [0.07–0.12]; MSc → PhD: h^2^_SNP_ ≈ 0.07 [0.01–0.13]).

### Polygenic indices stratify milestone progression, while predictive discrimination peaks at the transition into higher education

Extending these heritability insights to prediction, we evaluated how the EA4 PGI forecasts each milestone transition in MoBa (Fig 3B). Using probit regression adjusted for sex, birth year, and ten ancestry principal components, we quantified predictive power using Tjur’s coefficient of discrimination (R^2^_Tjur_) on the observed scale. The results followed an inverse-U pattern: predictability was moderate for Comp→HS+ (R^2^_Tjur_ ≈ 0.03), peaked for HS → BSc+ (R^2^_Tjur_ ≈ 0.05), and then waned for the postgraduate transitions (BSc → MSc+ ≈ 0.03; MSc → PhD < 0.01).

**Fig 4:**
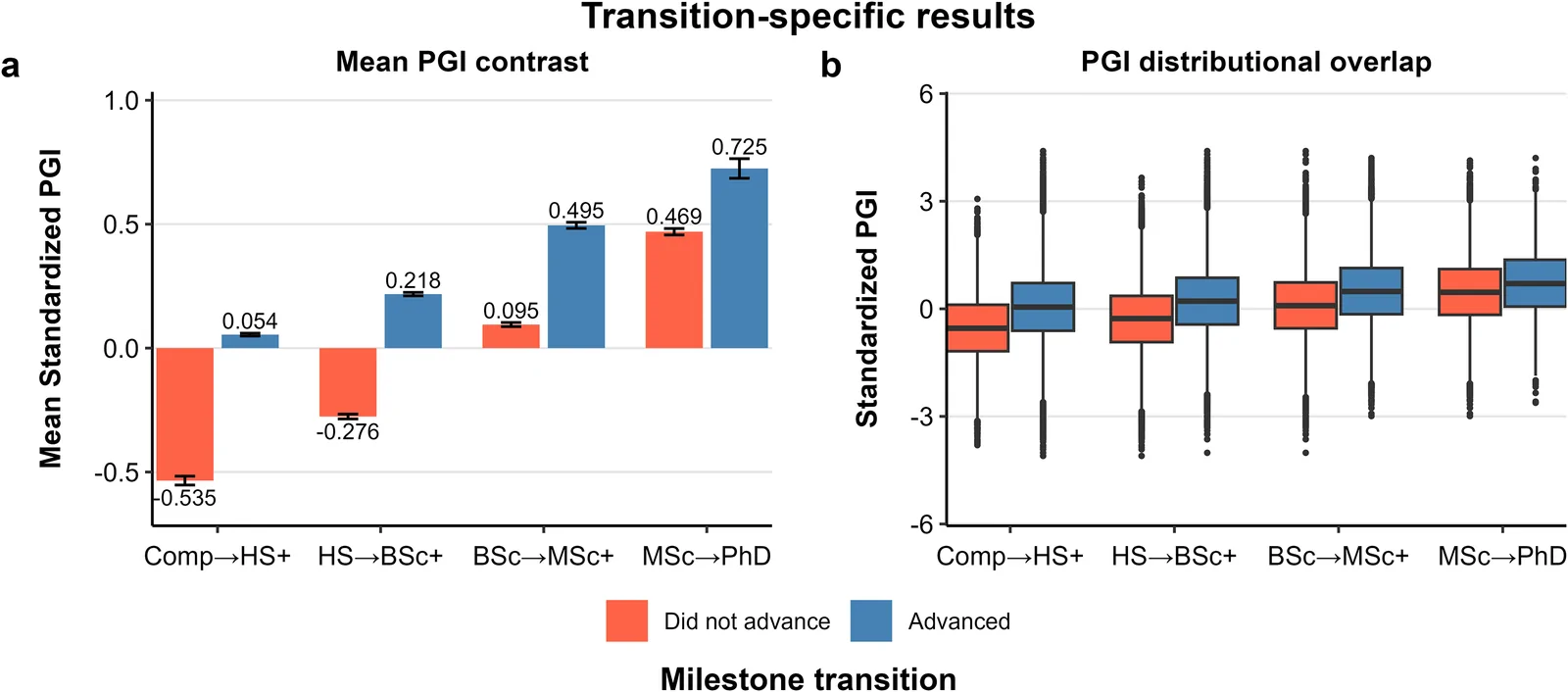
PGI differences at each educational milestone transition. **a, Mean PGI contrast.** Bars show the mean standardized PGI (z-score; overall MoBa mean = 0, s.d. = 1) for individuals who did not advance (orange) and those who advanced (blue) to each milestone. Error bars represent ± 1.96 s.e.; numeric labels indicate the group means. Advancing groups consistently had higher mean PGIs than non-advancing groups within each transition, but the between-group mean difference narrowed across successive transitions, from approximately 0.59 s.d. at Comp→HS+ to approximately 0.26 s.d. at MSc → PhD. **b, PGI distributional overlap.** Box plots show the full PGI distribution for the same groups. Panels a and b are complementary: panel a summarizes mean-level stratification, whereas panel b shows the considerable distributional overlap that remains even when group means differ.

Fig 4 provides further insight by contrasting mean PGI differences with the underlying score distributions at each milestone. This reveals a decoupling between average group separation and the PGI’s predictive performance in Fig 3B. Although the mean PGI was consistently higher among those who advanced at every milestone (Fig 4A), the score distributions for advancing and non-advancing groups nevertheless overlapped substantially, particularly at the PhD transition (Fig 4B). These results show that the PGI stratifies milestone progression at the group level, while retaining limited capacity to discriminate between individuals who will and will not advance to the next stage, especially at boundary milestones where case fractions are very high (Comp→HS+) or very low (MSc → PhD).

Supplementary PGI analyses broadly reinforced the same transition-specific pattern. For intelligence-related and GWAS-by-subtraction PGIs (Savage [26], Lam [27], CogSub and NonCogSub [28]), predictive increments peaked at HS → BSc + , and advancers had higher mean PGI values at every transition, although the between-group difference narrowed at later milestones. Mean PGI values nevertheless increased across transitions for both advancers and non-advancers, consistent with progressive selection into later at-risk sets, while the corresponding per-SD odds-ratio point estimates were generally lower at later transitions. By contrast, the educational-field PCs [29] showed directionally mixed mean contrasts and odds-ratio trajectories, consistent with these scores indexing opposing field-choice propensities rather than a unidirectional liability to educational advancement (Figs AL–AN in S1 Appendix; Table N in S2 Appendix).

### Cumulative genetic associations: Inter-milestone overlap and heritability comparisons

To complement the transition analyses, we re-estimated each milestone under a cumulative definition (Fig 1C), modeling genetic liability associated with reaching at least each level (HS + , BSc + , MSc + , PhD) by contrasting it with all lower levels in the full cohort. This design served three purposes. First, it enabled estimation of within-cohort pairwise genetic correlations among milestones using BOLT-REML in the full sample. Second, it allowed twin–molecular comparisons by reporting common-variant and twin-based heritabilities on the same liability scale. Third, it provided two complementary estimators of SNP heritability for robustness: LDSC from summary data and BOLT-REML from individual-level data. The GWAS pipeline matched the transition analyses (QC, fastGWA-GLMM, covariates, threshold). LDSC calibration was again acceptable (intercepts ≈ 1–1.08; attenuation ratios ≈ 0.04–0.17; Table A in S2 Appendix).

**Fig 5:**
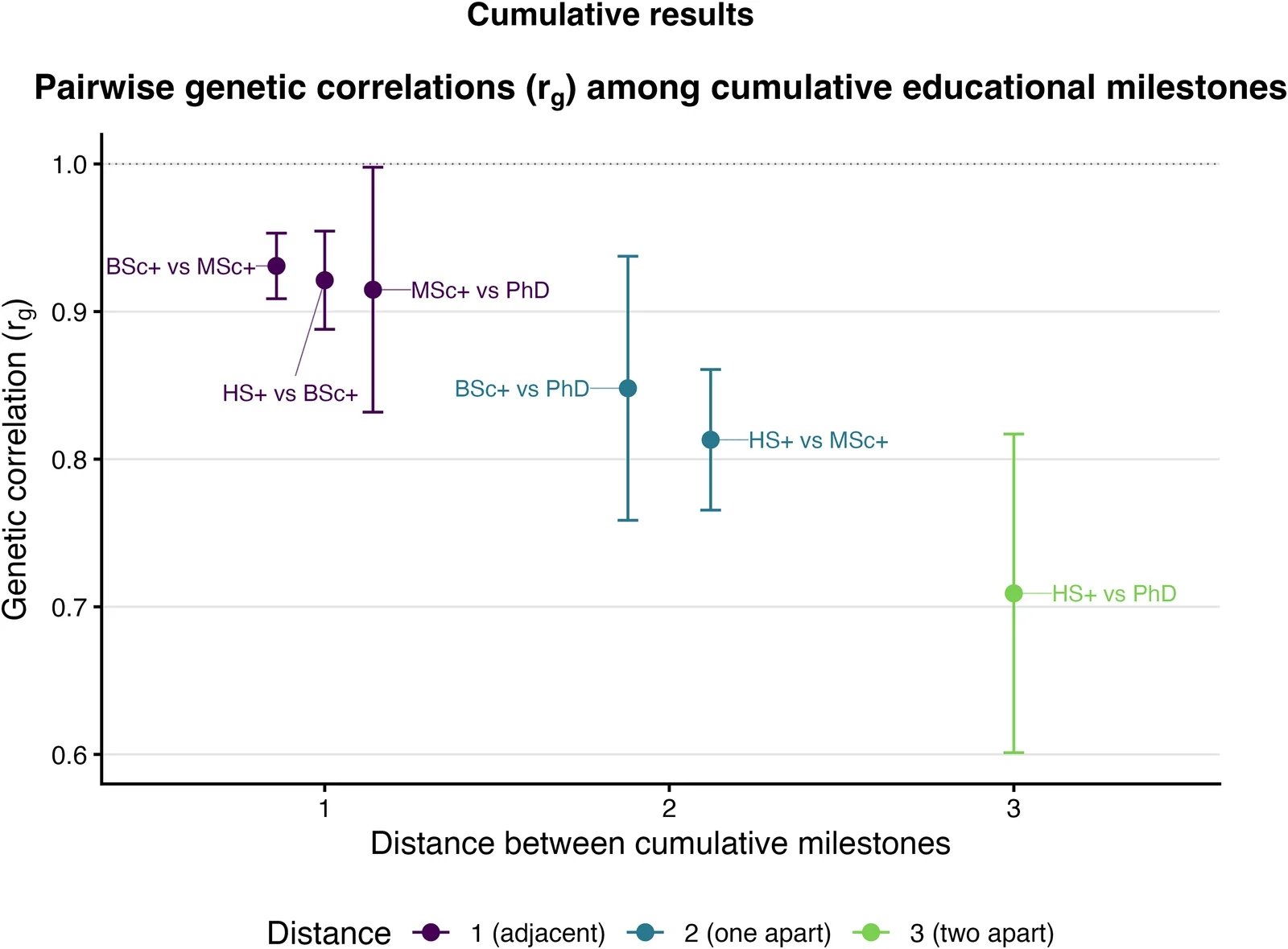
Pairwise genetic correlations (r_g_) among cumulative educational milestones in MoBa. Pairwise genetic correlations (r_g_) were estimated using a multivariate BOLT-REML model fitted jointly to the four cumulative milestones (HS + , BSc + , MSc + , and PhD). Points show r_g_ estimates with 95% confidence intervals. Color encodes the number of steps separating each pair and matches the x‑axis “distance”: 1 = adjacent (HS + vs BSc + , BSc + vs MSc + , MSc + vs PhD), 2 = one step apart (HS + vs MSc + , BSc + vs PhD), and 3 = two steps apart (HS + vs PhD). The dashed horizontal line marks r_g_ = 1. Genetic overlap is highest for adjacent milestones and lower for more distant milestone comparisons.

Using BOLT-REML, genetic correlations among cumulative milestones (Fig 5) were highest for adjacent steps (e.g., HS + vs BSc + , r_g_ ≈ 0.92) and lower for more distant comparisons (e.g., HS + vs PhD, r_g_ ≈ 0.71). This gradient indicates substantial shared genetic liability across cumulative milestones, with lower overlap as educational distance increases.

#### Twin–SNP heritability gap is largest at high-school completion and narrows at higher degrees.

**Fig 6:**
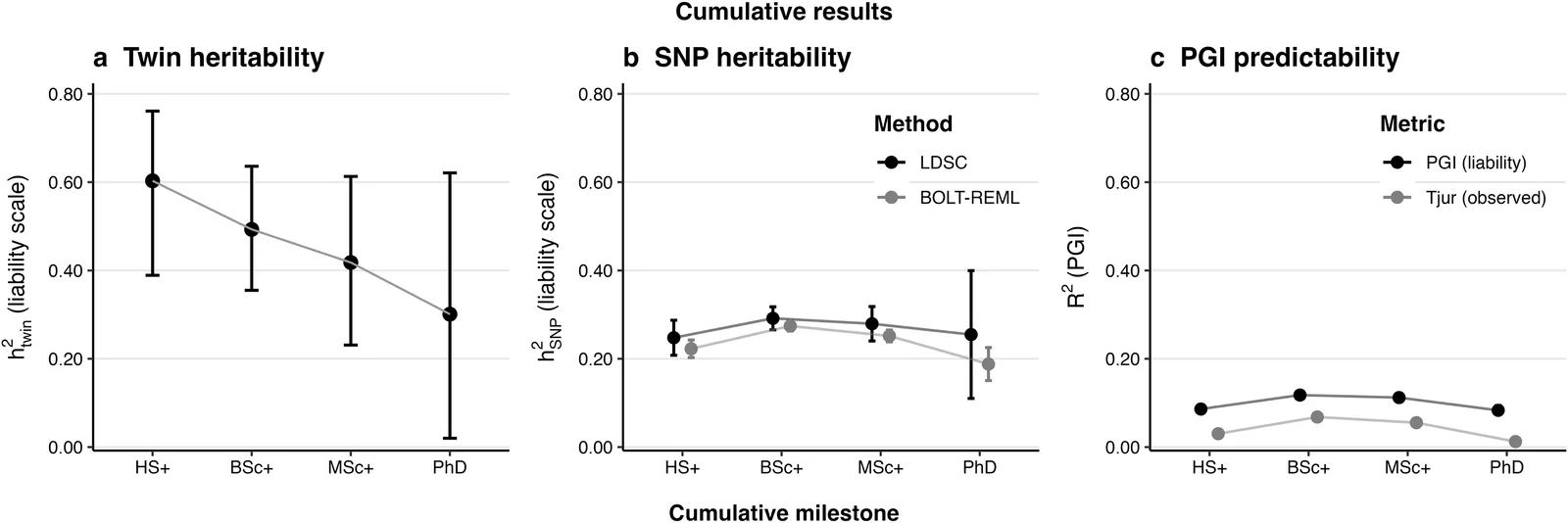
Cumulative genetic associations across educational milestones. **a, Twin heritability.** Additive twin heritability (h^2^_twin_) estimated with Bayesian liability‑threshold ACE models from the Norwegian Twin Registry; points show posterior means and whiskers show 95% credible intervals on the liability scale. **b, SNP heritability.** Common‑variant heritability (h^2^_SNP_) estimated by LDSC (black) and BOLT‑REML (grey); whiskers show 95% confidence intervals on the liability scale. **c, PGI predictability.** Two complementary metrics for the EA4‑based polygenic index. PGI (liability) is the variance on the liability scale explained by the PGI derived from the probit slope with a standardized residualized PGI following Lee et al. [34]. Tjur (observed) is Tjur’s coefficient of discrimination (R^2^_Tjur_) from probit models on the observed scale. Whiskers show 95% bootstrap CIs from 1,000 resamples for both metrics. Across panels, twin heritability point estimates decrease from HS+ to PhD, whereas SNP heritability and PGI predictability follow an inverse-U pattern, with higher point estimates around BSc+ and lower estimates at MSc+ and PhD.

To estimate the broader additive genetic contribution to each cumulative milestone, we analyzed data from 8,910 twins in the Norwegian Twin Registry. Additive twin heritability (h^2^_twin_) from Bayesian liability‑threshold ACE models (Fig 6A) was highest for HS+ (≈ 0.60, 95% credible interval [0.39, 0.76]) and declined in point estimate for BSc+ (≈ 0.49, [0.36, 0.64]), MSc+ (≈ 0.42, [0.23, 0.61]), and PhD (≈ 0.30, [0.02, 0.62]). Although intervals widened at rarer milestones, especially PhD, the point estimates followed a monotonic decline. Environmental components showed contrasting patterns: the shared environment (C) followed an inverse‑U shape, peaking around BSc+ and MSc+ (both ≈ 0.31), whereas the unique environment (E), which also includes measurement error, showed a U‑shape, lowest at BSc+ (≈ 0.20) and highest at PhD (≈ 0.47). Full ACE results are provided in Table C in S2 Appendix and Fig Z in S1 Appendix.

Using identical cumulative contrasts in MoBa, we then estimated common‑variant SNP heritability (h^2^_SNP_) with two complementary methods: summary‑level LDSC and individual‑level BOLT‑REML (Fig 6B). LDSC uses summary statistics and its intercept helps assess inflation from confounding, whereas individual-level REML is typically more statistically efficient; agreement between the two therefore increases confidence in the estimates. To permit direct comparison with twin‑based heritability (h^2^_twin_), observed‑scale SNP-based heritability estimates were converted to the liability scale (see Methods). Both methods showed a similar inverse‑U pattern: values increased from HS+ (≈ 0.25 LDSC; ≈ 0.22 BOLT‑REML) to a peak at BSc+ (≈ 0.29; ≈ 0.27), and were lower for MSc+ (≈ 0.27; ≈ 0.25) and PhD (≈ 0.25; ≈ 0.19).

Finally, we assessed the predictability of an EA4‑based PGI for the cumulative milestones using two complementary metrics (Fig 6C). First, we report the variance explained on the liability scale, which enables a direct comparison with our heritability estimates, derived from the probit slope with a standardized residualized PGI following Lee et al. [34]: HS+ ≈ 0.09, BSc+ ≈ 0.12, MSc+ ≈ 0.11, and PhD ≈ 0.08. Second, we report Tjur’s R^2^, which captures discrimination on the observed scale: HS+ ≈ 0.03, BSc+ ≈ 0.07, MSc+ ≈ 0.06, and PhD ≈ 0.01. Both metrics peaked near the bachelor’s level and were lower for MSc+ and PhD.

Comparing these estimates shows method-specific patterns across cumulative milestones (Fig 6). Twin heritability point estimates decreased from HS+ to PhD, whereas both SNP heritability and PGI predictability followed an inverse-U pattern, peaking at BSc + . The gap between twin and SNP heritability estimates was largest at HS+ and narrower for subsequent milestones.

## Discussion

Educational attainment is a sequential credentialing process and, at the same time, an institutional sorting process. In the Norwegian system, individuals move from compulsory schooling to high-school completion, then to bachelor’s, master’s, and doctoral credentials, with each step shaped by institutional rules, grading practices, admissions criteria, field availability, and the allocation of funded places. These gates define who is eligible for subsequent progression and structure the comparison groups observed at each milestone. Conventional EduYears GWAS have been productive precisely because they harmonize heterogeneous educational systems into a single quantitative phenotype, but this aggregation is not designed to distinguish where in the sequence genetic associations are expressed or how institutional sorting shapes them. An EduYears GWAS estimates one aggregate genome-wide SNP-association profile for a years-based phenotype rather than separate profiles for progression through distinct milestones. Using that aggregate profile to represent each milestone contrast entails two simplifying assumptions. The first is qualitative uniformity, meaning that the same genome-wide pattern of SNP associations is shared across milestones. The second is quantitative uniformity, meaning that the magnitude of those associations is constant, or differs only by a common scaling factor, across the EduYears metric. Our milestone framework estimates these profiles separately, allowing their composition, direction, and magnitude to vary across milestones. We first consider the empirical patterns relevant to composition and magnitude, and then examine how external measurement and selection shape their interpretation.

Several findings are relevant to the composition of the observed association profiles. In the cumulative framework, internal genetic correlations were highest among adjacent milestones and lower for more distant comparisons, revealing a distance gradient in estimated genetic overlap across cumulative milestone definitions. In the transition-specific framework, external genetic correlations with EA4, intelligence, and the GWAS-by-subtraction cognitive and non-cognitive components showed broadly similar attenuation at later transitions. The EduFields axes followed distinct, construct-specific trajectories. PC2, where higher values index practical fields and lower values index abstract fields, shifted toward the abstract end of the axis at later milestones. PC1, where higher values index technical fields and lower values index social fields, showed a more transition-dependent pattern without simple monotonic enrichment. Taken together, these results show that a scalar EduYears metric collapses distinct patterns of selection, prediction, and field composition across the educational sequence.

The strength of the observed signal also varied across milestone definitions. Under the transition-specific framework, both observed-scale h^2^_SNP_ and EA4 PGI discrimination followed an inverse-U pattern, with the largest common-variant signal and strongest discrimination at HS → BSc+ and lower estimates at the first and postgraduate transitions. This shape was not specific to the EA4 PGI. Supplementary analyses showed similar inverse-U trajectories in incremental Tjur’s R^2^ for intelligence-related, GWAS-by-subtraction, and EduFields PGIs (Fig AL in S1 Appendix). Under the cumulative framework, SNP heritability and PGI predictability were flatter but still tended to be highest around BSc + , whereas twin heritability point estimates declined from HS+ to PhD. Together, the transition-specific and cumulative analyses show that the magnitude and predictive utility of the observed genetic signal depend on milestone definition, target estimand, and analytical framework.

These composition and magnitude patterns can arise from several sources, including external phenotype coding, case composition, and sequential selection. Selection is particularly relevant to the transition-specific estimates because later contrasts are defined within progressively selected at-risk groups. We first consider whether limitations of the external EA4 benchmark could account for the pattern, and then turn to selection within the educational sequence.

The publicly available EA4 subset excludes 23andMe, is heavily weighted toward UK Biobank’s binary degree item, and uses ISCED-1997 coding that collapses bachelor’s and master’s degrees into a single category (Note A in S1 Appendix) [35]. These features may affect the magnitude of EA4-based genetic correlations and PGI estimates, particularly at postgraduate transitions. EA4 and intelligence should also be treated as overlapping rather than orthogonal reference traits, given their strong phenotypic and genetic coupling [36,37]. A further limitation is that the UK Biobank fluid-intelligence component used in Savage et al. (2018) [26] was adjusted for socioeconomic status using the Townsend deprivation index. Because the Lam et al. (2021) [27] intelligence reference incorporates GWAS inputs overlapping with Savage et al., the similar trajectories obtained with Savage and Lam do not provide an independent check on potential bias arising from this phenotypic conditioning. Nevertheless, two features of our results make it unlikely that EA4 coding alone accounts for the full cross-milestone pattern. First, the intelligence GWAS does not inherit EA4’s credential coding, although it is not independent of education-related and socioeconomic signal, yet its transition-specific correlations attenuate at the same later transitions. Second, the milestone-specific h^2^_SNP_ estimates are derived from the milestone GWASs themselves and follow a similar inverse-U pattern without using EA4 summary statistics. The GWAS-by-subtraction cognitive and non-cognitive components were constructed jointly from the EA3 educational-attainment and cognitive-performance GWASs, both reported by Lee et al. (2018), and therefore do not provide a comparison fully independent of the EduYears GWAS lineage underlying EA4. Among the more interpretable supplementary MiXeR analyses, HS → BSc+ showed particularly high overlap with EA4, consistent with the public EA4 subset being strongly aligned with entry into higher education, although transition-specific identification was otherwise limited. External measurement therefore affects the interpretation of EA4 benchmark magnitudes but is unlikely to account for the complete within-MoBa pattern.

Selection and case composition are particularly relevant to the transition-specific estimates. At the boundary milestones, case fractions are far from balance but in opposite directions. High-school completion is near-universal, so non-completers are rare, whereas doctoral completion is rare, so advancers are few. These imbalances can reduce observed-scale association signal and predictive discrimination. The transition-specific framework adds repeated conditioning on prior attainment. Later analyses therefore compare advancing and non-advancing groups drawn from increasingly selected at-risk sets, with less residual variation in education-related characteristics available to distinguish them.

The changing composition of these at-risk sets is visible in the supplementary PGI analyses. For the intelligence-related, CogSub, and NonCogSub PGIs, mean scores increased across transitions among both advancers and non-advancers, indicating that later comparisons are drawn from groups increasingly enriched for education-related, cognitive, and non-cognitive genetic propensities (Fig AM in S1 Appendix). Mean PGI, Tjur’s R^2^, and the per-SD odds ratio capture different features of these selected groups. Mean PGI describes the composition of the at-risk set. Tjur’s R^2^ quantifies observed-scale discrimination between advancers and non-advancers within that set. The odds ratio estimates the conditional association of a one-SD higher PGI with advancement. These quantities should therefore not be interpreted interchangeably.

For the intelligence-related and GWAS-by-subtraction PGIs, the empirical odds ratios also tended to attenuate across later transitions (Fig AN in S1 Appendix; Table N in S2 Appendix). This pattern need not reflect range restriction alone. Conditioning on prior attainment can induce dependence between a measured PGI and unmeasured genetic, environmental, and social contributors to progression because attainment is a common consequence of all of them. Individuals who reached a milestone despite a lower measured PGI may, on average, carry more of these unmeasured contributors. Such collider-like dependence can alter, and potentially attenuate, the observed per-SD association within later at-risk sets. The empirical odds-ratio trajectories are therefore compatible with selection-induced dependence within later at-risk sets and do not, by themselves, imply that the underlying PGI effect changes across milestones. Read together, the weaker predictive performance, lower external genetic correlations, and reduced observed-scale signal at the latest milestones should not be interpreted as showing that education-related, cognitive, or non-cognitive propensities matter less for doctoral attainment. They are compatible with later at-risk sets already being enriched on related propensities, leaving less residual variation with which to distinguish who advances further.

Note D in S1 Appendix illustrates this point directly for transition-specific PGI discrimination. With the simulated PGI coefficient held constant across transitions, sequential thresholding raises mean PGI and reduces PGI variance in later at-risk groups. Together with endpoint case imbalance, this lowers Tjur’s R^2^, while the per-SD odds ratio remains approximately stable (Figs AR–AS in S1 Appendix). The simulation is an illustrative scenario rather than a model fitted to the MoBa data, and it does not directly model genetic correlations or h^2^_SNP_. The approximately stable odds-ratio trajectory is a feature of this particular simulation and should not be interpreted as a general prediction of constant-coefficient selection processes; more realistic forms of sequential selection could alter conditional odds ratios without any change in the underlying PGI coefficient. The simulation’s central implication is therefore narrower: declining transition-specific PGI discrimination can occur even when the underlying PGI coefficient is constant across transitions. Related conditioning and case-composition concerns also bear on the interpretation of transition-specific genetic correlations and observed-scale h^2^_SNP_, although those quantities are not directly evaluated by the simulation.

The treatment of parental socioeconomic status raises a related estimand question. We did not treat parental SES as a straightforward confounder to be removed by default because family background is part of the developmental and institutional context through which genetic associations with education are expressed. Parental education, income, and occupation may partly reflect parental traits that are themselves genetically influenced. Through inheritance, assortative mating, dynastic effects, and genetic nurture, these traits can correlate with offspring genotype and shape the environment in which educational progression occurs. Adjusting for parental SES could therefore remove associations operating through family-mediated pathways rather than simply reduce confounding. Our primary specification estimates genotype-milestone associations without conditioning on parental SES, thereby retaining associations operating through or alongside family-mediated and socially structured pathways. SES-adjusted analyses may be useful as sensitivity or mechanism analyses, but they target a different estimand and were not treated as the default specification.

For the cumulative milestones, the point-estimate gap between twin-based and SNP-based heritability was largest at HS+ and narrower thereafter. Possible contributors include genetic variation not well tagged by common SNPs, such as rare or structural variants, and standard twin-model assumptions that may be imperfectly met [38–40]. However, the twin and SNP estimates were obtained in different cohorts. Their difference may therefore also reflect cohort variation in age, sampling, milestone prevalence, and other population characteristics rather than estimation method alone. We consequently treat this as a descriptive point-estimate gap rather than a pure within-cohort decomposition. Taken at face value, the pattern is consistent with current common-variant methods capturing a smaller share of the broader biometric additive signal at the near-universal HS+ threshold than at later milestones, but the present data do not isolate the source of that difference.

The practical implications concern how education PGIs should be used and interpreted. A years-based PGI is not uniformly predictive across milestone definitions. Its discrimination was strongest for intermediate transitions, particularly entry into higher education, and weaker at the distributional endpoints. Even where group means differed, the score distributions overlapped substantially. These findings support the use of education PGIs for population-level description and hypothesis testing, but not for individual-level differentiation. They also show that PGI performance is context-dependent and shaped by phenotype definition, case composition, and prior selection.

A related implication concerns the EduYears phenotype itself. EduYears is a harmonized credential-coded index rather than a literal measure of time exposed to education. In the Norwegian system, the years assigned to compulsory, upper-secondary, and tertiary credentials are placed on the same numerical scale even though they represent different institutional settings and selection processes. Our analyses begin after compulsory education and therefore do not resolve year-to-year heterogeneity within primary or lower-secondary schooling or within multi-year credentials. Nevertheless, the post-compulsory milestone contrasts differed substantially in prevalence, selection structure, predictive discrimination, and genetic association patterns. EduYears estimates average across these heterogeneous contrasts, with each contributing according to its coding, prevalence, and covariance with the overall index.

Scale also matters for interpretation. Observed-scale h^2^_SNP_, PGI R^2^, and regression coefficients can depend on phenotype coding and distribution and are not automatically comparable with liability-scale estimates for explicitly thresholded outcomes. A recent reanalysis of Estonian data illustrates this point: a difference in observed-scale EduYears h^2^_SNP_ became smaller and was no longer statistically significant after attainment was recoded as university-degree status and transformed to the liability scale [41,42], although the binary recoding also discarded information. In the present study, transition-specific estimates are conditional, observed-scale descriptions of progression within the relevant at-risk set, whereas cumulative milestone phenotypes are defined in the full cohort and can be placed on the liability scale for twin-SNP and cross-cohort attained-status comparisons. Coefficients described as associations “per additional year of education” are therefore better understood as associations per unit increase in a harmonized credential-coded index, not as stage-invariant associations with one calendar year anywhere in the educational sequence.

Several limitations should be considered. Residual right-censoring for advanced degrees is possible, particularly in the younger NTR sample, which had a mean age of 40.9 years, although it is less likely in the older MoBa cohort, which had a mean age of 51.4 years. Estimates for doctoral milestones are less precise because doctoral attainment is rare, resulting in smaller effective sample sizes and wider intervals. External comparisons inherit the measurement structure and sample composition of the publicly available EA4 data. Twin estimates depend on standard biometric assumptions, and comparisons between twin and SNP estimates also involve different cohorts. The analyses are associational and do not identify causal genetic, biological, or social pathways. As discussed above, prior selection can alter the conditional associations estimated at later transitions by changing the composition and residual variation of the at-risk groups. The present design cannot determine how much of the observed attenuation is attributable to this selection process. Finally, the findings reflect the Norwegian educational system and a primarily European-ancestry population. A lay-oriented discussion of interpretation, limitations, and potential misuse is provided in Note C in S1 Appendix.

Several extensions could strengthen the milestone-preserving framework. Larger analyses across multiple cohorts would improve precision at high-school non-completion and doctoral attainment, enlarge the MSc+ at-risk set, and enable out-of-sample validation of milestone-specific PGIs. Applying the same framework across educational systems and ancestry groups would also show how institutional features such as financial support, tuition, admissions selectivity, field availability, and funded doctoral places modify milestone-specific patterns. Broader reference traits, including personality and psychiatric or neurodevelopmental liabilities [43–47], could test whether the observed trajectories are specific to attainment-related constructs or extend to other domains.

Whole-genome sequencing could test whether rare or structural variation contributes to the HS+ twin-SNP point-estimate gap or to residual variation at the educational endpoints [48,49]. Family-based and multivariate designs are especially relevant to the interpretation of selection. Within-family milestone GWASs and PGI analyses could reduce confounding from shared family background, dynastic effects, assortative mating, and population structure [50–52]. Multivariate common-factor and heterogeneity models could test whether a broad educational-liability factor accounts for associations across cumulative and transition-specific milestones or whether residual milestone-specific patterns remain [53,54]. Such models could characterize the broad shared component and any residual milestone-specific associations more explicitly than the observed contrasts alone. They would not eliminate selection, and any residual pattern would still need to be interpreted in light of it.

Across the milestone analyses, common-variant signal and PGI discrimination were strongest around entry into higher education, cumulative genetic overlap was lower for more distant milestones, and the twin–SNP point-estimate gap was largest at high-school completion. Together, these findings show that genetic estimates depend on the educational contrast and comparison group. The attenuation observed in the transition-specific analyses is compatible with sequential selection induced by educational progression; milestone-specific changes in underlying genetic effects remain possible, but are neither demonstrated by nor necessary to explain this pattern. Because EduYears converts distinct credentials and progression steps into a single numerical scale, milestone-preserving analyses make this hidden structure visible and provide a clearer basis for interpreting genetic studies of education.

## Methods

### Ethics statement

This study used data from the Norwegian Mother, Father and Child Cohort Study (MoBa), the Norwegian Twin Registry (NTR), and educational register data from Statistics Norway (SSB). The current study was approved by the Regional Committees for Medical and Health Research Ethics (ref. 2017/2205). The University of Oslo was responsible for the study’s data-handling arrangements and conducted a Data Protection Impact Assessment in collaboration with the Norwegian Agency for Shared Services in Education and Research (Sikt; ref. 962088). Participation in MoBa is voluntary and based on written informed consent. The NTR is a consent-based registry in which participants provided written consent for health-related research. Data were provided to the authors as pseudonymized research extracts by the data controllers and were analyzed within the University of Oslo’s secure environment; only aggregate, non-identifiable results left the secure environment.

### Data samples

#### The Norwegian Mother, Father and Child Cohort Study (MoBa).

Our primary discovery analyses were conducted in the Norwegian Mother, Father and Child Cohort Study (MoBa), a population-based pregnancy cohort study conducted by the Norwegian Institute of Public Health [55,56]. Participants were recruited nationwide from 1999-2008 from 50 of Norway’s 52 hospitals with maternity units (41% participation rate), comprising approximately 114,500 children, 95,200 mothers, and 75,200 fathers. Participation required the ability to read Norwegian, as all materials were provided only in Norwegian.

This study analyzed 120,527 participants with registry-linked educational data: 48,953 males and 71,574 females born between 1937 and 1992 (males: 1937–1989; females: 1954–1992; mean birth year 1972.29 ± 5.63 for males, 1974.42 ± 5.08 for females). The mean age of the sample at data extraction (January 2025) was 51.4 ± 5.4 years.

Genotyping was conducted through multiple research projects using various arrays and genotyping centers. Quality control (QC) and imputation followed the MoBaPsychGen pipeline [57], which handled relatedness genetically rather than by reported family status alone. For the present analyses, we restricted the data to MoBa parents. Duplicate samples had been removed in the upstream QC pipeline, and any remaining related individuals in the adult analytic subset were retained and accounted for in the association analyses using mixed models with a sparse GRM.

Educational attainment (EA) distribution was: 9.2% (N = 11,129) compulsory education (Comp), 30.0% (N = 36,210) high school (HS), 42.1% (N = 50,724) bachelor’s (BSc), 16.7% (N = 20,187) master’s (MSc), and 1.9% (N = 2,277) doctorate (PhD). Sex-specific distributions showed males with higher Comp (M: 10.9%, F: 8.1%) and HS (M: 38.5%, F: 24.3%) completion, while females achieved higher BSc rates (M: 31.5%, F: 49.3%). MSc rates were similar (M: 16.9%, F: 16.6%), with males slightly higher for PhD (M: 2.2%, F: 1.6%).

The full distribution of EA across birth-year bins (5-year intervals) and sex in the MoBa sample is shown in Fig AO in S1 Appendix.

#### The Norwegian Twin Registry (NTR).

To estimate twin-based heritability, we used data from the Norwegian Twin Registry (NTR), a consent-based registry maintained by the Division for Health Data and Digitalization at the Norwegian Institute of Public Health. Our analytical sample was restricted to twins born 1967–2000 with available educational-attainment data. Zygosity was determined by questionnaire methods inquiring about twin similarity and has been verified in subsamples by genetic markers and DNA analysis [58].

The analytical sample included 8,910 twins: 3,054 males (34.3%) and 5,856 females (65.7%), with mean birth years of 1982.83 (SD = 10.41) and 1984.70 (SD = 10.38), respectively. At the time of data extraction (January 2025), the mean age was 40.9 ± 10.4 years. The sample contained 5,096 monozygotic (MZ) twins (57.2%) and 3,814 dizygotic (DZ) twins (42.8%).

EA distribution was: 12.7% (N = 1,128) Comp, 33.8% (N = 3,012) HS, 37.0% (N = 3,300) BSc, 15.6% (N = 1,391) MSc, and 0.9% (N = 79) PhD. Sex-specific distributions showed similar completion of Comp (M: 12.7%, F: 12.6%), but males completed HS more frequently (M: 38.5%, F: 31.4%), while females earned BSc more often (M: 30.2%, F: 40.6%). Males attained higher rates of MSc (M: 17.4%, F: 14.7%) and PhD (M: 1.2%, F: 0.7%).

The full distribution of EA across birth-year bins (5-year intervals) and sex in the NTR sample is shown in Fig AP in S1 Appendix.

### Definition of educational milestones and analytical approaches

EA was classified into five primary categories based on the Norwegian Standard Classification of Education (NUS2000 [59]), as detailed in Fig AQ in S1 Appendix. From these categories, we defined four key educational milestones: attaining at least a high school diploma (HS+), a bachelor’s degree (BSc+), a master’s degree (MSc+), and a doctoral degree (PhD).

**Fig 7:**
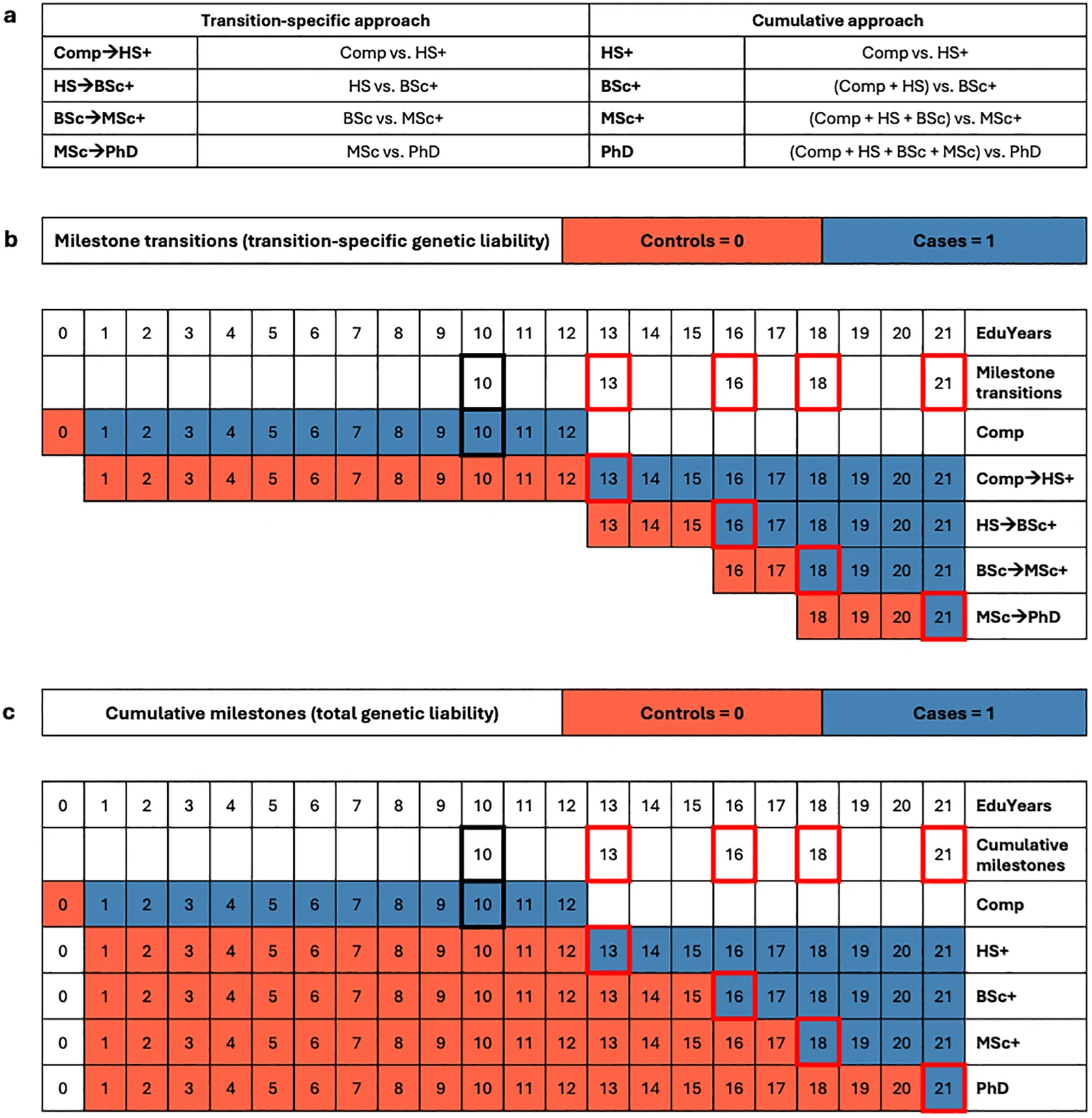
Definitions of transition-specific and cumulative analytical approaches. **a**, A summary of the case-control definitions for each educational milestone under the two primary analytical frameworks used in the study. **b**, Visual illustration of the transition-specific approach, which defines conditional progression contrasts. Cases (blue) for each milestone are compared against controls (red) who achieved only the immediately preceding educational level. In Fig 1B, these transition-specific contrasts are illustrated conceptually as advancement versus non-advancement, and Fig 4 visualizes the same underlying group contrast empirically. The black-outlined box marks the EduYears cut point corresponding to compulsory education (Comp), whereas the red-outlined boxes mark the higher cut points used to map EduYears to HS + , BSc + , MSc + , and PhD. These illustrative EduYears-to-milestone correspondences are anchored to the Norwegian educational system. **c**, Visual illustration of the cumulative approach, which defines attained-status contrasts in the full cohort. Cases (blue) are compared against all individuals who did not achieve that milestone, regardless of their final attainment. In Fig 1C, these same cumulative groups are illustrated conceptually as individuals who attained versus did not attain the milestone. The same black and red outlined boxes indicate how the milestone thresholds map onto the EduYears scale.

To analyze genetic associations with these milestones, we employed two complementary analytical frameworks: a transition-specific approach and a cumulative approach, both illustrated in Fig 7. This distinction allows us to distinguish genetic associations with conditional educational progression from genetic associations with cumulative attained status.

The transition-specific approach estimates genetic associations with each educational milestone transition, conditional on having reached the preceding milestone. As illustrated in Fig 7B, this framework defines cases as those who advance to a new milestone, while controls are limited to those whose highest attainment is the immediately prior level. We denote a milestone transition as X → Y, where cases have reached level Y or higher and controls are individuals whose highest attainment is X. For example, in the transition to a bachelor’s degree (HS → BSc+), cases have a BSc or higher, while controls completed high school (HS) but not a BSc. The transition-specific BSc → MSc+ analysis treats postgraduate advancement as attainment of either a master’s degree or a PhD, relative to stopping at the bachelor’s level.

In contrast, the cumulative approach estimates genetic associations with attaining a given educational milestone in the full cohort. As shown in Fig 7C, this framework compares all individuals who attained a specific milestone or higher (cases) against everyone in the sample who did not reach that level (controls). For example, in the cumulative analysis of a bachelor’s degree (BSc+), the control group includes all individuals with either compulsory education (Comp) or a high school diploma (HS). The cumulative MSc+ phenotype treats both master’s and doctoral attainment as cases. The specific sample sizes and effective sample sizes (N_eff_) for each analysis are detailed in Table 1.

### Statistical analyses

#### Overview.

Our analytical strategy leverages two Norwegian cohorts to characterize genetic associations with educational milestones through two complementary frameworks, with additional analyses used to triangulate the findings.

First, under a transition-specific framework, we estimated genetic associations with each sequential step conditional on prior attainment. To establish empirical ceilings for genetic overlap, we began by conducting an EduYears GWAS in MoBa and correlating it with EA4 [24] and the primary intelligence reference (Savage et al., 2018) [26]. With these benchmarks established, we conducted our transition-specific milestone GWASs. We used these results to estimate SNP-based heritability (h^2^_SNP_), calculate genetic correlations (r_g_) with EA4 and intelligence, and test the predictive power of an EA4-derived polygenic index (PGI). Supplementary analyses extended this framework to an intelligence sensitivity reference (Lam et al., 2021) [27], GWAS-by-subtraction cognitive and non-cognitive traits [28], educational-field axes [29], MiXeR [30] overlap decomposition, lead-locus overlap annotation [31], and external-trait PGIs.

Second, under a cumulative framework, we conducted a separate set of milestone GWASs to estimate genetic associations with attaining each status in the full cohort. This approach enabled us to assess genetic overlap (r_g_) among the milestones themselves using BOLT-REML, and to estimate common-variant heritability (h^2^_SNP_) with two complementary methods, summary-level LD score regression (LDSC) and individual-level BOLT-REML. Finally, this framework allowed us to contrast common-variant heritability with broader biometric additive heritability (h^2^_twin_) from classical twin modeling in the NTR. Together, these complementary approaches distinguish genetic associations with conditional educational progression from genetic associations with cumulative attained status.

#### Genome-wide association studies (MoBa).

We conducted genome-wide association studies (GWAS) in MoBa for EduYears and four educational milestones, with the milestones analyzed using both transition-specific and cumulative definitions (see Fig 7 for illustration of definitions and Table 1 for the primary and effective sample sizes). We also performed additional exploratory GWAS using non-cumulative contrasts, which directly compared individuals whose highest attainment was exactly BSc, MSc, or PhD with those whose highest attainment was exactly HS (see Table O in S2 Appendix for the full phenotype definitions and sample-size summaries). The MoBa EduYears GWAS provided essential benchmarks for establishing reference r_g_ estimates with external meta-analyses, as described in our Statistical Analyses overview.

All GWAS employed identical QC procedures and a shared mixed-model framework. For the continuous EduYears phenotype, we used fastGWA-LMM (GCTA; --fastGWA-mlm) [61], which implements a linear mixed model (LMM) with a sparse genetic relationship matrix (GRM) to account for relatedness [62]. For all binary milestone phenotypes, including the transition-specific, cumulative, and contrast definitions, we used fastGWA-GLMM [63] (--fastGWA-mlm-binary), the generalized LMM implementation designed for binary outcomes and case-control ascertainment. All association models adjusted for sex, birth year, genotyping batch, and the first ten ancestry principal components (PC1-PC10), with relatedness modeled through the sparse GRM.

The model specification was:


$$\begin{array}{c} \text{Phenotype }\sim \text{ SNP }+\text{ Sex }+\text{ Birth Year }+\;{\text{PC}}_{\text{1}-\text{10}}\;+\text{GenoBatch}+\;\left(\text{1}|\text{GRM}\right) \end{array}$$

where phenotypes were either continuous (EduYears) or binary (milestone completion). Standard QC excluded SNPs with call rate <0.95, MAF < 0.01, imputation INFO <0.80, or Hardy–Weinberg equilibrium p < 1 × 10^−6^. Genome-wide significance was set at two-sided p < 5 × 10^−8^ [64].

Post-GWAS annotation used FUMA [31], identifying independent significant SNPs at r^2^ = 0.6 and lead SNPs at r^2^ = 0.1, and merging LD blocks separated by ≤250 kb, using the UK Biobank 10k European panel as the LD reference. Calibration was assessed with LD score regression (LDSC) on HapMap3 SNPs using 1000G-EUR LD scores. Q-Q and Manhattan plots for all analyses are shown in Figs A–X in S1 Appendix. Numerical values for observed-scale SNP‑heritability (LDSC) and QC metrics (λGC, LDSC intercept, and attenuation ratio) for each GWAS are provided in Table A in S2 Appendix; for cumulative milestones, the table also reports liability-scale LDSC estimates for comparison with twin-based heritability.

A complete list of genome-wide significant lead variants and their clumped genomic loci across the transition-specific, cumulative, contrast, and continuous analyses, together with FUMA-based annotation and strict overlap against reference loci from EA4 and Intelligence (Savage et al., 2018), is provided in Table B in S2 Appendix.

Finally, we used the resulting .fastgwa summary statistics in cross‑trait LDSC for external GWAS comparisons, while BOLT‑REML used individual‑level data for within‑cohort milestone correlations (see subsequent section).

#### Genetic correlations (MoBa).

We estimated pairwise r_g_ to quantify genetic overlap within MoBa, among educational milestones, and between MoBa milestone GWASs and external GWASs of EA and intelligence.

For within-MoBa r_g_, we fit a multivariate BOLT‑REML model [65] jointly to the four cumulative milestone phenotypes. This choice is driven by model identifiability. Transition outcomes are defined on different at‑risk subsets (e.g., the PhD transition is defined only among MSc+). In the complete-case sample required by BOLT-REML, earlier transition outcomes are constant (always 1) among individuals eligible for the latest included transition and therefore have zero variance, leading to non‑convergence. Cumulative outcomes are defined for the full cohort, so all four phenotypes retain non‑zero variance and can be analyzed jointly. Consistent with this rationale, effective sample sizes (see Table 1) differ only modestly between cumulative and transition definitions, indicating that the transition non‑convergence is not explained by lack of power.

For external GWAS comparisons, we used cross‑trait LDSC [66]. Under both transition-specific and cumulative definitions, we benchmarked MoBa EduYears and the milestone GWASs against EduYears (EA4 [24]) and the primary intelligence reference (Savage et al., 2018 [26]). In supplementary analyses, we additionally estimated r_g_ for the transition-specific milestones against the intelligence sensitivity reference (Lam et al., 2021 [27]), CogSub and NonCogSub [28], and EduFields PC1 and PC2 [29]. To formally evaluate whether transition-specific r_g_ differed across milestones, we conducted block-jackknife pairwise comparisons of the r_g_ estimates within each external reference trait, restricted to the transition-specific analyses, and applied within-trait FDR correction. Compact significance matrices and the full pairwise results are provided in the supplement.

LDSC separates polygenic overlap from confounding using genome-wide LD patterns. BOLT-REML is appropriate for overlapping phenotypes measured on the same individuals when all vary in the joint sample (cumulative outcomes), whereas cross-trait LDSC is appropriate for between-cohort comparisons from summary statistics, including the transition-specific outcomes. Together they provide complementary estimates of genetic sharing across milestones and their links to broader constructs of EA and cognition.

#### MiXeR overlap decomposition (MoBa).

As a supplementary overlap-decomposition analysis, we applied MiXeR [30] to the transition-specific GWASs and selected reference traits. MiXeR complements LDSC by decomposing overlap into an overall genetic correlation (r_g_), the fraction of inferred polygenicity shared between two traits, the correlation of effect directions within the shared component (rho_beta), and Dice overlap of the inferred causal-variant sets. Dice overlap quantifies the degree of overlap between the inferred causal-variant sets of two traits, scaled relative to their total sizes. We first fit univariate models for each transition and reference trait and then fit bivariate models for milestone-reference pairs against EA4, MoBa EduYears, the primary intelligence reference (Savage et al., 2018 [26]), the sensitivity intelligence reference (Lam et al., 2021 [27]), CogSub and NonCogSub [28], and EduFields [29] PC1 and PC2. Because stability was assessed from the univariate MiXeR fits, we emphasize the stable HS → BSc+ transition and the MoBa EduYears benchmark as the most interpretable MiXeR results, whereas overlap decompositions for Comp→HS + , BSc → MSc + , and MSc → PhD are interpreted more cautiously and presented as supplementary evidence rather than primary results.

#### SNP-based heritability (MoBa).

We estimated common‑variant heritability (h^2^_SNP_) in MoBa using two complementary estimators. (i) BOLT‑REML (individual‑level): We fitted a multivariate LMM to the four cumulative milestone phenotypes using QC’d autosomal SNPs (minor‑allele frequency > 0.01, imputation INFO > 0.80, Hardy–Weinberg *p* ≥10^-6^, call rate > 0.95), adjusting for sex, birth year, genotyping batch, and PC1–PC10, with genetic relatedness modeled implicitly from the SNP genotypes [65]. (ii) LDSC (summary‑level): We applied LDSC to fastGWA summary statistics [61] using 1000G EUR HapMap3 LD scores and report LDSC intercepts/attenuation ratios as calibration checks [33].

Because outcome definitions differ in identifiability and scale, cumulative and transition phenotypes were handled separately. For cumulative milestones (HS + , BSc + , MSc + , PhD), we report h^2^_SNP_ from both estimators: LDSC on the cumulative‑phenotype GWAS and the heritability estimates from the joint BOLT‑REML model. Both procedures return observed‑scale heritabilities, which we converted to the liability scale to enable direct comparison with twin‑based heritability (h^2^_twin_). The conversion follows the classical liability‑threshold model [67,68] using the mapping of Lee et al. [34] as implemented in bigsnpr [69]. We denote the MoBa sample case fraction by P. For each cumulative milestone, we used the corresponding NTR cumulative milestone prevalence as K, matching the prevalence scale of the twin ACE estimates. P and K values are reported in Table 1 using the MoBa and NTR cumulative rows, respectively.

For transition‑specific milestones (Comp→HS + , HS → BSc + , BSc → MSc + , MSc → PhD), we estimated h^2^_SNP_ with LDSC and retained the resulting estimates on the observed scale rather than transforming them to the liability scale. A liability-scale transform at these transitions would produce conditional quantities specific to the at-risk subpopulation and would therefore not be directly comparable to the population-level liability estimates reported for cumulative milestones. We could not estimate genetic correlations among transition phenotypes using individual-level BOLT-REML because, in the complete-case sample for the included transitions, earlier transition outcomes are constant among those eligible for the latest included transition, yielding zero variance and non-convergence (see Genetic Correlations).

To contextualize precision for binary traits, we report effective sample size *N*_eff_ in Table 1, computed as *N*_eff_ = 4/(1/*N*_cases_+1/*N*_controls_) = 4*NP*(1 − *P*), a standard proxy for power [60]. *N*_eff_ is used for interpretation only and does not enter the liability‑scale conversion.

Using both BOLT‑REML and LDSC for cumulative outcomes provides triangulation: REML is statistically efficient but sensitive to covariate control for structure, whereas LDSC is robust to such confounding via the intercept, albeit typically less efficient. Agreement between the two therefore increases confidence that the reported values reflect common‑variant contributions to the cumulative milestones.

#### Polygenic indices (MoBa).

In MoBa, we constructed genome-wide PGIs using PRS-CS [32] and summary statistics from EA4, excluding the 23andMe cohort because those data are not publicly available [24]. PRS‑CS applies a Bayesian continuous‑shrinkage prior and accounts for linkage disequilibrium through an external LD reference panel, which permits genome‑wide weighting without *p*-value thresholding. After allele alignment and standard QC in the target data, individual-level PGIs were computed and standardized to mean 0 and variance 1 within MoBa.

We evaluated the relation between the EA4 PGI and educational milestones in two complementary ways and under both transition-specific and cumulative definitions. First, we compared mean PGI levels between outcome groups. For transition-specific outcomes, this compared individuals who advanced from the relevant at-risk set with those who did not. For cumulative outcomes, this compared individuals who had attained the milestone or higher with those who had not. Second, we quantified predictive accuracy in multivariable probit models that regressed each binary milestone phenotype on the standardized PGI, adjusting for sex, birth year, and the first ten PCs.

For predictive accuracy we report two metrics that intentionally live on different, interpretable scales. Tjur’s coefficient of discrimination [70] is reported on the observed scale as the difference in mean predicted probabilities between cases and controls; uncertainty is summarized with nonparametric bootstrap 95% confidence intervals obtained by refitting the models on resampled data (1000 iterations).

For cumulative outcomes only, we also report a liability‑scale PGI metric that captures the variance of liability explained by the PGI, obtained from the probit slope using a standardized PGI that was first residualized on the same covariates used in the prediction model; this follows the mapping described by Lee et al. [34], also with bootstrapped (1,000 resamples) 95% confidence intervals.

We do not convert Tjur’s R^2^ to the liability scale because such a transformation is not standard and is not directly comparable to the liability‑variance metric. For transition‑specific outcomes, we keep PGI metrics on the observed scale and pair them with observed‑scale SNP‑based heritability estimates to maintain internal consistency within the conditional framework.

Together, the mean‑difference analyses and the two prediction metrics address related but distinct questions: the former captures between‑group stratification by milestone, while the latter assesses individual‑level discrimination. We interpret these PGI results alongside SNP-based and twin-based heritability estimates to contextualize the explanatory power of the EA-based PGIs.

In supplementary analyses, we extended the transition-specific PGI framework to six additional external summary-statistic sources: intelligence (Savage et al., 2018 [26]), intelligence (Lam et al., 2021 [27]), CogSub and NonCogSub [28], and EduFields PC1 and PC2 [29]. For each trait, PGI scores were standardized within MoBa and analyzed only under the transition-specific milestone framework. We summarized mean PGI differences between those who advanced and those who did not advance at each transition using group means with 95% confidence intervals derived from standard errors. We quantified predictive performance on the observed scale using Tjur’s coefficient of discrimination from probit models, with 95% confidence intervals obtained from 500 bootstrap iterations. We additionally estimated logistic-regression odds ratios with 95% confidence intervals for each transition. Logistic-regression odds ratios used the same covariate adjustment as the probit models. These supplementary PGI results are reported in Figs AL–AN in S1 Appendix and Table N in S2 Appendix.

#### Twin-based heritability (NTR).

We analyzed the NTR sample using a classical twin design [71,72] within a liability-threshold framework, which is appropriate for the binary milestone outcomes. This approach decomposes the variance in liability into additive genetic (A), shared environmental (C), and unique environmental (E) components (the ACE model).

We implemented these models in Stan [73], a platform for Bayesian statistical modeling, to obtain posterior distributions for the ACE parameters, from which we derived point estimates and 95% credible intervals. This Bayesian approach provides more robust uncertainty quantification than traditional maximum likelihood methods, particularly important given the varying prevalences of our educational milestones.

Due to insufficient statistical power and potential model instability for analyzing specific transitions within the NTR sample, our twin analyses were restricted to the cumulative definition of educational milestones. The transition-specific approach would require analyzing twins discordant for consecutive educational levels (e.g., one twin stopping at high school while the other continues to bachelor’s), which yields very small cell sizes given the correlation of EA within twin pairs.

The resulting additive genetic heritability (h^2^_twin_) and the C and E components are all reported on the unobserved liability scale. The A component estimates broader biometric additive genetic variance than common-SNP methods and may include additive effects of genetic variation not well captured by current common-variant arrays, such as rare or structural variants. Under standard twin-model assumptions, however, non-additive genetic effects or assumption violations may also contribute to the A component. Comparing h^2^_twin_ with h^2^_SNP_ therefore provides an estimate of the twin–SNP heritability gap at each educational milestone, rather than a direct attribution of the gap to any specific genetic source. Full details on the model specification, choice of priors, and MCMC settings are provided in Note B in S1 Appendix.

## Supporting information

S1 Appendix Supplementary figures and notes. Contains Figs A–AS and Notes A–D. Note A describes composition and measurement limitations in the EA4 comparator GWAS; Note B provides details and Stan code for the Bayesian twin ACE models; Note C provides frequently asked questions and interpretive clarifications; and Note D provides the illustrative sequential-selection simulation and code. The figures include GWAS Manhattan and QQ plots (Figs A–X), cumulative external-benchmark correlations (Fig Y), ACE decomposition (Fig Z), supplementary external-trait LDSC analyses (Figs AA–AF), MiXeR visual summaries (Figs AG–AK), supplementary PGI figures (Figs AL–AN), descriptive cohort distributions (Figs AO–AP), the NUS2000 educational classification mapping (Fig AQ), and the illustrative sequential-selection simulation (Figs AR–AS).
(DOCX)

S2 Appendix Supplementary tables and numerical source data. Contains Tables A–R, including GWAS QC metrics, lead-variant and FUMA annotation, ACE estimates, supplementary genetic-correlation results, MiXeR summaries, PGI summary statistics, BOLT-REML cumulative genetic correlations and heritability estimates, benchmark genetic-correlation estimates, and PGI source data.
(XLSX)
