# Supplementary material for: Beyond years of schooling: Genetic associations across educational milestones in two Norwegian cohorts
Source: PLoS Genet. 2026 Sep 17;22(9):e1012310. doi: 10.1371/journal.pgen.1012310 (PMC13596786; doi:10.1371/journal.pgen.1012310)
Supplement: Supplementary file 1 [file pgen.1012310.s003.pdf]

Summary of the research

The authors apply a milestone-based framework to the genetic basis for educational attainment, using GWAS-based analyses to investigate the genetic liability for both cumulative and stage-specific elements of education. They focus on heritability, genetic correlations and PGI prediction and use both summary-level and individual-level methods. One key finding is that many elements of the common genetic liability for EA follow an inverse-U pattern, being most predictive of outcomes at the transition to higher education and being less so at the start and end of the education period, where less population variation exists.

Strengths and weaknesses

On strengths, this paper is an effective piece of social science genetics work, bridging GWA studies that use continuous measures to maximise statistical power and the sociological literature which is concerned with the granular milestones which are of great social importance to the individual. Figure 1 is particularly effective in formalising assumptions and frameworks that are typically implicit in nature. The authors also successfully demonstrate robustness of their findings to choice of estimator, such as by estimating heritability using different methods with distinct assumptions, which increases confidence in their findings. Their central finding of qualitative and quantitative heterogeneity using a milestone-based framework will inform how EA PGIs and heritability estimates should (and should not) be interpreted, which is valuable given that education genetics is particularly liable to misinterpretation both within and without the genetics community.

On weaknesses, a key limitation is that the analyses remain correlational and descriptive. While the milestone framework is informative, the paper offers limited insight into underlying pathways or mechanisms. This is a limitation which is common to GWAS-based analysis and education genetics work. Furthermore, one of the main conclusions on heterogeneity at the top of the educational distribution relies on estimates with wide confidence intervals, which warrants more epistemic caution in interpretation.

Overall, this is a strong and conceptually important paper that will be of broad interest to behavioral geneticists and sociogenomic researchers. With revisions, the paper would make a durable contribution to how educational attainment is conceptualised and analysed in genetic research.

Minor issues

First, as with much research of this kind, the paper does not provide pathway-based, causal or biological insights, but presents a largely correlational and association-based genetic framework. While useful for enriching and complementing long-standing empirical work that conceptualises EA as a continuous measure, little is done to provide explanations for the patterns observed.

One relatively straightforward way to address this, particularly given the ongoing challenge of identifying biological or mechanistic genetic explanations for educational outcomes, involves work that has already been identified in the *future research* section (p.15). The authors could estimate genetic correlations with phenotypes beyond EA4 and IQ3, which, while still not being a causal analysis, would provide a richer indication of the genetic variance that is common and unique to educational milestones. Traits that could be considered include personality, mental health, or other cognitive measures, which are all known to be important for educational outcomes. A particularly interesting option would be the novel GWAS of educational fields (Cheesman et al. 2025). Generally, the phenotypes investigated as genetic correlates of education in the cognitive/non-cognitive GWAS of EA would also be a good starting point (Demange et al. 2021).

Second, some of the conclusions rely on estimates with wide confidence intervals, especially for the MSc → PhD milestone. While this is understandable given the smaller sample at the top of the distribution, this limitation ought to be acknowledged more throughout the text, as it does weaken the

claim about heterogeneity. For example, the following sentence could be qualified: “MSc→PhD marked the sharpest decline… indicating reduced alignment with the genetic influences captured by EA4 and IQ3, though with wider confidence intervals at this transition” (p.6).

Third, the exploration of measurement limits and base-rate constraints contains unclear phrasing which could be benefitted by improving the explanations of concepts. For example, the sentence “We next consider mechanisms that could generate these patterns, contrasting measurement limits in external comparators with selection coupled with base‑rate constraints in the at‑risk sets (case fractions far from 0.5 that reduce observed‑scale variance and predictive discrimination), and we argue that the within‑MoBa shapes are most parsimoniously explained by the latter.” (p.13) was particularly difficult to understand. A suggestion would be to either explain “external comparators with selection” and “base rate constraints” further in this paragraph, or to simplify and cut this paragraph entirely, in favour of the explanations which come in the following paragraphs. In comparison, the explanation of selection in the at-risk sets (p.14) is relatively clear.

Other

I will be available to review a revised version of this manuscript.

References

Demange, P.A., Malanchini, M., Mallard, T.T. et al. Investigating the genetic architecture of noncognitive skills using GWAS-by-subtraction. Nat Genet 53, 35–44 (2021). https://doi.org/10.1038/s41588-020-00754-2

Cheesman, R., Anapaz, V., van Alten, S. et al. Genetic associations with educational fields. Nat Genet 57, 2997–3006 (2025). https://doi.org/10.1038/s41588-025-02391-z
