# Supplementary material for: Beyond years of schooling: Genetic associations across educational milestones in two Norwegian cohorts
Source: PLoS Genet. 2026 Sep 17;22(9):e1012310. doi: 10.1371/journal.pgen.1012310 (PMC13596786; doi:10.1371/journal.pgen.1012310)
Supplement: Supplementary file 2 [file pgen.1012310.s004.pdf]

Manuscript summary

This manuscript examines the genetic architecture of educational attainment by reconceptualizing education not as a continuous measure of “years of schooling,” but as a sequence of discrete educational milestones (high school, bachelor’s, master’s, PhD). Using two large Norwegian cohorts (MoBa and the Norwegian Twin Registry), the authors apply complementary transition-specific and cumulative genetic models, combining GWAS, SNP-heritability (LDSC/BOLT-REML), polygenic indices, and twin models to test whether genetic influences are stable across stages of educational progression.

The central claim is that genetic influences on education are dynamic rather than uniform across the educational trajectory. The authors report both qualitative heterogeneity (the composition of genetic influences changes across stages) and quantitative heterogeneity (the magnitude of genetic influence varies by stage). In transition-specific analyses, genetic correlations with large external GWAS of educational attainment (EA4) and intelligence (IQ3) are high for early transitions (e.g., high school and entry into higher education), but decline substantially at postgraduate stages, especially for the MSc→PhD transition. In parallel, SNP-heritability and polygenic score predictability follow a clear inverse-U pattern, peaking at the transition into higher education and declining at the endpoints of the distribution.

In cumulative analyses, the authors show that genetic overlap between milestones decreases with increasing educational distance, while twin-based heritability declines monotonically from high school to PhD. The gap between twin-based and SNP-based heritability is largest at near-universal milestones (high school completion) and narrows at higher degrees, suggesting that the proportion of heritable variance captured by common SNPs is not constant across educational stages. Together, these findings are interpreted as evidence for a dynamic, stage-dependent genetic architecture of education that is obscured by conventional “years of schooling” phenotypes.

Minor Comments

1. Socioeconomic factors

The manuscript would benefit from a more explicit discussion and justification for the decision not to include socioeconomic status (SES)–related factors as covariates. Even if these variables are intentionally treated as part of the educational phenotype rather than confounders, clarifying this choice would help readers interpret the results.

2. Robustness to rare endpoint definitions

The authors may consider adding a small set of sensitivity analyses (e.g., combining MSc and PhD into a single postgraduate category) to demonstrate that the main patterns are robust to how rare educational endpoints are defined.

The authors report a sharp decline in genetic correlations between the MSc→PhD transition and EA4/IQ3. However, Table 1 also shows substantially reduced sample sizes for MSc and PhD

transitions. It would be helpful if the Discussion more explicitly addressed the extent to which reduced statistical power may contribute to attenuated genetic correlations at these stages.

3. Interpretation of transition and cumulative models together

Visually presenting results from the transition-specific and cumulative models side by side (for example, using aligned panels or a summary figure) would help readers more clearly understand how the two modelling frameworks complement each other and how their results should be interpreted jointly and separately.

4. Clarity of expression, use of examples, and annotation

Several sections would benefit from clearer and more direct language, particularly when explaining the authors' conceptual motivations. Providing concrete examples, especially in the context of Figure 4, would make the arguments more accessible.

Figure 5 is currently difficult to interpret due to the overlap of markers and lines. Replotting this figure with clearer visual separation (e.g., fewer overlapping elements or alternative aesthetics) would improve readability.

In Figure 7, the meaning of the red and black outline boxes is not clearly explained in the text or caption. Additional clarification would help readers interpret this figure correctly.

5. Linking results more directly to conceptual claims

The manuscript presents compelling conceptual models regarding stage-specific genetic influences on educational attainment and the limitations of treating education as a continuous variable. However, these models are not always explicitly tied back to the empirical results. The Introduction and Discussion would benefit from a clearer explanation of how the presented findings support (or qualify) these conceptual claims.

6. Placement of phenotype description

The first subsection of the Results primarily describes phenotype construction and GWAS definitions. This material may be more appropriate for the Introduction or Methods section, as it functions largely as background and justification rather than as results.

7. GWAS method for binary phenotypes

A methodological concern is the use of fastGWA-LMM for GWAS with binary (0/1) phenotypes, given that this method was originally designed for quantitative traits. The GCTA documentation recommends fastGWA-GLMM for binary outcomes. Although the authors provide a rationale for their choice, additional sensitivity analyses comparing results across methods would strengthen confidence that this decision does not affect the robustness of the conclusions.

8. Broader contextualisation of genetic correlations

The Discussion could be strengthened by comparing results not only to EA4 and IQ3, but also to a broader set of cognitive or cognitive-adjacent GWAS (e.g., general cognitive ability g, non-cognitive skills derived from GWAS-by-subtraction). This would help move beyond reporting correlations and predictability toward deeper discussion of potential mechanisms underlying the observed stage-specific patterns.
