# Supplementary material for: Beyond years of schooling: Genetic associations across educational milestones in two Norwegian cohorts
Source: PLoS Genet. 2026 Sep 17;22(9):e1012310. doi: 10.1371/journal.pgen.1012310 (PMC13596786; doi:10.1371/journal.pgen.1012310)
Supplement: Supplementary file 3 [file pgen.1012310.s001.docx]

S1 Appendix. Supplementary figures and notes

**Supplementary figures (A–AS)	4**

Transition-specific GWAS results and QC (Figs A–H)	4

Fig A. Manhattan plot, milestone transition Comp→HS+	4

Fig B. QQ plot, milestone transition Comp→HS+	4

Fig C. Manhattan plot, milestone transition HS→BSc+	5

Fig D. QQ plot, milestone transition HS→BSc+	5

Fig E. Manhattan plot, milestone transition BSc→MSc+	6

Fig F. QQ plot, milestone transition BSc→MSc+	6

Fig G. Manhattan plot, milestone transition MSc→PhD	7

Fig H. QQ plot, milestone transition MSc→PhD	7

Cumulative GWAS results and QC (Figs I–P)	8

Fig I. Manhattan plot, cumulative milestone HS+	8

Fig J. QQ plot, cumulative milestone HS+	8

Fig K. Manhattan plot, cumulative milestone BSc+	9

Fig L. QQ plot, cumulative milestone BSc+	9

Fig M. Manhattan plot, cumulative milestone MSc+	10

Fig N. QQ plot, cumulative milestone MSc+	10

Fig O. Manhattan plot, cumulative milestone PhD	11

Fig P. QQ plot, cumulative milestone PhD	11

Contrast GWAS results and QC (Figs Q–V)	12

Fig Q. Manhattan plot, contrast (non-cumulative) BSc vs. HS	12

Fig R. QQ plot, contrast (non-cumulative) BSc vs. HS	12

Fig S. Manhattan plot, contrast (non-cumulative) MSc vs. HS	13

Fig T. QQ plot, contrast (non-cumulative) MSc vs. HS	13

Fig U. Manhattan plot, contrast (non-cumulative) PhD vs. HS	14

Fig V. QQ plot, contrast (non-cumulative) PhD vs. HS	14

EduYears GWAS results and QC (Figs W–X)	15

Fig W. Manhattan plot, EduYears	15

Fig X. QQ plot, EduYears	15

Additional analyses (Figs Y–AN)	16

Fig Y. Genetic correlations between cumulative milestones and EA4 and Intelligence (Savage et al., 2018).	16

Fig Z. ACE decomposition for cumulative milestones - see Note B	17

Fig AA. Transition-specific rg trajectories for EduYears (EA4) and Intelligence (Lam et al., 2021).	18

Fig AB. Compact pairwise significance matrix for EduYears (EA4) and Intelligence (Lam et al., 2021).	19

Fig AC. Transition-specific rg trajectories for CogSub and NonCogSub.	20

Fig AD. Compact pairwise significance matrix for CogSub and NonCogSub.	21

Fig AE. Transition-specific rg trajectories for EduFields PC1 (Technical vs. Social) and EduFields PC2 (Practical vs. Abstract).	22

Fig AF. Compact pairwise significance matrix for EduFields PC1 (Technical vs. Social) and EduFields PC2 (Practical vs. Abstract).	23

Fig AG. MiXeR transition-versus-reference overlap heatmap.	24

Fig AH. MiXeR univariate fit-stability overview.	25

Fig AI. MiXeR shared-versus-unique decomposition for stable analyses.	26

Fig AJ. Schematic MiXeR overlap decompositions for stable analyses.	27

Fig AK. MiXeR rg-versus-shared-fraction scatter for stable analyses.	28

Fig AL. Increment in PGI discrimination across milestone transitions.	29

Fig AM. Mean PGI contrast by educational milestone transition.	30

Fig AN. Odds-ratio trajectories for external PGIs.	31

Cohort descriptive distributions (Figs AO–AP)	32

Fig AO. Educational attainment distribution in the MoBa cohort.	32

Fig AP. Educational attainment distribution in the NTR cohort.	33

NUS2000 educational classification mapping (Fig AQ)	34

Fig AQ. Mapping the Norwegian Standard Classification of Education (NUS2000) to analytical categories.	34

Simulation of sequential selection (Figs AR–AS) - see Note D	35

Fig AR. Illustrative simulation setup and transition-specific thresholding.	35

Fig AS. Consequences of sequential selection under a constant simulated PGI coefficient.	36

Tables and numerical source data (provided separately in S2 Appendix).	37

**Notes A–D	37**

Note A.	37

Composition and Measurement Limitations in the EA4 Comparator GWAS	37

Note B.	38

Methodological Details for the Bayesian Twin Model	38

1. Model Specification	38

2. Hierarchical Structure: Sex- and Cohort-Specific Thresholds	39

3. Choice of Priors	39

4. MCMC Settings and Implementation	40

5. Stan Model Code	40

Note C.	43

Frequently Asked Questions (FAQ)	43

1. Background	43

1.1 What did this study investigate?	43

1.2 Why does it matter whether education is measured as years or milestones?	43

1.3 What data and methods were used?	43

2. Key findings	44

2.1 What did you find?	44

2.2 Does this mean genetics matter more for some educational stages than others?	44

2.3 Why is the genetic signal strongest at the transition into higher education?	45

2.4 What about the twin results?	45

2.5 How confident are you in the findings for postgraduate degrees?	45

3. What the results do NOT mean	46

3.1 Did you find "the genes for" completing university?	46

3.2 Can your results predict whether a specific person will finish a degree?	46

3.3 Do your findings mean that people who do not complete higher education are genetically disadvantaged?	46

3.4 Do your results explain differences between countries, ethnic groups, or social classes?	46

3.5 Does this research imply that educational policy or investment does not matter?	47

3.6 Could someone use these results to select students or make admissions decisions?	47

3.7 Does the weaker genetic signal at postgraduate levels mean that genetics is unimportant for completing a PhD?	47

3.8 Do your findings show that educational success is meritocratic or that the most educated individuals are genetically "better"?	47

4. Broader context	48

4.1 How does this study relate to earlier genetic studies of education?	48

4.2 What are the main limitations of this study?	48

4.3 What would you like future research to address?	48

Note D.	49

Illustrative simulation of sequential selection and PGI attenuation	49

1 Purpose	49

2 Simulation design	49

3 R code	50

3.1 Setup	50

3.2 Simulate PGI	51

3.3 Simulate stage-specific latent liabilities	52

3.4 Define thresholds and cumulative milestone indicators	53

3.5 Visualize the transition-specific liability-threshold mechanism	54

3.6 Summarize transition-specific PGI associations	56

3.7 Plot transition-specific mean PGI contrasts	58

3.8 Plot PGI odds-ratio trajectory	60

3.9 Plot PGI variance among at-risk individuals	61

3.10 Plot transition-specific PGI prediction	62

3.11 Plot transition-specific PGI distributions by advancement status	63

4 Interpretation	66

# **Supplementary figures (A–AS)**

## Transition-specific GWAS results and QC (Figs A–H)

### Fig A. Manhattan plot, milestone transition Comp→HS+

### Fig B. QQ plot, milestone transition Comp→HS+

### Fig C. Manhattan plot, milestone transition HS→BSc+

### Fig D. QQ plot, milestone transition HS→BSc+

### Fig E. Manhattan plot, milestone transition BSc→MSc+

### Fig F. QQ plot, milestone transition BSc→MSc+

### Fig G. Manhattan plot, milestone transition MSc→PhD

### Fig H. QQ plot, milestone transition MSc→PhD

## Cumulative GWAS results and QC (Figs I–P)

### Fig I. Manhattan plot, cumulative milestone HS+

### Fig J. QQ plot, cumulative milestone HS+

### Fig K. Manhattan plot, cumulative milestone BSc+

### Fig L. QQ plot, cumulative milestone BSc+

### Fig M. Manhattan plot, cumulative milestone MSc+

### Fig N. QQ plot, cumulative milestone MSc+

### Fig O. Manhattan plot, cumulative milestone PhD

### Fig P. QQ plot, cumulative milestone PhD

## Contrast GWAS results and QC (Figs Q–V)

### Fig Q. Manhattan plot, contrast (non-cumulative) BSc vs. HS

### Fig R. QQ plot, contrast (non-cumulative) BSc vs. HS

### Fig S. Manhattan plot, contrast (non-cumulative) MSc vs. HS

### Fig T. QQ plot, contrast (non-cumulative) MSc vs. HS

### Fig U. Manhattan plot, contrast (non-cumulative) PhD vs. HS

### Fig V. QQ plot, contrast (non-cumulative) PhD vs. HS

## EduYears GWAS results and QC (Figs W–X)

### Fig W. Manhattan plot, EduYears

### Fig X. QQ plot, EduYears

## Additional analyses (Figs Y–AN)

### Fig Y. Genetic correlations between cumulative milestones and EA4 and Intelligence (Savage et al., 2018).

*Note. Horizontal dashed lines with shaded 95% CI ribbons represent benchmark correlations from the MoBa EduYears GWAS, showing its genetic correlation with EA4 and Intelligence (Savage et al., 2018). The cumulative milestone estimates and benchmark values underlying this figure are reported in Table R in S2 Appendix.*

### Fig Z. ACE decomposition for cumulative milestones - see Note B

*Note. See Note B for Methodological Details for the Bayesian Twin Model. The numerical estimates underlying this figure are reported in Table C in S2 Appendix.*

### Fig AA. Transition-specific r_g_ trajectories for EduYears (EA4) and Intelligence (Lam et al., 2021).

*Note. Points and error bars show rg estimates (95% CIs) for each transition-specific milestone GWAS against the external EduYears (EA4) and Intelligence (Lam et al., 2021) reference GWASs. Horizontal dashed lines with shaded 95% CI ribbons represent benchmark correlations from the MoBa EduYears GWAS. The transition-specific estimates underlying this figure are reported in Table D in S2 Appendix, and the MoBa EduYears benchmark values are reported in Table R in S2 Appendix.*

### Fig AB. Compact pairwise significance matrix for EduYears (EA4) and Intelligence (Lam et al., 2021).

*Note. Symbols summarize within-trait FDR-corrected pairwise block-jackknife comparisons across transitions. The full pairwise test results are reported in Table E in S2 Appendix, and the symbolized matrix entries are reported in Table F in S2 Appendix.*

### Fig AC. Transition-specific r_g_ trajectories for CogSub and NonCogSub.

*Note. Horizontal dashed lines with shaded 95% CI ribbons represent benchmark correlations from the MoBa EduYears GWAS. The transition-specific estimates underlying this figure are reported in Table G in S2 Appendix, and the MoBa EduYears benchmark values are reported in Table R in S2 Appendix.*

### Fig AD. Compact pairwise significance matrix for CogSub and NonCogSub.

*Note. The full pairwise block-jackknife test results are reported in Table H in S2 Appendix, and the symbolized matrix entries are reported in Table I in S2 Appendix.*

### Fig AE. Transition-specific rg trajectories for EduFields PC1 (Technical vs. Social) and EduFields PC2 (Practical vs. Abstract).

*Note. Horizontal dashed lines with shaded 95% CI ribbons represent benchmark correlations from the MoBa EduYears GWAS. The transition-specific estimates underlying this figure are reported in Table G in S2 Appendix, and the MoBa EduYears benchmark values are reported in Table R in S2 Appendix.*

### Fig AF. Compact pairwise significance matrix for EduFields PC1 (Technical vs. Social) and EduFields PC2 (Practical vs. Abstract).

*Note. The full pairwise block-jackknife test results are reported in Table H in S2 Appendix, and the symbolized matrix entries are reported in Table I in S2 Appendix.*

### Fig AG. MiXeR transition-versus-reference overlap heatmap.

*Note. The underlying MiXeR summary metrics are reported in Table J in S2 Appendix.*

### Fig AH. MiXeR univariate fit-stability overview.

*Note. The underlying fit-stability classifications and summary statistics are reported in Table L in S2 Appendix.*

### Fig AI. MiXeR shared-versus-unique decomposition for stable analyses.

*Note. The underlying shared-versus-unique decomposition values are reported in Table M in S2 Appendix.*

### Fig AJ. Schematic MiXeR overlap decompositions for stable analyses.

*Note. The corresponding numerical decomposition values are reported in Table M in S2 Appendix.*

### Fig AK. MiXeR rg-versus-shared-fraction scatter for stable analyses.

*Note. The underlying MiXeR summary metrics are reported in Table J in S2 Appendix.*

### Fig AL. Increment in PGI discrimination across milestone transitions.

*Note. The underlying PGI summary statistics are reported in Table N in S2 Appendix.*

### Fig AM. Mean PGI contrast by educational milestone transition.

*Note. For EduFields PC1 and PC2, the sign of the association reflects the orientation of the field-propensity axis (for example, technical versus social or practical versus abstract), rather than more versus less educational success per se. The underlying PGI summary statistics are reported in Table N in S2 Appendix.*

### Fig AN. Odds-ratio trajectories for external PGIs.

*Note. For EduFields PC1 and PC2, the sign of the association reflects the orientation of the field-propensity axis (for example, technical versus social or practical versus abstract), rather than more versus less educational success per se. The underlying PGI summary statistics are reported in Table N in S2 Appendix.*

## Cohort descriptive distributions (Figs AO–AP)

### Fig AO. Educational attainment distribution in the MoBa cohort.

**Fig AO | EA across birth cohorts in the MoBa sample.** Distribution of EA in the Norwegian Mother, Father and Child Cohort Study (MoBa) sample (N = 120,527) stratified by sex and 5-year birth cohorts.

**a**, Absolute number of individuals in each highest-attainment category.

**b**, Proportion of individuals within each cohort in each highest-attainment category.

### Fig AP. Educational attainment distribution in the NTR cohort.

**Fig AP | EA across birth cohorts in the NTR sample.** Distribution of educational attainment in the Norwegian Twin Registry (NTR) sample (N = 8,910) stratified by sex and 5-year birth cohorts.

**a**, Absolute number of individuals in each highest-attainment category.

**b**, Proportion of individuals within each cohort in each highest-attainment category.

## NUS2000 educational classification mapping (Fig AQ)

### Fig AQ. Mapping the Norwegian Standard Classification of Education (NUS2000) to analytical categories.

**Fig AQ | Mapping the Norwegian Standard Classification of Education (NUS2000) to analytical categories.** The table details the official Norwegian Standard Classification of Education (NUS2000) levels. We mapped these levels to the five primary analytical education categories used in this study: Compulsory Education (Comp), High School (HS), Bachelor's (BSc), Master's (MSc), and Doctorate (PhD). The final column lists the conventional EduYears value assigned to each NUS2000 level. Educational levels 0 (pre-school) and 9 (unspecified) were excluded from all analyses. Adapted from Barrabés & Østli, 2016.

## Simulation of sequential selection (Figs AR–AS) - see Note D

### Fig AR. Illustrative simulation setup and transition-specific thresholding.

**Fig AR | Illustrative simulation setup and transition-specific thresholding.**

A, Simulated standardized PGI distribution. B, Stage-specific latent liabilities L1–L4 generated using the same simulated PGI coefficient at each transition. C, Transition-specific liability-threshold selection among individuals at risk for each transition. Dashed vertical lines indicate transition thresholds; shaded regions indicate individuals who did and did not advance. See Note D for full simulation details and code.

### Fig AS. Consequences of sequential selection under a constant simulated PGI coefficient.

**Fig AS | Consequences of sequential selection under a constant simulated PGI coefficient.**

A, Mean PGI among simulated advancers and non-advancers at each transition. B, Logistic-regression odds ratio per 1-SD PGI across transitions. C, Standard deviation of PGI among individuals at risk for each transition. D, Tjur’s pseudo-R2 for transition-specific PGI prediction. E, PGI distributions among advancers and non-advancers within each transition-specific at-risk group. Although the simulated PGI coefficient is held constant across transitions, sequential selection increases mean PGI in later at-risk groups and reduces PGI variance among individuals at risk. This range restriction, together with endpoint case-control imbalance, attenuates transition-specific discrimination. See Note D for full simulation details and code.

## Tables and numerical source data (provided separately in S2 Appendix).

S2 Appendix contains Tables A–R, including GWAS QC metrics, lead-variant and FUMA annotation, ACE estimates, supplementary rg results, MiXeR summaries, PGI summary statistics, BOLT-REML cumulative genetic correlations and heritability estimates, benchmark rg estimates, and PGI source data.

# **Notes A–D**

## Note A.

### Composition and Measurement Limitations in the EA4 Comparator GWAS

Our analyses of external genetic correlations (r_g_) with educational attainment (EA) and the performance of the EA4-derived polygenic index (PGI) rely on summary statistics from the EA4 meta-analysis. The interpretation of our findings, particularly the attenuated correlations at postgraduate milestones, requires a clear understanding of the composition and measurement properties of the specific EA4 subset available for these comparisons.

Due to data access restrictions, our analyses necessarily excluded summary statistics from the 23andMe cohort, which constitutes approximately 75% of the total EA4 sample size. The remaining, publicly available EA4 subset (N ≈ 765,283) has a distinct composition. The UK Biobank cohort is the largest single contributor, representing approximately 58% of this subset (N ≈ 441,121). Within the UK Biobank, educational attainment is measured with a single binary survey item that distinguishes only whether a participant obtained any college or university degree (Okbay et al., 2022; see their Supplementary Note, Section 1).

The remaining 42% of the subset comprises 69 different cohorts (total N ≈ 324,162) with more varied and often more granular measurement approaches (Lee et al., 2018; see their Supplementary Tables 16, 17, and 22). However, the harmonization of these diverse measures for the EA4 meta-analysis relied on the International Standard Classification of Education (ISCED) 1997 framework (UNESCO, 2006). A key feature of this framework is that it collapses bachelor's and master's degrees into a single category (ISCED level 5), which is then mapped to a single value of 19 years of education in the EduYears metric.

These measurement limitations have direct implications for our findings. Our study, using Norwegian registry data, distinguishes between bachelor’s, master’s, and doctoral degrees. In the public EA4 subset, the UK Biobank contribution uses a binary degree indicator, while the remaining cohorts are harmonized using ISCED-1997 coding that does not distinguish bachelor’s from master’s attainment. Consequently, association signal relevant to master’s-level attainment is combined with signal relevant to bachelor’s-level attainment. This may attenuate the genetic correlation with our BSc→MSc+ transition because the EA4 summary statistics are not designed to isolate this specific progression step. Similarly, although doctoral attainment is represented in some contributing cohorts, the predominance of the binary degree measure in the largest cohort may weaken external signal specific to the highest educational transitions.

## Note B.

### Methodological Details for the Bayesian Twin Model

This note provides detailed information on the specification, priors, and implementation of the Bayesian hierarchical twin model used to estimate heritability for the four educational milestones.

#### **1. Model Specification**

We implemented a liability-threshold model to analyze the binary educational attainment outcomes for same-sex monozygotic (MZ) and dizygotic (DZ) twin pairs. The model assumes that an underlying continuous liability determines the binary outcome (i.e., whether an individual achieves a given educational milestone). An individual *i’*s liability (*L_i_*) is decomposed into additive genetic (*A*), shared environmental (*C*), and unique environmental (*E*) components:

*L_i_ = A_i_ + C_i_ + E_i_*

The total variance of the liability is standardized to 1, such that:

 *Var(A) + Var(C) + Var(E) = h^2^ + c^2^ + e^2^ = 1.*

The parameters h^2^, c^2^, and e^2^ represent the proportions of variance explained by *A*, *C*, and *E*, respectively.

For a given twin pair, the model leverages the genetic relatedness of MZ twins (who share ~100% of their segregating genes) and DZ twins (who share ~50% on average). The shared component of liability for an MZ pair is $\sqrt{h^{2}+c^{2}}$, while for a DZ pair it is $\sqrt{0.5h^{2}+c^{2}}$. The unique (residual) standard deviation for an individual in an MZ pair is $\sqrt{e^{2}}$, and for an individual in a DZ pair it is $\sqrt{e^{2} +0.5h^{2}}$ .

#### **2. Hierarchical Structure: Sex- and Cohort-Specific Thresholds**

To account for secular trends and sex differences in educational attainment across birth cohorts, we modeled the liability threshold as a hierarchical parameter that varied by both sex and cohort. The model estimates separate thresholds for males and females within each birth cohort.

This was implemented as a random walk, where the threshold for a given sex in a specific cohort is determined by a global mean for that sex plus a cumulative deviation specific to that cohort. This flexible approach allows the prevalence of achieving an educational milestone to change non-linearly over time for males and females independently, without imposing a specific functional form on the trend.

#### **3. Choice of Priors**

We used weakly informative priors to regularize the model and ensure stable estimates, while allowing the data to drive the posterior distributions.

- Variance Components (*ACE*): A Dirichlet prior was placed on the simplex of the three variance components:

  *ACE ~ Dirichlet* (1,1,1)

  This is a uniform prior over the space of possible proportions for h^2^, c^2^, and e^2^.
- Global Thresholds (*threshold_global*): The global mean thresholds for males and females were given a standard normal prior:

  *Threshold_global ~ Normal*(0,1)
- Threshold Variation (*threshold_sigma*): The standard deviation of the annual change in thresholds for each sex was given a half-normal prior, weakly centered around zero:

  *Threshold_sigma ~ Normal*(0, 0.2)
- **Standardized Parameters**: All non-centered standardized parameters (*mz_std, dz_std, threshold_std*) were given standard normal priors, *N*(0,1), a standard practice in hierarchical Bayesian models.

#### **4. MCMC Settings and Implementation**

The model was written in Stan, a probabilistic programming language, and posterior distributions were estimated using the No-U-Turn Sampler (NUTS), an efficient variant of Hamiltonian Monte Carlo. The analysis was implemented in R using the *rstan* package (version 2.32.6, interfacing with Stan version 2.32.2).

For each of the four educational milestones (HS+, BSc+, MSc+, and PhD), we ran a separate model.

- For each milestone model, HS+, BSc+, MSc+, and PhD, we ran 8 parallel Markov chains for 6,000 iterations each, with the first 2,000 iterations discarded as warmup. This resulted in 32,000 post-warmup samples for estimating the posterior distributions.

Convergence of the chains was assessed by ensuring the Gelman-Rubin statistic $\hat{R}$ was below 1.01 for all parameters, observing no divergent transitions after warmup, and by visual inspection of the trace plots to check for good mixing and stationarity.

#### **5. Stan Model Code**

functions {

real log_prob_bin(int twin_cat, real twinpair_mu, real threshold, real residual_sd){

real temp_sum = 0;

if (twin_cat == 0){

temp_sum = normal_lcdf(threshold | twinpair_mu, residual_sd);

} else {

temp_sum = log1m_exp(normal_lcdf(threshold | twinpair_mu, residual_sd));

}

return temp_sum;

}

}

data {

int<lower=1> mz_twinpairs_n;

int<lower=1> dz_twinpairs_n;

int<lower=1> T; // number of unique cohorts

int<lower=1, upper=T> mz_cohort[mz_twinpairs_n]; // Cohort index for each mz twin pair

int<lower=1, upper=T> dz_cohort[dz_twinpairs_n]; // Cohort index for each dz twin pair

int<lower=0, upper=1> mz_y[mz_twinpairs_n, 2]; // Outcome for mz twin pairs

int<lower=0, upper=1> dz_y[dz_twinpairs_n, 2]; // Outcome for dz twin pairs

int<lower=0, upper=1> sex_mz[mz_twinpairs_n]; // Sex for mz twin pairs

int<lower=0, upper=1> sex_dz[dz_twinpairs_n]; // Sex for dz twin pairs

}

parameters {

// Heritability parameters

simplex[3] ACE;

// Shared components

vector[mz_twinpairs_n] mz_std;

vector[dz_twinpairs_n] dz_std;

// Educational thresholds - male and female separately

vector[2] threshold_global; // Global threshold

vector<lower = 0>[2] threshold_sigma; // Global threshold - SD of annual change

matrix[T-1, 2] threshold_std; // cohort specific annual CHANGE in threshold

}

transformed parameters {

real mz_shared_sd;

real dz_shared_sd;

real mz_unique_sd;

real dz_unique_sd;

vector[mz_twinpairs_n] mz_re;

vector[dz_twinpairs_n] dz_re;

vector[T] threshold_male;

vector[T] threshold_female;

// Calculate the shared and unique standard deviations

mz_shared_sd = sqrt((ACE[1] + ACE[2]));

dz_shared_sd = sqrt((0.5 * ACE[1] + ACE[2]));

mz_unique_sd = sqrt(ACE[3]);

dz_unique_sd = sqrt(ACE[3] + 0.5 * ACE[1]);

// Random effects for each twin pair

mz_re = mz_shared_sd * mz_std;

dz_re = dz_shared_sd * dz_std;

// Calculate the thresholds for each cohort and sex

{

vector[T] temp_vec = cumulative_sum(append_row(0,threshold_std[,1]));

threshold_male = threshold_global[1] + threshold_sigma[1] * (temp_vec - mean(temp_vec));

temp_vec = cumulative_sum(append_row(0,threshold_std[,2]));

threshold_female = threshold_global[2] + threshold_sigma[2] * (temp_vec - mean(temp_vec));

}

}

model {

// Priors

ACE ~ dirichlet(rep_vector(1, 3));

mz_std ~ std_normal();

dz_std ~ std_normal();

threshold_global ~ normal(0, 1);

threshold_sigma ~ normal(0, 0.2);

to_vector(threshold_std) ~ std_normal();

// Likelihood for MZ twins

for (n in 1:mz_twinpairs_n) {

int t = mz_cohort[n];

real threshold = (sex_mz[n] == 0) ? threshold_male[t] : threshold_female[t]; // Use sex_mz for MZ twins

target += log_prob_bin(mz_y[n, 1], mz_re[n], threshold, mz_unique_sd);

target += log_prob_bin(mz_y[n, 2], mz_re[n], threshold, mz_unique_sd);

}

// Likelihood for DZ twins

for (n in 1:dz_twinpairs_n) {

int t = dz_cohort[n];

real threshold = (sex_dz[n] == 0) ? threshold_male[t] : threshold_female[t]; // Use sex_dz for DZ twins

target += log_prob_bin(dz_y[n, 1], dz_re[n], threshold, dz_unique_sd);

target += log_prob_bin(dz_y[n, 2], dz_re[n], threshold, dz_unique_sd);

}

}

## Note C.

### Frequently Asked Questions (FAQ)

#### 1. Background

##### 1.1 What did this study investigate?

We investigated whether genetic association patterns with education look the same across the educational sequence, or whether they depend on how educational attainment is defined. Most genetic studies of education treat attainment as a single years-based measure. We instead analyzed discrete milestones: completing high school, earning a bachelor’s degree, completing a master’s degree, and obtaining a doctorate. We used two complementary approaches. In the transition-specific approach, we estimated genetic associations with progression to the next milestone among people who had already reached the previous one. In the cumulative approach, we estimated genetic associations with attaining a given milestone or higher in the full cohort.

##### 1.2 Why does it matter whether education is measured as years or milestones?

In practice, educational progress is not a smooth accumulation of years. It involves clearing discrete hurdles, each with different demands, different selection pressures, and different populations of people who are "at risk" of advancing. For example, nearly everyone in Norway completes high school, but very few obtain a doctorate. A years-based approach treats moving from 12 to 13 years of schooling as equivalent to moving from 19 to 20, which may not reflect reality. By analyzing milestones, we can ask whether genetic association patterns are similar across the educational sequence, and how they change when analyses condition on prior attainment.

##### 1.3 What data and methods were used?

We analyzed data from two Norwegian cohorts. The first was the Norwegian Mother, Father and Child Cohort Study (MoBa), which included over 120,000 genotyped adults with registry-linked information on their highest educational credential. The second was the Norwegian Twin Registry (NTR), which included nearly 9,000 twins. For the MoBa cohort, we conducted genome-wide association studies (GWAS) for each milestone, estimated genetic correlations with large international GWAS of educational attainment (EA4) and intelligence, and tested how well existing genetic scores predicted each milestone transition. Within MoBa, some analyses focused on progression to the next stage among people who had already reached the previous one, whereas others compared people who had reached a given milestone with everyone below that threshold in the full cohort. For the NTR cohort, we used classical twin models to estimate the total genetic contribution to each milestone. These genotype-based and twin-based approaches are complementary: the genotype-based methods capture the effects of common genetic variants (those carried by many people in the population), while the twin-based methods reflect a broader set of genetic influences, including those not yet individually identified.

#### 2. Key findings

##### 2.1 What did you find?

Our main finding is that observed genetic association patterns with education are not uniform across milestone definitions. Several specific patterns emerged:

- **Common genetic variant effects peaked at the transition into higher education.**
  In the step-by-step analyses, which compare who advances among those eligible, using genotype-based methods, we found that the genetic signal, including heritability and overlap with international studies of educational attainment and intelligence, was strongest at the transition from high school to higher education.
- **The predictive power of existing genetic scores followed the same pattern.** The widely used polygenic score for educational attainment (from the EA4 study) was most useful for distinguishing who enters higher education and least useful at the endpoints (high school completion and doctoral completion).
- **Twin-based estimates pointed to a different pattern.** Using the cumulative approach, which compares everyone who reached a milestone against everyone who did not, twin analyses suggested that the broader overall genetic contribution to education was largest for completing high school and declined at higher milestones.
- **The gap between twin-based and genotype-based estimates was largest at the high school milestone.** Under the same cumulative approach, common genetic variants, which are what current genetic scores are based on, missed a larger share of the total genetic influence at this near-universal milestone than at later ones.
- **Genetic overlap between milestones declined with increasing distance.** Using the cumulative approach, the genetic factors associated with completing high school overlapped substantially with those for a bachelor's degree but less so with those for a doctorate.

##### 2.2 Does this mean genetics matter more for some educational stages than others?

Not necessarily in the way it might seem. The strength of the genetic signal depends partly on the kinds of groups being compared at each stage. In the step-by-step analyses, each comparison is made only among people who have already cleared all earlier milestones. At the high school endpoint, nearly everyone succeeds, so the small group that does not complete high school is unusual and may be influenced by factors, including genetic factors, that are not well captured by current common-variant methods. At the doctoral endpoint, by contrast, the people being compared have already been heavily filtered by prior educational success. At both ends of the sequence, the remaining differences between the groups are therefore harder for genotype-based methods to detect. This helps explain why genetic signal and predictive accuracy are weaker at the endpoints. Our findings should not be interpreted as showing that genetics is inherently less important for doctoral completion. Note D illustrates this point with a simulation showing that transition-specific PGI discrimination can attenuate even when the simulated PGI coefficient is held constant across transitions. The transition-specific attenuation is therefore compatible with sequential selection and does not, by itself, show that underlying genetic effects differ across stages. Such differences remain possible, but are not needed to explain the observed pattern. In the cumulative analyses, which compare each milestone with everyone below it in the full population, this progressive selection is weaker, and that likely contributes to the flatter pattern across milestones.

##### 2.3 Why is the genetic signal strongest at the transition into higher education?

This is likely driven in large part by the statistical properties of the comparison. The transition into higher education has the largest and most balanced at-risk population, with roughly equal numbers of high school graduates advancing and not advancing. This balance maximizes the power of our methods to detect genetic influences. At the endpoints, by contrast, the groups are highly unbalanced: nearly everyone completes high school (compressing variation at the bottom), and very few obtain a doctorate (compressing variation at the top, where everyone has already been selected on education-related traits). The peak signal at the bachelor's transition therefore reflects favorable statistical conditions rather than necessarily indicating that common genetic variants are biologically more relevant at this particular stage. It is also the transition most closely aligned with what existing large-scale GWAS of education and intelligence were designed to capture: broad differences in attainment around the median of the distribution. In the cumulative analyses, which do not condition on prior stages, the pattern is flatter because the comparison groups are less affected by this progressive selection.

##### 2.4 What about the twin results?

The twin analyses provide a different perspective. While the genotype-based methods capture only common genetic variants, the twin design reflects the full range of inherited influences (though it rests on assumptions that may not be perfectly met). The twin results showed that the overall genetic contribution was highest for completing high school and declined for later milestones. The divergence between twin and genotype-based estimates was also largest at the high school milestone, suggesting that common genetic variants alone do not capture the full picture there. However, the twin estimates were imprecise, particularly for the rarer milestones, and the confidence intervals overlapped across milestones. We therefore interpret the twin results as indicative of a trend rather than definitive evidence of large differences.

##### 2.5 How confident are you in the findings for postgraduate degrees?

Less confident than for the earlier milestones. The number of individuals at postgraduate transitions, especially for the doctoral stage, is much smaller. This means our estimates are less precise and our confidence intervals are wider. While the general pattern of attenuation at postgraduate levels is consistent across multiple methods and measures, the specific numerical estimates for the MSc-to-PhD transition should be treated with appropriate caution. We highlight this uncertainty throughout the paper.

#### 3. What the results do NOT mean

##### 3.1 Did you find "the genes for" completing university?

No. We did not identify specific genes that determine whether someone completes a particular degree. The genetic variants identified in our genome-wide analyses each have extremely small effects. No single variant meaningfully predicts educational outcomes. What we found are statistical patterns across the genome that, when aggregated, are associated with educational milestones at the group level.

##### 3.2 Can your results predict whether a specific person will finish a degree?

No. Our polygenic score analyses show that even the most predictive genetic scores explain only a small fraction of the differences between people (roughly 5% of the variation at the most informative transition, and much less at other transitions). The vast majority of the differences in educational outcomes between individuals are not captured by genetics. Genetic scores are statistical tools useful for research, not prediction tools for individuals.

##### 3.3 Do your findings mean that people who do not complete higher education are genetically disadvantaged?

No. Educational outcomes are shaped by a complex interplay of individual characteristics, family circumstances, institutional structures, economic conditions, and chance. Genetic associations are probabilistic and population-level; they do not define any individual's potential. Many people with high polygenic scores do not pursue higher education, and many people with lower scores do. Importantly, the genetic variants we study do not have fixed or inevitable effects. Their associations with educational outcomes depend on the social and institutional context in which people live.

##### 3.4 Do your results explain differences between countries, ethnic groups, or social classes?

No. Our study was conducted entirely within Norway and in a population of primarily European genetic ancestry. The genetic associations we find are specific to this population and its educational system. Genetic findings from one population cannot be meaningfully extrapolated to explain differences between populations, countries, or social groups. Differences in educational outcomes between groups are overwhelmingly shaped by social, economic, historical, and institutional factors.

##### 3.5 Does this research imply that educational policy or investment does not matter?

No. The existence of genetic influences on educational outcomes does not diminish the importance of educational environments, policies, or investments. In fact, genetic influences on education are partly mediated through environmental pathways (for example, parents with certain genetic variants may tend to have higher incomes, which in turn affects their children's educational opportunities). Effective educational policies can change outcomes for everyone, regardless of their genetic background. Norway's near-universal high school completion rate is itself a testament to the power of institutional and policy factors.

##### 3.6 Could someone use these results to select students or make admissions decisions?

Our results should not be used for this purpose. The predictive accuracy of genetic scores for individual outcomes is far too low and the ethical objections are far too serious for any such application. Our study is designed to improve scientific understanding of how genetic and environmental factors interact across the educational sequence, not to provide tools for individual classification.

##### 3.7 Does the weaker genetic signal at postgraduate levels mean that genetics is unimportant for completing a PhD?

No. A weaker observed genetic signal does not mean that genetic factors are unimportant for doctoral attainment. In the transition-specific MSc→PhD analysis, everyone being compared has already attained at least a master’s degree, so the analysis is conducted within a highly selected group with less remaining variation in education-related characteristics. In the cumulative PhD analysis, doctoral attainment is rare, producing a highly imbalanced comparison and lower statistical precision. These features can weaken observed genetic signal and predictive discrimination without implying weaker underlying genetic effects at the doctoral stage. As discussed in Section 2.2, our simulation illustrates this point directly for transition-specific PGI discrimination.

##### 3.8 Do your findings show that educational success is meritocratic or that the most educated individuals are genetically "better"?

No. Our findings do not support this interpretation. The genetic variants associated with educational attainment are not measures of merit, ability, or worth. They are statistical associations that reflect a complex mix of cognitive traits, non-cognitive traits, family environments, and social structures. Higher polygenic scores do not mean someone is more deserving of educational success, and lower scores do not mean someone is less capable. Educational systems are shaped by institutional design, funding, admissions policies, and social norms, all of which affect who advances regardless of genotype. Our results describe population-level statistical patterns, not individual merit.

#### 4. Broader context

##### 4.1 How does this study relate to earlier genetic studies of education?

Previous large-scale GWAS of education (EA1 through EA4) have treated educational attainment as a continuous measure of years of schooling. These studies have been enormously productive, identifying thousands of genetic variants and producing polygenic scores that are widely used in research. Our study builds on this work, but asks a different question: does the years-based approach obscure meaningful heterogeneity across educational stages? Our findings suggest that it does. Genetic association patterns with education differ across milestone definitions in ways that are not visible when education is collapsed into a single EduYears measure. This does not invalidate the years-based approach but rather shows what additional information can be gained by preserving the milestone structure.

##### 4.2 What are the main limitations of this study?

Several important limitations should be kept in mind:

- **Population specificity.** Our findings reflect the Norwegian educational system and a primarily European-ancestry population. They may not generalize to other countries or populations.
- **Statistical precision at rare milestones.** Estimates for postgraduate transitions, especially the doctorate, are imprecise due to small sample sizes at these stages.
- **Correlation, not causation.** Our analyses are associational. We cannot determine whether the genetic variants we identify causally influence educational outcomes or whether they are associated through indirect pathways (for example, through effects on parental behavior or family environment).
- **Twin model assumptions.** The twin-based heritability estimates rest on classical assumptions (for example, that shared environments affect monozygotic and dizygotic twins equally) that may not be perfectly met and could differ across milestones.
- **External reference limitations.** Our comparisons with the EA4 GWAS inherit that study's measurement limitations, including its reliance on a coarse binary degree measure in a large portion of its sample.
- **Selection and non-identification.** Observed differences across milestones can be shaped by selection, case composition, phenotype definition, and measurement, so the analyses do not by themselves identify why genetic associations vary across milestones.

##### 4.3 What would you like future research to address?

We see several promising directions:

- **Scaling up.** Larger samples, particularly for postgraduate milestones, would allow more precise estimation of genetic influences at the top of the educational distribution and could enable construction of milestone-specific polygenic scores.
- **Broader traits.** Examining genetic correlations with personality, mental health conditions, and neurodevelopmental traits across milestones could help clarify what drives the stage-specific patterns we observe.
- **Cross-country comparisons.** Applying the same milestone framework in countries with different educational structures (for example, varying levels of tuition, admission selectivity, or public funding) would test how general these patterns are.
- **Diverse populations.** Extending this work to multi-ancestry samples is essential for understanding whether the milestone-specific patterns we observe are universal or specific to European-ancestry populations.
- **Family-based designs.** Within-family analyses could test which milestone association patterns remain after accounting for shared family background, assortative mating, dynastic effects, and genetic nurture.

## Note D.

### Illustrative simulation of sequential selection and PGI attenuation

#### 1 Purpose

This note presents an illustrative simulation showing how attenuation in transition-specific PGI discrimination can arise from sequential selection and range restriction, even when the simulated PGI coefficient is held constant across educational transitions. The simulation uses the manuscript notation and includes transition-specific threshold visualizations, mean-contrast and odds-ratio panels, and full annotated R code.

The simulation is intended as an illustrative constant-coefficient scenario. It is not fitted to the empirical MoBa data and is not intended to provide a realistic generative model of educational attainment. Its purpose is to show that a decline in transition-specific PGI discrimination does not, by itself, establish that the underlying PGI association is weaker at later milestones.

The notation follows the manuscript. Educational milestones are denoted as HS+ (at least high school), BSc+ (at least bachelor’s), MSc+ (at least master’s), and PhD. The transition-specific contrasts are Comp→HS+, HS→BSc+, BSc→MSc+, and MSc→PhD.

#### 2 Simulation design

We simulate a standardized PGI for N = 500,000 individuals:

$${PGI}_{i}\sim N(0,1).$$

For each transition, we also simulate a stage-specific non-PGI factor, independent of the PGI:

$$E_{ki}\sim N(0,1), k\in\{1,2,3,4\}.$$

The latent liability for each transition is generated as:

$$L_{ki}=\beta_{g}{PGI}_{i}+\beta_{e}E_{ki}+\varepsilon_{ki},$$

with:

$$\beta_{g}=0.7, \beta_{e}=0.7, \varepsilon_{ki}\sim N(0,1).$$

The key design choice is that $\beta_{g}$ is identical for all four transitions. Thus, the simulated PGI effect on latent liability is held constant across Comp→HS+, HS→BSc+, BSc→MSc+, and MSc→PhD.

Sequential milestone indicators are then generated by applying liability thresholds:

$${HS}_{i}^{+}=1\left( L_{1i}>t_{1} \right),$$

$${BSc}_{i}^{+}=1\left( {HS}_{i}^{+}=1 \wedge L_{2i}>t_{2} \right),$$

$${MSc}_{i}^{+}=1\left( {BSc}_{i}^{+}=1 \wedge L_{3i}>t_{3} \right),$$

$${PhD}_{i}=1\left( {MSc}_{i}^{+}=1 \wedge L_{4i}>t_{4} \right).$$

The thresholds are chosen to create increasingly selective transitions. Because the same PGI contributes to each latent liability, individuals reaching later milestones are increasingly selected on PGI, even though the PGI coefficient is unchanged.

#### 3 R code

##### 3.1 Setup

**set.seed**(123)

**library**(dplyr)
**library**(tidyr)
**library**(ggplot2)

*# Store automatically generated Quarto figures in a predictable folder.*
**dir.create**("figures/simulation_note", recursive = TRUE, showWarnings = FALSE)
knitr**::**opts_chunk**$set**(
 dpi = 300,
 fig.path = "figures/simulation_note/"
)

**theme_set**(
 **theme_minimal**(base_size = 14) **+**
 **theme**(
 plot.title = **element_text**(hjust = 0.5, face = "bold", size = 12),
 plot.subtitle = **element_text**(hjust = 0.5, size = 12),
 plot.title.position = "plot"
 )
)

N <- 500000

##### 3.2 Simulate PGI

*# Genetic predictor, e.g. an EA- or intelligence-related PGI.*
*# It is standardized to have mean 0 and SD 1 in the simulated population.*

PGI <- **rnorm**(N)

p_pgi <- **ggplot**(**data.frame**(PGI = PGI), **aes**(x = PGI)) **+**
 **geom_density**(linewidth = 1.2) **+**
 **labs**(
 title = "Simulated PGI distribution",
 x = "PGI (standardized)",
 y = "Density"
 )

p_pgi

*Simulated standardized PGI distribution.*

##### 3.3 Simulate stage-specific latent liabilities

*# Stage-specific non-PGI factors.*
*# These are independent of PGI and independent across transitions.*
E1 <- **rnorm**(N)
E2 <- **rnorm**(N)
E3 <- **rnorm**(N)
E4 <- **rnorm**(N)

*# PGI and non-PGI effects.*
*# The PGI effect is intentionally held constant at every transition.*
beta_g <- 0.7
beta_e <- 0.7

*# Latent liabilities for the four educational transitions.*
L1 <- beta_g ***** PGI **+** beta_e ***** E1 **+** **rnorm**(N)
L2 <- beta_g ***** PGI **+** beta_e ***** E2 **+** **rnorm**(N)
L3 <- beta_g ***** PGI **+** beta_e ***** E3 **+** **rnorm**(N)
L4 <- beta_g ***** PGI **+** beta_e ***** E4 **+** **rnorm**(N)

liability_plot_data <- **stack**(**data.frame**(L1 = L1, L2 = L2, L3 = L3, L4 = L4))
**names**(liability_plot_data) <- **c**("liability", "stage")

p_liability <- **ggplot**(liability_plot_data, **aes**(x = liability, colour = stage)) **+**
 **geom_density**(linewidth = 1) **+**
 **theme**(
 legend.position = **c**(0.86, 0.60),
 legend.background = **element_rect**(fill = scales**::alpha**("white", 0.75), colour = NA)
 ) **+**
 **labs**(
 title = "Distributions of latent liabilities L1–L4",
 x = "Latent liability",
 y = "Density",
 colour = "Stage"
 )

p_liability

*Distributions of stage-specific latent liabilities L1–L4.*

##### 3.4 Define thresholds and cumulative milestone indicators

*# Thresholds are chosen so that later milestones are increasingly selective.*
*# We use unname() because quantile() returns named values by default.*
t1 <- **unname**(**quantile**(L1, 0.25))
t2 <- **unname**(**quantile**(L2, 0.55))
t3 <- **unname**(**quantile**(L3, 0.75))
t4 <- **unname**(**quantile**(L4, 0.90))

*# Cumulative milestone indicators, matching the notation in the manuscript.*
HS_plus <- L1 **>** t1
BSc_plus <- HS_plus **&** L2 **>** t2
MSc_plus <- BSc_plus **&** L3 **>** t3
PhD	<- MSc_plus **&** L4 **>** t4

*# Analysis data frame.*
dat <- **data.frame**(
 PGI = PGI,
 L1 = L1,
 L2 = L2,
 L3 = L3,
 L4 = L4,
 HS_plus = HS_plus,
 BSc_plus = BSc_plus,
 MSc_plus = MSc_plus,
 PhD = PhD
)

##### 3.5 Visualize the transition-specific liability-threshold mechanism

transition_levels <- **c**("Comp→HS+", "HS→BSc+", "BSc→MSc+", "MSc→PhD")

transition_data <- **bind_rows**(
 **data.frame**(
 liability = dat**$**L1,
 transition = "Comp→HS+",
 threshold = t1
 ),
 **data.frame**(
 liability = dat**$**L2[dat**$**HS_plus],
 transition = "HS→BSc+",
 threshold = t2
 ),
 **data.frame**(
 liability = dat**$**L3[dat**$**BSc_plus],
 transition = "BSc→MSc+",
 threshold = t3
 ),
 **data.frame**(
 liability = dat**$**L4[dat**$**MSc_plus],
 transition = "MSc→PhD",
 threshold = t4
 )
) **%>%**
 **mutate**(transition = **factor**(transition, levels = transition_levels))

*# Estimate one smooth density per transition and then shade the areas below*
*# and above the threshold. This avoids the artificial discontinuity that appears*
*# if separate densities are estimated for advancers and non-advancers.*
density_data <- transition_data **%>%**
 **group_by**(transition) **%>%**
 **group_modify**(**~** {
 d <- **density**(.x**$**liability, n = 512)
 threshold <- **unique**(.x**$**threshold)

 **data.frame**(
 liability = d**$**x,
 density = d**$**y,
 threshold = threshold,
 transition_outcome = **ifelse**(d**$**x **>** threshold, "Advanced", "Did not advance")
 )
 }) **%>%**
 **ungroup**() **%>%**
 **mutate**(
 transition_outcome = **factor**(
 transition_outcome,
 levels = **c**("Did not advance", "Advanced")
 )
 )

threshold_data <- transition_data **%>%**
 **distinct**(transition, threshold)

*# Place each transition label above its dashed threshold line rather than using*
*# centered facet-strip labels. This makes the link between the transition name*
*# and the threshold more explicit.*
threshold_label_data <- density_data **%>%**
 **group_by**(transition) **%>%**
 **summarise**(
 threshold = **unique**(threshold),
 y_label = **max**(density) ***** 1.04,
 .groups = "drop"
 )

p_transition <- **ggplot**(density_data, **aes**(x = liability, y = density)) **+**
 **geom_area**(**aes**(fill = transition_outcome), alpha = 0.45) **+**
 **geom_line**(linewidth = 0.9) **+**
 **geom_vline**(
 data = threshold_data,
 **aes**(xintercept = threshold),
 linetype = "dashed",
 linewidth = 0.9,
 inherit.aes = FALSE
 ) **+**
 **geom_text**(
 data = threshold_label_data,
 **aes**(x = threshold, y = y_label, label = transition),
 inherit.aes = FALSE,
 vjust = 0,
 size = 3.6,
 fontface = "bold"
 ) **+**
 **facet_wrap**(**~** transition, nrow = 1) **+**
 **coord_cartesian**(xlim = **c**(**-**5, 5), clip = "on") **+**
 **scale_y_continuous**(expand = **expansion**(mult = **c**(0, 0.12))) **+**
 **theme**(
 strip.text = **element_blank**(),
 strip.background = **element_blank**(),
 panel.spacing.x = grid**::unit**(1.4, "lines"),
 plot.margin = **margin**(5.5, 10, 14, 5.5),
 legend.position = "bottom",
 legend.direction = "horizontal",
 aspect.ratio = 1
 ) **+**
 **labs**(
 title = "Transition-specific educational selection",
 subtitle = "Each panel includes only individuals at risk for that transition",
 x = "Stage-specific latent liability",
 y = "Density",
 fill = "Transition outcome"
 )

p_transition

*Transition-specific liability-threshold selection. Each panel includes only individuals at risk for that transition.*

##### 3.6 Summarize transition-specific PGI associations

*# Function for transition-specific PGI summaries.*
*# The logistic model is used only to compute fitted probabilities, odds ratios, and Tjur's R2 in the simulation.*
*# The empirical manuscript analyses use covariate-adjusted probit* *models; the qualitative simulation point does not depend on this link function.*

transition_summary <- **function**(data, at_risk, outcome, label) {
 d <- data[data[[at_risk]], ]
 y <- d[[outcome]]

 fit <- **glm**(y **~** PGI, data = d, family = **binomial**(link = "logit"))
 pred <- **fitted**(fit)

 **tibble**(
 transition = label,
 n_at_risk = **nrow**(d),
 n_advance = **sum**(y),
 advance_rate = **mean**(y),
 mean_PGI_at_risk = **mean**(d**$**PGI),
 sd_PGI_at_risk = **sd**(d**$**PGI),
 mean_PGI_advanced = **mean**(d**$**PGI[y **==** TRUE]),
 mean_PGI_not_advanced = **mean**(d**$**PGI[y **==** FALSE]),
 PGI_mean_difference = **mean**(d**$**PGI[y **==** TRUE]) **-** **mean**(d**$**PGI[y **==** FALSE]),
 log_OR_PGI = **unname**(**coef**(fit)["PGI"]),
 SE_log_OR_PGI = **unname**(**summary**(fit)**$**coefficients["PGI", "Std. Error"]),
 OR_PGI = **unname**(**exp**(**coef**(fit)["PGI"])),
 OR_lower = **unname**(**exp**(**coef**(fit)["PGI"] **-** 1.96 ***** **summary**(fit)**$**coefficients["PGI", "Std. Error"])),
 OR_upper = **unname**(**exp**(**coef**(fit)["PGI"] **+** 1.96 ***** **summary**(fit)**$**coefficients["PGI", "Std. Error"])),
 pseudo_R2_Tjur = **mean**(pred[y **==** TRUE]) **-** **mean**(pred[y **==** FALSE])
 )
}

*# Explicit at-risk indicator for the first transition.*
dat**$**All <- TRUE

res <- **bind_rows**(
 **transition_summary**(dat, "All", "HS_plus", "Comp→HS+"),
 **transition_summary**(dat, "HS_plus", "BSc_plus", "HS→BSc+"),
 **transition_summary**(dat, "BSc_plus", "MSc_plus", "BSc→MSc+"),
 **transition_summary**(dat, "MSc_plus", "PhD", "MSc→PhD")
) **%>%**
 **mutate**(transition = **factor**(transition, levels = transition_levels))

res_display <- res **%>%**
 **select**(
 transition,
 n_at_risk,
 n_advance,
 advance_rate,
 mean_PGI_at_risk,
 sd_PGI_at_risk,
 mean_PGI_advanced,
 mean_PGI_not_advanced,
 PGI_mean_difference,
 log_OR_PGI,
 OR_PGI,
 pseudo_R2_Tjur
 )

knitr**::kable**(
 res_display,
 digits = 3,
 caption = "Simulation summary table. Later at-risk groups have higher mean PGI and lower PGI variance, even though the simulated PGI coefficient is identical at every transition."
)

*Simulation summary table. Later at-risk groups have higher mean PGI and lower PGI variance, even though the simulated PGI coefficient is identical at every transition.*

| **Transition** | **N_at_**  **risk** | **N_**  **advance** | **Advance**  **_rate** | **Mean_**  **PGI_**  **at_risk** | **sd_PGI_**  **at_risk** | **Mean_**  **PGI_**  **advanced** | **Mean_**  **PGI_not_**  **advanced** | **PGI_**  **mean_**  **difference** | **Log_**  **OR_PGI** | **OR_PGI** | **Pseudo_2_Tjur** |
| --- | --- | --- | --- | --- | --- | --- | --- | --- | --- | --- | --- |
| **Comp→HS+** | 500000 | 375000 | 0.750 | 0.000 | 1.001 | 0.211 | -0.633 | 0.844 | 0.985 | 2.677 | 0.143 |
| **HS→BSc+** | 375000 | 184272 | 0.491 | 0.211 | 0.941 | 0.573 | -0.139 | 0.712 | 0.940 | 2.561 | 0.145 |
| **BSc→MSc+** | 184272 | 63207 | 0.343 | 0.573 | 0.878 | 0.997 | 0.352 | 0.645 | 0.950 | 2.585 | 0.124 |
| **MSc→PhD** | 63207 | 13062 | 0.207 | 0.997 | 0.826 | 1.487 | 0.869 | 0.617 | 0.989 | 2.688 | 0.098 |

##### 3.7 Plot transition-specific mean PGI contrasts

*# This plot parallels the manuscript's mean PGI contrast figure.*
*# Each transition is summarized among the relevant at-risk group.*

transition_group_summary <- **function**(data, at_risk, outcome, label) {
 d <- data[data[[at_risk]], ]

 d **%>%**
 **mutate**(
 transition = label,
 group = **if_else**(.data[[outcome]], "Advanced", "Did not advance")
 ) **%>%**
 **group_by**(transition, group) **%>%**
 **summarise**(
 n = **n**(),
 mean_PGI = **mean**(PGI),
 se_PGI = **sd**(PGI) **/** **sqrt**(n),
 .groups = "drop"
 )
}

mean_contrast_data <- **bind_rows**(
 **transition_group_summary**(dat, "All", "HS_plus", "Comp→HS+"),
 **transition_group_summary**(dat, "HS_plus", "BSc_plus", "HS→BSc+"),
 **transition_group_summary**(dat, "BSc_plus", "MSc_plus", "BSc→MSc+"),
 **transition_group_summary**(dat, "MSc_plus", "PhD", "MSc→PhD")
) **%>%**
 **mutate**(
 transition = **factor**(transition, levels = transition_levels),
 group = **factor**(group, levels = **c**("Did not advance", "Advanced"))
 )

p_mean_contrast <- **ggplot**(mean_contrast_data, **aes**(x = transition, y = mean_PGI, fill = group)) **+**
 **geom_col**(position = **position_dodge**(width = 0.75), width = 0.65) **+**
 **geom_errorbar**(
 **aes**(ymin = mean_PGI **-** 1.96 ***** se_PGI, ymax = mean_PGI **+** 1.96 ***** se_PGI),
 position = **position_dodge**(width = 0.75),
 width = 0.2
 ) **+**
 **theme**(
 legend.position = **c**(0.20, 0.82),
 legend.background = **element_rect**(fill = scales**::alpha**("white", 0.75), colour = NA),
 legend.key.size = grid**::unit**(0.45, "cm"),
 plot.margin = **margin**(5.5, 12, 5.5, 5.5),
 plot.subtitle = **element_text**(hjust = 0.5, size = 12)
 ) **+**
 **labs**(
 title = "Mean PGI contrast across simulated transitions",
 subtitle = "Both groups become increasingly selected on PGI**\n**at later transitions",
 x = "Educational transition",
 y = "Mean PGI",
 fill = "Transition outcome"
 )

p_mean_contrast

*Mean PGI among simulated advancers and non-advancers at each transition.*

##### 3.8 Plot PGI odds-ratio trajectory

*# The odds ratio is calculated from the transition-specific logistic model used*
*# in the simulation. Here, the OR trajectory remains comparatively stable*
*# while Tjur's R2 attenuates.*

p_or <- **ggplot**(res, **aes**(x = transition, y = OR_PGI, group = 1)) **+**
 **geom_hline**(yintercept = 1, linetype = "dashed") **+**
 **geom_errorbar**(**aes**(ymin = OR_lower, ymax = OR_upper), width = 0.12) **+**
 **geom_point**(size = 3) **+**
 **geom_line**() **+**
 **theme**(
 plot.margin = **margin**(5.5, 10, 5.5, 5.5),
 plot.subtitle = **element_text**(hjust = 0.5, size = 12)
 ) **+**
 **labs**(
 title = "PGI odds ratio remains approximately stable",
 subtitle = "The simulated PGI coefficient is identical**\n**at every transition",
 x = "Educational transition",
 y = "Odds ratio per 1-SD PGI"
 )

p_or

*Simulated PGI odds-ratio trajectory across transitions.*

##### 3.9 Plot PGI variance among at-risk individuals

p_sd <- **ggplot**(res, **aes**(x = transition, y = sd_PGI_at_risk, group = 1)) **+**
 **geom_point**(size = 3) **+**
 **geom_line**() **+**
 **theme**(plot.margin = **margin**(5.5, 10, 5.5, 5.5)) **+**
 **labs**(
 title = "Range restriction:**\n**PGI variance shrinks in later at-risk groups",
 x = "Educational transition",
 y = "SD of PGI among at-risk individuals"
 )

p_sd

*PGI variance among at-risk individuals across transitions.*

##### 3.10 Plot transition-specific PGI prediction

p_tjur <- **ggplot**(res, **aes**(x = transition, y = pseudo_R2_Tjur, group = 1)) **+**
 **geom_point**(size = 3) **+**
 **geom_line**() **+**
 **theme**(
 plot.margin = **margin**(5.5, 10, 5.5, 5.5),
 plot.subtitle = **element_text**(hjust = 0.5, size = 12)
 ) **+**
 **labs**(
 title = "PGI prediction attenuates under sequential selection",
 subtitle = "The simulated PGI coefficient is identical**\n**at every transition",
 x = "Educational transition",
 y = "Tjur pseudo-R2"
 )

p_tjur

*Transition-specific PGI prediction across simulated educational transitions.*

##### 3.11 Plot transition-specific PGI distributions by advancement status

*# This plot parallels the manuscript's PGI distributional-overlap figure.*
*# Each transition is represented in the relevant at-risk group, with separate*
*# PGI distributions for individuals who do and do not advance.*

transition_pgi_distribution <- **function**(data, at_risk, outcome, label) {
 d <- data[data[[at_risk]], ]

 d **%>%**
 **transmute**(
 PGI = PGI,
 transition = label,
 group = **if_else**(.data[[outcome]], "Advanced", "Did not advance")
 )
}

pgi_transition_distribution_data <- **bind_rows**(
 **transition_pgi_distribution**(dat, "All", "HS_plus", "Comp→HS+"),
 **transition_pgi_distribution**(dat, "HS_plus", "BSc_plus", "HS→BSc+"),
 **transition_pgi_distribution**(dat, "BSc_plus", "MSc_plus", "BSc→MSc+"),
 **transition_pgi_distribution**(dat, "MSc_plus", "PhD", "MSc→PhD")
) **%>%**
 **mutate**(
 transition = **factor**(transition, levels = transition_levels),
 group = **factor**(group, levels = **c**("Did not advance", "Advanced"))
 )

p_density_transition <- **ggplot**(
 pgi_transition_distribution_data,
 **aes**(x = PGI, fill = group, colour = group)
) **+**
 **geom_density**(alpha = 0.35, linewidth = 0.75, adjust = 1.05) **+**
 **facet_wrap**(**~** transition, ncol = 1) **+**
 **coord_cartesian**(xlim = **c**(**-**4, 4)) **+**
 **theme**(
 panel.spacing.y = grid**::unit**(0.9, "lines"),
 legend.position = "bottom"
 ) **+**
 **labs**(
 title = "Sequential selection shifts PGI distributions by transition outcome",
 subtitle = "Both advancers and non-advancers are increasingly selected on PGI at later transitions",
 x = "PGI",
 y = "Density",
 fill = "Transition outcome",
 colour = "Transition outcome"
 )

p_density_transition

*PGI distributions among simulated advancers and non-advancers at each transition.*

#### 4 Interpretation

The simulation generates five key patterns. First, the mean PGI among individuals at risk for later transitions increases across the educational sequence. Second, the PGI variance among at-risk individuals decreases. Third, both advancers and non-advancers are increasingly selected on PGI at later transitions, while the transition-specific mean contrast between them remains visible. Fourth, transition-specific PGI discrimination, summarized by Tjur’s $R^{2}$, can attenuate even while the simulated odds-ratio trajectory remains comparatively stable. Fifth, the transition-specific PGI distribution plots show that later at-risk groups are both shifted and narrowed relative to earlier transitions.

In this constant-coefficient scenario, attenuation in transition-specific PGI discrimination is not diagnostic of a weaker underlying PGI association. It can arise because later analyses are estimated within increasingly selected at-risk groups. Related conditioning and case-composition concerns may also affect other transition-specific association statistics, although this simulation directly evaluates PGI discrimination rather than genetic correlations or SNP heritability.

This simulation is directed at the transition-specific framework, where repeated conditioning on prior attainment progressively restricts the at-risk set. Cumulative attained-status contrasts are less exposed to this specific mechanism because they are estimated in the full cohort, but they remain nested threshold phenotypes whose observed-scale estimates can still be shaped by endpoint prevalence, case fractions, and phenotype scaling.

*For clarifications or additional questions, please contact:* *Eirik H. Kvalvik (eirikhkv@uio.no)* *Center for Lifespan Changes in Brain and Cognition, Department of Psychology, University of Oslo*
