# Supplementary material for: Beyond years of schooling: Genetic associations across educational milestones in two Norwegian cohorts
Source: PLoS Genet. 2026 Sep 17;22(9):e1012310. doi: 10.1371/journal.pgen.1012310 (PMC13596786; doi:10.1371/journal.pgen.1012310)
Supplement: Supplementary file 4 [file pgen.1012310.s005.docx]

**Response to Reviewers**

PGENETICS-D-25-01264

*Beyond years of schooling: Shifting genetic influences across educational milestones in two Norwegian cohorts*

Kvalvik, Wang, Walhovd, Lyngstad & Røgeberg

Dear Editors and Reviewers,

We thank the Editors and all four Reviewers for their constructive and thorough evaluation of our manuscript. Their comments have substantially improved the paper. Below we provide a detailed, point-by-point response to each comment. Reviewer comments are shown in shaded boxes, and our responses follow below. All references below refer to the revised manuscript with tracked changes.

**A summary of the major changes:**

In the revised manuscript, we made six main changes.

First, we reframed all transition-specific findings explicitly as genetic associations with progression conditional on prior attainment, with the attenuation at later milestones interpreted in terms of range restriction and selection rather than as evidence that intelligence becomes unimportant (Reviewer #1 Comments 1–3).

Second, we added sensitivity analyses using Lam et al. (2021) intelligence summary statistics and replaced the “IQ3” shorthand with explicit labels throughout (Reviewer #1 Comment 4).

Third, we added formal block-jackknife pairwise tests for differences in genetic correlation across transitions, with within-trait FDR correction (Reviewer #1 Comment 5; new Supplementary Tables S6 & S9, and Supplementary Figs. S28, S30, S32).

Fourth, we extended the supplementary LDSC and PGI analyses to additional external traits, including GWAS-by-subtraction cognitive (CogSub) / non-cognitive (NonCogSub) phenotypes (Demange et al. 2021) and educational-field axes (PC1 & PC2) (Cheesman et al. 2025) (Reviewers #2 and #4; new Supplementary Figs. S29–S32, S38–S40 and Tables S7–S9, S14).

Fifth, we completed MiXeR overlap decomposition analyses (Reviewer #3; new Supplementary Figs. S33–S37 and Tables S10–S13).

Sixth, we clarified that all binary-phenotype GWAS were already conducted with fastGWA-GLMM, added an Author Summary and a detailed lay-audience FAQ (Supplementary Note 3), expanded Supplementary Table S2 with FUMA annotation and EA4/intelligence locus overlap, and added an explicit justification for not conditioning on SES.

**Additional Editor Comments**

**Editor:** *While most reviewers appreciated the overall goal of the study, they also highlighted several issues that should be addressed before the manuscript can be considered further. In particular, I would like to pay attention to Reviewer #1’s comments about collider bias, the lack of formal comparison between the genetic correlations derived, and the need to more carefully discuss the meaning of the findings.*

**Response:** We thank the Editor for this guidance. We have addressed each of these priorities in detail:

**(1) Collider bias and range restriction:** We revised the Results and Discussion to avoid implying that lower h²_SNP_ or r_g_ at later milestones means that intelligence “matters less.” Instead, we now frame all milestone results as genetic associations with progression conditional on prior attainment and explicitly note that later transitions operate in increasingly selected at-risk groups, so attenuation is expected under range restriction. We removed the “two-component architecture” sentence. The new supplementary PGI analyses are consistent with this interpretation: the largest incremental predictive values occurred at HS→BSc+ for NonCogSub (ΔTjur’s R² = 2.10%, 95% CI 1.94–2.27), intelligence (Savage et al., 2018) (2.00%, 1.84–2.18), intelligence (Lam et al., 2021) (1.97%, 1.82–2.14), and CogSub (1.66%, 1.53–1.80), while mean intelligence-related PGIs increased across milestones for both advancers and non-advancers (Supplementary Figs. S38–S39; Table S14). We interpret this pattern as consistent with cognitive ability continuing to matter for reaching higher educational stages, while repeated selection into later at-risk groups reduces the residual variance available to distinguish who progresses further.

**(2) Formal comparison of genetic correlations:** We conducted block-jackknife pairwise tests (200 blocks) across transitions for each reference trait, with within-trait FDR correction. These tests confirm that the attenuation at MSc→PhD is statistically robust, not merely a visual impression. For EduYears (EA4), all three contrasts involving MSc→PhD were FDR-significant at q < 0.001, and HS→BSc+ also differed from BSc→MSc+ at q < 0.05. For intelligence (Lam et al., 2021), the same three MSc→PhD contrasts were FDR-significant at q < 0.01. Results are in Supplementary Fig. S28 and Tables S4–S6. Additional pairwise tests for CogSub, NonCogSub, and EduFields PCs are in Tables S7–S9 and Figs. S30, S32.

**(3) Careful discussion of findings:** We revised the Abstract, Results, and Discussion to use more uncertainty-aware language and to distinguish clearly between strong and suggestive findings. The MSc→PhD estimates are now described as directionally consistent but less precise. The Abstract now concludes that attenuation at later transitions should be interpreted in the context of conditional progression and increasingly selected at-risk groups.

**Reviewer #1**

We thank Reviewer #1 for their careful and constructive critique, which substantially improved the manuscript.

**Comment 1 — Abstract (Textual)**

*The final sentence of the abstract is far too strong. I don’t believe any one has ever argued that genetic influences are uniform genetic influences across time or educational progression. There’s actually a lot of literature detailing how heritability of cognitive ability (which contributes substantially to the heritability of education) changes across the lifespan.*

**Response:** We agree and have softened the Abstract accordingly. The revised final sentence now reads: “These results, obscured by EduYears metrics, indicate a dynamic genetic architecture across educational milestones and suggest that attenuation at later transitions should be interpreted in the context of conditional progression and increasingly selected at-risk groups.”

**Comment 2 — Collider bias and range restriction (Textual reframing)**

*The authors seem very keen to frame their findings in terms of biology rather than an artifact of the methods that they have used… This wording invites a biological interpretation… rather than the more simple explanation of the genetic correlation is attenuated because of range restriction… The authors need to be very clear that these results are likely selection-induced attenuation rather than implying intelligence matters less for attaining a PhD. Similarly, the phrasing of “This contrast points to a two-component architecture…” is also misleading as this too can simply be attributed to restricting the variance as described above.*

**Response:** We agree and have substantially revised the interpretation.

(a) In the Results section describing Figure 2, we now write: “MSc→PhD showed the weakest overlap with both EduYears (r_g_ ≈ 0.38 [0.13–0.64]) and intelligence (r_g_ ≈ 0.28 [0.07–0.50]), but these estimates were also markedly less precise. The substantially smaller effective sample size at MSc→PhD (Neff = 8,185; Table 1) likely contributes to this imprecision and should be considered alongside the attenuation itself.”

(b) In the Discussion we now devote two paragraphs to selection within the at-risk sets as the primary account: “The weaker predictive performance, lower genetic correlations, and reduced observed-scale signal at the highest milestones should therefore not be interpreted as evidence that these traits matter less there. Rather, they are more consistent with those traits having already contributed to selection into the at-risk group, leaving less residual trait-related variance with which to distinguish who advances further.”

(c) The "two-component architecture" sentence has been deleted entirely. We now frame the cumulative r_g_ gradient primarily in terms of selection and range restriction at later milestones, while noting that the pattern remains compatible with substantial shared genetic influences and some genuine differentiation at higher levels.

(d) The new supplementary PGI analyses reinforce this interpretation: mean PGI values for intelligence-related traits are progressively higher at later milestones for both advancers and non-advancers, indicating that these later transitions are drawn from increasingly selected groups (Supplementary Fig. S39; Table S14).

**Comment 3 — Framing of conditional results (Textual reframing)**

*This is related to my prior comment. These results are best described as genetic associations with progression of education, conditional on having passed earlier grades/levels not as the genetics of education level in general.*

**Response:** We agree and have implemented this throughout. Transition-specific results are now consistently described as “genetic associations with educational progression conditional on prior attainment” rather than as the genetics of educational levels in general.

**Comment 4 — Choice of intelligence GWAS (Methodological / New analysis)**

*The authors use the reference of Savage et al for their measure of IQ… the authors refer to this as IQ3. It’s not clear why they have selected this as the name… However, the largest GWAS of intelligence is Lam et al 2021 with 373,617 participants. As such, I don’t really understand why the authors have selected this data set nor why they believe it is the third GWAS on Intelligence.*

**Response:** We have made three changes:

(a) We have replaced the “IQ3” label throughout the manuscript with “intelligence (Savage et al., 2018),” which is a neutral and accurate description.

(b) We retained Savage et al. (2018) as our primary intelligence reference because it indexes cognitive ability without incorporating educational attainment signals via MTAG, which is important given that our study specifically aims to disentangle genetic influences on education from those on intelligence. We have added Lam et al. (2021; N = 373,617) as a sensitivity analysis. The sensitivity figure (Supplementary Fig. S27) shows that the Lam et al. reference yields nearly identical r_g_ trajectories across transitions, with the same early high-overlap pattern and attenuation at MSc→PhD.

(c) Regarding the concern about SES conditioning in the UK Biobank VNR measure: the convergence between the Savage and Lam results in our sensitivity analysis provides reassurance that this issue does not materially affect our conclusions.

**Comment 5 — Formal statistical tests for r_g_ differences (New analysis)**

*Could the authors show if their estimates of the genetic correlations between educational milestones and intelligence and education differ significantly. At present the authors primarily rely on visual inspection and descriptive language… claims of lower rgs or of distinct genetic architectures should be interpreted cautiously.*

**Response:** We conducted formal block-jackknife pairwise tests (200 blocks) across transitions for each reference trait, with within-trait FDR correction. These formal tests confirm that the attenuation centered on MSc→PhD is statistically significant, not merely a visual impression. Key results:

• For EduYears (EA4): all three contrasts involving MSc→PhD were FDR-significant at q < 0.001; HS→BSc+ also differed from BSc→MSc+ at q < 0.05.

• For intelligence (Lam et al., 2021): the three MSc→PhD contrasts were FDR-significant at q < 0.01.

• For CogSub, NonCogSub, and EduFields PCs: additional pairwise tests are provided.

Results are presented in compact significance matrices (Supplementary Figs. S28, S30, S32) and full pairwise tables (Supplementary Tables S4–S9).

**Comment 6 — FAQ for lay audience (New document)**

*The authors make strong claims regarding the role of genetics in education. As is common in such papers, I strongly advise the authors write a detailed FAQ document that details what they found, what it means and importantly what it does not mean, and draft this for a lay audience.*

**Response:** We appreciate this suggestion and have prepared a detailed FAQ document structured around four sections: (1) Background, (2) Key findings, (3) What the results do NOT mean, and (4) Broader context. The FAQ addresses common misinterpretations including genetic determinism, individual prediction, group differences, and policy implications. It is included as Supplementary Note 3 in the Supplementary Information, with a pointer in the Discussion.

**Reviewer #2**

We thank Reviewer #2 for their careful reading and constructive suggestions.

**Comment 1 — Broader genetic correlations (New analysis)**

*The authors could estimate genetic correlations with phenotypes beyond EA4 and IQ3… Traits that could be considered include personality, mental health, or other cognitive measures. A particularly interesting option would be the novel GWAS of educational fields (Cheesman et al. 2025). Generally, the phenotypes investigated in the cognitive/non-cognitive GWAS of EA would also be a good starting point (Demange et al. 2021).*

**Response:** We have expanded our analyses to include both recommended trait sets:

**(a) GWAS-by-subtraction (Demange et al. 2021):** We estimated transition-specific r_g_ with the cognitive (CogSub) and non-cognitive (NonCogSub) components. Both broadly follow the attainment-related trajectory, with the transition into higher education showing the strongest alignment. Results are in Supplementary Figs. S29–S30 and Tables S7–S9.

**(b) Educational fields (Cheesman et al. 2025):** We estimated transition-specific r_g_ with EduFields PC1 (Technical vs. Social) and PC2 (Practical vs. Abstract). These field-propensity axes show a different profile across milestones compared to the attainment-related traits, consistent with them indexing opposing field-choice propensities rather than a unidirectional liability to educational advancement. Results are in Supplementary Figs. S31–S32 and Tables S7–S9.

**(c) Supplementary PGIs:** We additionally computed PGIs for all six traits (Savage, Lam, CogSub, NonCogSub, EduFields PC1, PC2) and evaluated their transition-specific mean differences, Tjur’s R², and odds ratios. The strongest individual-level predictive signal was at HS→BSc+ for NonCogSub, intelligence, and CogSub, while field PCs showed directionally mixed contrasts (Supplementary Figs. S38–S40 and Table S14).

These new analyses are introduced in the Results and discussed in the Discussion paragraphs on qualitative heterogeneity. In this revision we prioritized the conceptually closest extensions to educational attainment and intelligence; broader personality and psychiatric traits remain an important direction for future work and are now noted explicitly in the Discussion.

**Comment 2 — Wide confidence intervals at MSc→PhD (Textual)**

*Some of the conclusions rely on estimates with wide confidence intervals, especially for the MSc → PhD milestone… this limitation ought to be acknowledged more throughout the text, as it does weaken the claim about heterogeneity.*

**Response:** We have added qualifications throughout. In the Results, MSc→PhD estimates are now described as “markedly less precise” with explicit reference to the smaller Neff. In the Discussion, we state: “The decline at the latest transition should nevertheless be interpreted with caution, because that endpoint is estimated with substantially less precision than the earlier milestones”. The Abstract now includes “albeit with wider confidence intervals.”

**Comment 3 — Unclear phrasing on measurement limits (Textual)**

*The sentence “We next consider mechanisms that could generate these patterns, contrasting measurement limits in external comparators with selection coupled with base-rate constraints in the at-risk sets…” was particularly difficult to understand.*

**Response:** We have rewritten this passage. The Discussion now separates the two arguments rather than compressing them into a single sentence: “We next consider two explanations for these patterns. One concerns the external benchmarks themselves: years-based GWAS may blur distinctions that our milestone framework preserves. The other concerns selection within the educational sequence…”

**Reviewer #3**

We thank Reviewer #3 for their positive assessment and helpful suggestions for improvement.

**Figure 1 caption — Norwegian context (Minor)**

*Figure 1 caption for Panel A might need to add that EduYears–achievement correspondence was derived from the Norwegian school system… I also wonder whether the coloured areas for MSc+ and PhD in panel C were supposed to be smaller than the corresponding “Advanced” areas in panel B.*

**Response:** We have added to the Figure 1A caption: “The illustrative correspondence between EduYears values and educational credentials shown here is anchored to the Norwegian educational system and should not be assumed to map identically across countries.” Regarding Panel C, we have added an explanation: “Except for the first milestone, the shaded advanced areas are larger in the transition-specific panels because these prevalences are conditioned on the relevant at-risk subset rather than the full cohort.”

**Observed-scale h²SNP clarification (Minor)**

*“We report observed scale h²SNP estimates…” → might be useful to clarify “We report LDSC observed scale, rather than liability-scale, h²SNP estimates…”*

**Response:** We agree and have made this wording more explicit. The Results now state: “For transition phenotypes, we report LDSC observed-scale rather than liability-scale h²_SNP_ estimates to avoid assumptions about conditional population prevalences; r_g_ estimates are scale-free and therefore unaffected by this choice.”

**Birth cohort and probit regression (Methods clarification)**

*“Using probit regression adjusted for sex, birth cohort, and ten ancestry principal components…” do the authors mean they controlled for birth cohort bins?*

**Response:** We have clarified that birth year was included as a continuous covariate in the GWAS and PGI models. The model specification in the Methods now reads: “Phenotype ~ SNP + Sex + Birth Year + PC1–10 + GenoBatch + (1|GRM).” The NTR twin model uses cohort-specific thresholds via a random walk, as detailed in Supplementary Note 2.

**Figure 4 presentation (Figures)**

*I find Figure 4 a bit misleading as it is not immediately clear the two figures represent exactly the same data, just on a different scale. To me it would be preferable to only report Figure 4b.*

**Response:** We considered this suggestion carefully and decided to retain both panels because they answer complementary questions. Panel a shows that the advancing and non-advancing groups differ in average PGI level, with the mean contrasts labeled directly, whereas panel b shows that the full PGI distributions still overlap substantially despite those mean differences. To make this explicit, we revised the Figure 4 caption: “Panels a and b are complementary: panel a summarizes mean-level stratification, whereas panel b shows the considerable distributional overlap that remains even when group means differ.”

**MiXeR analyses (New analysis)**

*I would be curious to see MiXeR analyses complement LDSC, as we would know whether shared genetic signal is concordant or discordant in effects across transitions.*

**Response:** We have conducted MiXeR analyses for all four transition-specific GWASs against eight reference traits (EA4, MoBa EduYears, Savage 2018, Lam 2021, CogSub, NonCogSub, EduFields PC1 & PC2). MiXeR requires large GWAS samples for reliable parameter identification; Frei et al. (2019) recommend a minimum of N ≈ 50,000. Among the four milestone transitions, HS→BSc+ showed sufficiently consistent univariate MiXeR support for cautious interpretation under the AIC criterion, although under the stricter BIC criterion it was less stable than MoBa EduYears, which served as the more robust overall attainment benchmark. For HS→BSc+, MiXeR indicated high shared overlap with EA4 (shared fraction 0.89, rg = 0.91), MoBa EduYears (0.74, rg = 0.86), NonCogSub (0.73, rg = 0.76), and intelligence (Savage: 0.57, rg = 0.60; Lam: 0.61, rg = 0.60). The most informative contrast was that EA4 showed stronger overlap with HS→BSc+ than with MoBa EduYears overall (MoBa EduYears vs EA4: shared fraction 0.72, rg = 0.85), whereas MoBa EduYears showed equal or greater overlap than HS→BSc+ with intelligence, CogSub, and NonCogSub. We therefore present MiXeR as supplementary architectural evidence, treating HS→BSc+ and MoBa EduYears as the only attainment phenotypes sufficiently interpretable for these external-trait comparisons and retaining caution for the other milestone transitions because their univariate MiXeR identification was weaker. Results are in Supplementary Figs. S33–S37 and Tables S10–S13.

**Assessment of family relationships (Methods clarification)**

*How were relationships assessed, based on parent report or genetics? It would be important to clarify whether monozygotic twins were included in the GWAS analyses and how they were distinguished from duplicate samples at the genotype level.*

**Response:** Relatedness in the MoBa GWAS analyses was assessed genetically rather than by parent report, through the MoBaPsychGen pipeline. Our milestone GWAS analyzed only MoBa parents. In this adult analytic sample, we identified only two parent-parent pairs with relatedness coefficient ≥ 0.8, indicating MZ/near-duplicate-level relatedness. Because duplicate samples were removed in the upstream MoBaPsychGen QC pipeline, these residual very-close pairs are unlikely to materially influence the results. They were retained and modeled through the sparse GRM in the mixed-model GWAS. Given that this concerns only two pairs among 120,527 adults, we do not believe it has any substantial effect on the association estimates. We have revised the Methods to make the genetic assessment of relatedness more explicit.

**Consistent terminology across figures (Figures)**

*“Cases” and “Controls” are named “Advanced” and “Not advanced” in Figure 1. It would be worth using the wording consistently across all figures.*

**Response:** We appreciate this point. The figures use different label pairs because they serve different communicative functions: Figures 1B and 4 describe the transition-specific contrast intuitively as "Advanced" versus "Did not advance," while Figure 1C describes the cumulative contrast as "Attained" versus "Not attained," reflecting the distinction between conditional progression and unconditional status. Figure 7 and Table 1 retain "Cases" and "Controls" because they define the formal GWAS phenotype structure, where this terminology is standard. We have revised the Figure 7 caption to make the mapping between these label sets explicit, so that the correspondence is clear to the reader. We believe this context-appropriate labeling is more informative than a single label pair applied uniformly across frameworks that ask different questions.

**Birth cohort variable explanation (Methods)**

*In the Statistical Analyses part or in the Methods, the authors should explain what the “Birth Cohort” variable represents.*

**Response:** We have clarified that the covariate is “birth year” (continuous) in the GWAS and PGI models. The model specification now reads: “Phenotype ~ SNP + Sex + Birth Year + PC1–10 + GenoBatch + (1|GRM).”

**Genome-wide significant hits (Reporting)**

*I would want to know the lead independent significant hits at least for the GWAS with genome-wide significant results, and whether they align with EA or IQ GWAS hits.*

**Response:** We have extended Supplementary Table S2 to incorporate FUMA-based lead-variant annotation together with strict overlap against reference loci from EA4 and Intelligence (Savage et al., 2018). Focusing on the transition-specific GWASs, the Comp→HS+ analysis contained 2 lead variants, of which 1 overlapped both EA4 and Savage intelligence loci; the HS→BSc+ transition-specific GWAS yielded 12 lead variants, of which 7 overlapped EA4 loci and 5 overlapped Savage intelligence loci, with 5 overlapping both reference architectures; and the BSc→MSc+ GWAS contained 3 lead variants, of which 1 overlapped both EA4 and Savage loci. The MSc→PhD transition-specific GWAS did not yield genome-wide significant lead variants. Supplementary Table S2 also reports the corresponding lead-variant overlaps for the cumulative, contrast, and EduYears analyses. The Methods now describe this: “A complete list of genome-wide significant lead variants and their clumped genomic loci across the transition-specific, cumulative, contrast, and continuous analyses, together with FUMA-based annotation and strict overlap against reference loci from EA4 and Intelligence (Savage et al., 2018), is provided in Supplementary Table S2.”

**Reviewer #4**

We thank Reviewer #4 for their supportive assessment and thoughtful suggestions.

**Comment 1 — Socioeconomic factors (Discussion)**

*The manuscript would benefit from a more explicit discussion and justification for the decision not to include socioeconomic status (SES)–related factors as covariates.*

**Response:** We have added a dedicated Discussion paragraph clarifying why SES-related measures were not included as default covariates in the primary milestone GWASs. Briefly, we do not regard parental SES as a straightforward confounder in this setting, because it may partly reflect genetically influenced parental traits, correlate with offspring genotype through inheritance, and help structure the family environment through which genetic influences on educational progression may operate. Adjusting for it could therefore remove part of the family-mediated pathway linking genotype to educational progression, rather than simply reduce confounding.

**Comment 2 — Robustness to rare endpoint definitions (Sensitivity analysis)**

*The authors may consider adding sensitivity analyses (e.g., combining MSc and PhD into a single postgraduate category)… It would be helpful if the Discussion more explicitly addressed the extent to which reduced statistical power may contribute to attenuated genetic correlations.*

**Response:** We did not add a separate new analysis combining MSc and PhD because that estimand is captured by our existing phenotype definitions: the cumulative MSc+ phenotype includes both MSc and PhD attainment, and the transition-specific BSc→MSc+ milestone treats postgraduate advancement as entry into either an MSc or a PhD. We now note this explicitly in the Methods. We also clarify in the Results and Discussion that attenuation at the final transition should be interpreted cautiously because that endpoint is estimated less precisely; in the Results, we note that the smaller effective sample size at MSc→PhD (Neff = 8,185; Table 1) likely contributes to this imprecision.

**Comment 3 — Interpretation of transition and cumulative models together (Figures)**

*Visually presenting results from the transition-specific and cumulative models side by side would help readers more clearly understand how the two modelling frameworks complement each other.*

**Response:** We considered this, but the two frameworks use different scales (observed vs liability) and a direct visual comparison would also reflect additional assumptions (transition-specific outcomes lack straightforward population prevalences). To better address the point, we now clarify how the two frameworks relate also in Methods and Results sections.

**Comment 4 — Clarity of expression, examples, and annotation (Textual / Figures)**

*(a) Providing concrete examples, especially in the context of Figure 4, would make the arguments more accessible. (b) Figure 5 is currently difficult to interpret… (c) In Figure 7, the meaning of the red and black outline boxes is not clearly explained.*

**Response:** (a) We have revised the conceptual sections throughout for clarity. The Figure 4 caption already included concrete numeric examples (from ≈ 0.58 s.d. at HS+ to ≈ 0.25 s.d. at PhD); in the revision, we tightened the caption wording and added a sentence making the complementary relationship between panels explicit: “Panels a and b are complementary: panel a summarizes mean-level stratification, whereas panel b shows the considerable distributional overlap that remains even when group means differ.”

(b) We have replotted Figure 5 with improved label placement so that pair labels no longer overlap, making the figure substantially easier to read.

(c) The Figure 7 caption now explains: “The black-outlined box marks the EduYears cut point corresponding to compulsory education (Comp), whereas the red-outlined boxes mark the higher cut points used to map EduYears to HS+, BSc+, MSc+, and PhD. These illustrative EduYears-to-milestone correspondences are anchored to the Norwegian educational system.”

**Comment 5 — Linking results to conceptual claims (Discussion)**

*The Introduction and Discussion would benefit from a clearer explanation of how the presented findings support (or qualify) these conceptual claims.*

**Response:** The original Discussion already organized findings around qualitative and quantitative heterogeneity. In the revision, we strengthened this structure in several concrete ways. First, the qualitative-heterogeneity section was expanded from three evidence patterns to four: the new second pattern integrates the supplementary external-trait analyses showing that GWAS-by-subtraction CogSub and NonCogSub follow the attainment-related trajectory while educational-field PCs show a different profile (Supplementary Figs. S29–S32; Tables S7–S9). Second, we removed the “two-component architecture” claim (flagged by Reviewer #1) and replaced it with language acknowledging that the r_g_ gradient is compatible with selection-induced attenuation. Third, we added selection and range-restriction caveats throughout both the qualitative and quantitative sections, including the new Discussion paragraphs on selection within the at-risk sets. Fourth, each empirical finding is now tied more explicitly to the conceptual claim it supports or qualifies.

**Comment 6 — Placement of phenotype description (Structure)**

*The first subsection of the Results primarily describes phenotype construction and GWAS definitions. This material may be more appropriate for the Introduction or Methods section.*

**Response:** We agree that this material should be streamlined. Rather than moving it entirely to the Methods, we condensed the opening Results subsection so that it takes less space while retaining the conceptual Figure 1, which we consider essential for interpreting the results that follow. Many readers proceed directly to the Results without reading the Methods, and the transition-specific versus cumulative distinction is critical for understanding the subsequent findings. The detailed phenotype construction, case/control definitions, Figure 7, and Table 1 remain in the Methods, where they were already placed. The net effect is a more concise Results opening that proceeds more quickly to empirical findings.

**Comment 7 — GWAS method for binary phenotypes (Methodological)**

*A methodological concern is the use of fastGWA-LMM for GWAS with binary (0/1) phenotypes, given that this method was originally designed for quantitative traits. The GCTA documentation recommends fastGWA-GLMM for binary outcomes.*

**Response:** After re-checking our original pipeline, we confirmed that all binary milestone GWAS were already run with fastGWA-GLMM (--fastGWA-mlm-binary), whereas the continuous EduYears phenotype was analyzed with fastGWA-LMM. We have revised the Methods to state this explicitly: “For the continuous EduYears phenotype, we used fastGWA-LMM (GCTA; --fastGWA-mlm). For all binary milestone phenotypes, including the transition-specific, cumulative, and contrast definitions, we used fastGWA-GLMM (--fastGWA-mlm-binary), the generalized linear mixed-model implementation designed for binary outcomes and case-control ascertainment.”

**Comment 8 — Broader contextualisation of genetic correlations (New analysis)**

*The Discussion could be strengthened by comparing results not only to EA4 and IQ3, but also to a broader set of cognitive or cognitive-adjacent GWAS (e.g., non-cognitive skills derived from GWAS-by-subtraction).*

**Response:** As detailed in our response to Reviewer #2 Comment 1, we have expanded our analyses to include GWAS-by-subtraction CogSub and NonCogSub (Demange et al. 2021) and EduFields PCs (Cheesman et al. 2025), with both r_g_ and PGI analyses across all four transitions. We have also added Lam et al. (2021; N = 373,617) as a sensitivity intelligence reference alongside Savage et al. (2018), which provides a broader cognitive ability measure and addresses the reviewer’s suggestion regarding general cognitive ability g. These analyses reveal that the attainment-related cognitive and non-cognitive components broadly follow the same trajectory as EA4 and intelligence, while the field-propensity axes show a distinct, non-monotonic profile. This is discussed in the revised Results and Discussion.

We believe these revisions substantively address the concerns raised by the Editors and Reviewers, and that the manuscript has been substantially strengthened. We look forward to the Editors’ decision.

Sincerely,

Eirik H. Kvalvik (on behalf of all authors)
