# Supplementary material for: Beyond years of schooling: Genetic associations across educational milestones in two Norwegian cohorts
Source: PLoS Genet. 2026 Sep 17;22(9):e1012310. doi: 10.1371/journal.pgen.1012310 (PMC13596786; doi:10.1371/journal.pgen.1012310)
Supplement: Supplementary file 5 [file pgen.1012310.s006.docx]

Manuscript #: PGENETICS-D-25-01264R1

Title: **Beyond years of schooling: Shifting genetic influences across educational milestones in two Norwegian cohorts**

The authors have substantially improved the manuscript and addressed some of my major concerns. However, I encourage restraint in interpreting the observed attenuation across educational milestones as evidence of a substantively distinct genetic architecture, given that many observed patterns remain plausibly attributable to selection and range restriction induced by conditioning on prior educational attainment. I have included at the end of my review a number of simulations that illustrate how, where every genetic predictor has the same statistical effect at every educational transition, selection alone can produce the effects seen by the authors.

Reviewer Comment 1

The final sentence of the abstract is far too strong. I don’t believe any one has ever argued that genetic influences are uniform genetic influences across time or educational progression. There’s actually a lot of literature detailing how heritability of cognitive ability (which contributes substantially to the heritability of education) changes across the lifespan.

Author response 1.

We agree and have softened the Abstract accordingly. The revised final sentence now reads: “These results, obscured by EduYears metrics, indicate a dynamic genetic architecture across educational milestones and suggest that attenuation at later transitions should be interpreted in the context of conditional progression and increasingly selected at-risk groups.”

Reviewer Response 1

The quoted section of the abstract does not match what is currently found in the authors abstract. However, whilst the authors now appropriately acknowledge selection/range restriction, they still infer “dynamic genetic architecture” from patterns that may arise largely from conditioning on prior attainment. Their revised interpretation does soften the claim, but it still does not establish that the observed attenuation reflects distinct genetic influences rather than expected statistical consequence of selection.

Reviewer Comment 2

The authors seem very keen to frame their findings in terms of biology rather than an artifact of the methods that they have used. For example, in figure 2 we find what is described as transition-specific results comparing the genetic correlations between each level of education conditioned on the previous levels, with years of Education and intelligence. The paragraph above reads as follows “The pattern shifted at postgraduate levels. BSc→MSc+ showed diminished correlations with EduYears (rg ≈ 0.82 [0.69–0.94]) and GCA (rg ≈ 0.56 [0.45–0.66]), though these remained substantial. MSc→PhD marked the sharpest decline, with values far below our benchmarks for EduYears (rg ≈ 0.38 [0.13–0.64]) and GCA (rg ≈ 0.28 [0.07–0.50]), indicating reduced alignment with the genetic influences captured by EA4 and IQ3, though with wider confidence intervals at this transition.”.

This wording invites a biological interpretation of the results of their work (i.e. completing a PhD is less genetically related to education/ intelligence or) rather than the more simple explanation of the genetic correlation is attenuated because of range restriction (i.e. those who complete a PhD are typically of a higher level of intelligence than those who do not).

The population of those who complete a PhD are highly selected on intelligence and EA linked traits as well as corelated social factors. This analysis would induce a restriction in the range of intelligence and EA related genetic variance as well as induce collider bias. Under these conditions a lower genetic correlation with intelligence and education is expected even if the same genetic influences were acting because the variance in intelligence genetic signal is compressed due the restriction. The authors need to be very clear that these results are likely selection-induced attenuation rather than implying intelligence maters less for attaining a PhD.

Similarly, the phrasing of “This contrast points to a two-component architecture: a shared general component that persists across most milestones and stage-specific components that increasingly differentiate higher levels” is also misleading as this too can simply be attributed to restricting the variance as described above.

Author Response 2.

Response: We agree and have substantially revised the interpretation.

(a) In the Results section describing Figure 2, we now write: “MSc→PhD showed the

weakest overlap with both EduYears (rg ≈ 0.38 [0.13–0.64]) and intelligence (rg ≈ 0.28

[0.07–0.50]), but these estimates were also markedly less precise. The substantially

smaller effective sample size at MSc→PhD (Neff = 8,185; Table 1) likely contributes to

this imprecision and should be considered alongside the attenuation itself.”

(b) In the Discussion we now devote two paragraphs to selection within the at-risk sets

as the primary account: “The weaker predictive performance, lower genetic correlations,

and reduced observed-scale signal at the highest milestones should therefore not be

interpreted as evidence that these traits matter less there. Rather, they are more

consistent with those traits having already contributed to selection into the at-risk group,

leaving less residual trait-related variance with which to distinguish who advances

further.”

(c) The "two-component architecture" sentence has been deleted entirely. We now frame

the cumulative rg gradient primarily in terms of selection and range restriction at later

milestones, while noting that the pattern remains compatible with substantial shared

genetic influences and some genuine differentiation at higher levels.

(d) The new supplementary PGI analyses reinforce this interpretation: mean PGI values

for intelligence-related traits are progressively higher at later milestones for both

advancers and non-advancers, indicating that these later transitions are drawn from

increasingly selected groups (Supplementary Fig. S39; Table S14).

Reviewer Response 2.

(a) Great

(b) Great

(c) & (d). The finding that the PGI rises for both advancers and non-advances is, as the authors state, very likely due to the increasingly selected groups. It is consistent with my initial concern that, rather than a dynamic genetic architecture across education milestones, there is an increasingly smaller range of ability at the higher levels of educational attainment. This supplementary analysis reinforces a statistical explanation of the authors results more than the “dynamic architecture” interpretation.

To illustrate my point, I’ve created a script where every genetic predictor has the same statistical effect at every educational transition. Thus, any weakening seen later therefore cannot be because the PGI “matters less”; it must come from selection/range restriction.

The simulation shows that attenuation in transition-specific PGI prediction or genetic correlations can arise simply from conditioning on prior educational success. Because the same genetic effect is imposed at every transition, the apparent weakening at later transitions cannot be interpreted as evidence that genetic influences are less important at higher educational levels. It is a consequence of sequential selection and range restriction.

From my simulations restriction alone produces highly similar effects to those found by the authors. I have included these simulations at the end of this review.

Reviewer Comment 3

This is related to my prior comment. These results are best described as genetic associations with progression of education, conditional on having passed earlier grades/levels not as the genetics of education level in general.

Author Response 3

We agree and have implemented this throughout. Transition-specific results are

now consistently described as “genetic associations with educational progression conditional

on prior attainment” rather than as the genetics of educational levels in general.

Reviewer Response 3

Great.

Reviewer Comment 4

The authors use the reference of Savage et al for their measure of IQ. I have a number of concerns with this data set. Specifically, the measure of intelligence used is the verbal numerical reasoning test in UK Biobank which is conditioned phenotypically on socioeconomic status. Conditioning on heritable phenotypes can result in collider bias but more worryingly for this study is that

Furthermore, the authors refer to this as IQ3. It’s not clear why they have selected this as the name of the trait as there multiple GWAS on intelligence. The first large scale GWAS of intelligence (using the VNR) being Hill et al 2018 (N = 248,482), the Savage GWAS (n = 269,867) used the Hill data to replicate their findings. However, the largest GWAS of intelligence is Lam et al 2021 with 373,617 participants. As such, I don’t really understand why the authors have selected this data set nor why they believe it is the third GWAS on intelligence.

Author Response 4

We have made three changes:

(a) We have replaced the “IQ3” label throughout the manuscript with “intelligence

(Savage et al., 2018),” which is a neutral and accurate description.

(b) We retained Savage et al. (2018) as our primary intelligence reference because it

indexes cognitive ability without incorporating educational attainment signals via MTAG,

which is important given that our study specifically aims to disentangle genetic influences

on education from those on intelligence. We have added Lam et al. (2021; N = 373,617)

as a sensitivity analysis. The sensitivity figure (Supplementary Fig. S27) shows that the

Lam et al. reference yields nearly identical rg trajectories across transitions, with the

same early high-overlap pattern and attenuation at MSc→PhD.

(c) Regarding the concern about SES conditioning in the UK Biobank VNR measure: the

convergence between the Savage and Lam results in our sensitivity an

Reviewer Response 4

(a) Great
(b) & (c) The MTAG data set to which the authors refer has already been used to disentangle genetic influences on education from those of intelligence^1,2^. The Lam et al data set consists of Savage + Davies. As such the effect of conditioning on ES is still present.

Reviewer Comment 5

Could the authors show if their estimates of the genetic correlations between educational milestones and intelligence and education differ significantly. At present the authors primarily rely on visual inspection and descriptive language to interpret differences across traits or transitions (Figures 2 and 5). As such, claims of lower rgs or of distinct genetic architectures should be interpreted cautiously.

Author Response 5

We conducted formal block-jackknife pairwise tests (200 blocks) across transitions for each reference trait, with within-trait FDR correction. These formal tests confirm that the attenuation centered on MSc→PhD is statistically significant, not merely a visual impression. Key results: • For EduYears (EA4): all three contrasts involving MSc→PhD were FDR-significant at q < 0.001; HS→BSc+ also differed from BSc→MSc+ at q < 0.05. • For intelligence (Lam et al., 2021): the three MSc→PhD contrasts were FDRsignificant at q < 0.01. • For CogSub, NonCogSub, and EduFields PCs: additional pairwise tests are provided. Results are presented in compact significance matrices (Supp

Reviewer Response 5

Great stuff.

Reviewer Comment 6

The authors make strong claims regarding the role of genetics in education. As is common in such papers, I strongly advise the authors write a detailed FAQ document that details what they found, what it means and importantly what it does not mean, and draft this for a lay audience. Manuscripts such as these are likely to picked up by the media where they can be (sometimes wilfully) misinterpreted.

Author Response 6

We appreciate this suggestion and have prepared a detailed FAQ document structured around four sections: (1) Background, (2) Key findings, (3) What the results do NOT mean, and (4) Broader context. The FAQ addresses common misinterpretations including genetic determinism, individual prediction, group differences, and policy implications. It is included as Supplementary Note 3 in the Supplementary Information, with a pointer in the Discussion.

Reviewer Response 6.

Nice.

References

1.	Anderson, E.L. *et al.* Education, intelligence and Alzheimer's disease: evidence from a multivariable two-sample Mendelian randomization study. *Int J Epidemiol* **49**, 1163-1172 (2020).

2.	Davies, N.M. *et al.* Multivariable two-sample Mendelian randomization estimates of the effects of intelligence and education on health. *Elife* **8**(2019).

##Simulations

\# Simulate how increasing selection/range restriction can produce

\# attenuated genetic associations across educational transitions,

\# even when the true PGI effect is constant at every stage.

set.seed(123)

library(dplyr)

library(ggplot2)

N <- 500000

\# Genetic predictor, e.g. intelligence/EA PGI

PGI <- rnorm(N)

\# Other influences on educational progression

\# These are independent of PGI, but affect progression too.

E1 <- rnorm(N)

E2 <- rnorm(N)

E3 <- rnorm(N)

E4 <- rnorm(N)

\# True PGI effect is CONSTANT at every transition

beta_g <- 0.7

beta_e <- 0.7

\# Latent liability to pass each stage

\# Same genetic effect at every stage i.e. PGI contributes equally to high school, bachelor’s, master’s, and PhD progression.

L1 <- beta_g * PGI + beta_e * E1 + rnorm(N)

L2 <- beta_g * PGI + beta_e * E2 + rnorm(N)

L3 <- beta_g * PGI + beta_e * E3 + rnorm(N)

L4 <- beta_g * PGI + beta_e * E4 + rnorm(N)

\# Thresholds chosen so later transitions become increasingly selective

\# among those still "at risk"

t1 <- quantile(L1, 0.25) # most pass high school

t2 <- quantile(L2, 0.55)

t3 <- quantile(L3, 0.75)

t4 <- quantile(L4, 0.90) # few reach PhD

HS	<- L1 > t1

BSc <- HS & L2 > t2

MSc <- BSc & L3 > t3

PhD <- MSc & L4 > t4

dat <- data.frame(PGI, HS, BSc, MSc, PhD)

\# Function for transition-specific associations

transition_summary <- function(data, at_risk, outcome, label) {

d <- data[data[[at_risk]], ]

y <- d[[outcome]]

fit <- glm(y ~ PGI, data = d, family = binomial())

tibble(

transition = label,

n_at_risk = nrow(d),

n_advance = sum(y),

advance_rate = mean(y),

mean_PGI_at_risk = mean(d$PGI),

sd_PGI_at_risk = sd(d$PGI),

mean_PGI_advanced = mean(d$PGI[y == 1]),

mean_PGI_not_advanced = mean(d$PGI[y == 0]),

PGI_mean_difference = mean(d$PGI[y == 1]) - mean(d$PGI[y == 0]),

log_OR_PGI = coef(fit)["PGI"],

OR_PGI = exp(coef(fit)["PGI"]),

pseudo_R2_Tjur = mean(fitted(fit)[y == 1]) - mean(fitted(fit)[y == 0])

)

}

\# Create explicit at-risk indicators

dat$All <- TRUE

res <- bind_rows(

transition_summary(dat, "All", "HS", "Comp -> HS"),

transition_summary(dat, "HS", "BSc", "HS -> BSc"),

transition_summary(dat, "BSc", "MSc", "BSc -> MSc"),

transition_summary(dat, "MSc", "PhD", "MSc -> PhD")

)

print(res)

\### This table shows the following

########## mean_PGI_at_risk increases across transitions

#########sd_PGI_at_risk decreases across transitions

###########PGI_mean_difference often shrinks at later stages

###########pseudo_R2_Tjur often attenuates at later stages

\##### that does not mean the PGI became less relevant for PhD attainment. It means the MSc-at-risk group is already genetically selected.

\# Plot 1: PGI variance shrinks in later at-risk groups

res <- res %>%

mutate(transition = factor(transition, levels = c(

"Comp -> HS",

"HS -> BSc",

"BSc -> MSc",

"MSc -> PhD"

)))

ggplot(res, aes(x = transition, y = sd_PGI_at_risk, group = 1)) +

geom_point(size = 3) +

geom_line() +

theme_minimal(base_size = 14) +

labs(

title = "Range restriction: PGI variance shrinks in later at-risk groups",

x = "Educational transition",

y = "SD of PGI among at-risk individuals"

)

########## This should go down. It shows range restriction. Later groups contain less PGI variation.

\# Plot 2: PGI prediction can attenuate despite constant true PGI effect

ggplot(res, aes(x = transition, y = pseudo_R2_Tjur, group = 1)) +

geom_point(size = 3) +

geom_line() +

theme_minimal(base_size = 14) +

labs(

title = "PGI prediction attenuates under sequential selection",

subtitle = "The true PGI effect is identical at every transition",

x = "Educational transition",

y = "Tjur pseudo-R2"

)

#########################This can go down even though the PGI effect is identical at every transition. This is the key demonstration against overinterpreting attenuation.

\# Plot 3: Mean PGI rises across selected groups

mean_levels <- tibble(

stage = c("Population", "Completed HS", "Completed BSc", "Completed MSc", "Completed PhD"),

mean_PGI = c(

mean(dat$PGI),

mean(dat$PGI[dat$HS]),

mean(dat$PGI[dat$BSc]),

mean(dat$PGI[dat$MSc]),

mean(dat$PGI[dat$PhD])

),

sd_PGI = c(

sd(dat$PGI),

sd(dat$PGI[dat$HS]),

sd(dat$PGI[dat$BSc]),

sd(dat$PGI[dat$MSc]),

sd(dat$PGI[dat$PhD])

)

) %>%

mutate(stage = factor(stage, levels = c(

"Population",

"Completed HS",

"Completed BSc",

"Completed MSc",

"Completed PhD"

)))

print(mean_levels)

ggplot(mean_levels, aes(x = stage, y = mean_PGI, group = 1)) +

geom_point(size = 3) +

geom_line() +

theme_minimal(base_size = 14) +

labs(

title = "Mean PGI increases across educational milestones",

subtitle = "Even though the PGI effect is constant at each transition",

x = "Group",

y = "Mean PGI"

) +

theme(axis.text.x = element_text(angle = 30, hjust = 1))

##################### This should go up from population to HS to BSc to MSc to PhD. That shows repeated selection.

plotdat <- bind_rows(

data.frame(PGI = dat$PGI, group = "Population"),

data.frame(PGI = dat$PGI[dat$HS], group = "Completed HS"),

data.frame(PGI = dat$PGI[dat$BSc], group = "Completed BSc"),

data.frame(PGI = dat$PGI[dat$MSc], group = "Completed MSc"),

data.frame(PGI = dat$PGI[dat$PhD], group = "Completed PhD")

) %>%

mutate(group = factor(group, levels = c(

"Population",

"Completed HS",

"Completed BSc",

"Completed MSc",

"Completed PhD"

)))

ggplot(plotdat, aes(x = PGI)) +

geom_density() +

facet_wrap(~ group, ncol = 1) +

theme_minimal(base_size = 14) +

labs(

title = "Sequential selection shifts and narrows the PGI distribution",

x = "PGI",

y = "Density"

)

############## This shows the PGI distribution shifting rightward and often narrowing as milestones accumulate.
