# Supplementary material for: Beyond years of schooling: Genetic associations across educational milestones in two Norwegian cohorts
Source: PLoS Genet. 2026 Sep 17;22(9):e1012310. doi: 10.1371/journal.pgen.1012310 (PMC13596786; doi:10.1371/journal.pgen.1012310)
Supplement: Supplementary file 6 [file pgen.1012310.s008.docx]

**Response to Reviewers – Second Revision**

PGENETICS-D-25-01264R1

*Beyond years of schooling: Genetic associations across educational milestones in two Norwegian cohorts*

Kvalvik, Wang, Walhovd, Lyngstad & Røgeberg

Dear Editors and Reviewers,

We thank the Editors and Reviewer #1 for the additional careful evaluation of our revised manuscript, and Reviewers #2 and #3 for confirming that their concerns have been addressed. The central issue in this round was whether attenuation across educational milestones could be interpreted as evidence of distinct genetic architecture, given that sequential selection, range restriction, and conditioning on prior attainment can produce similar patterns. We agree that our earlier framing remained too strong in places. We have therefore revised the manuscript so that selection is treated as a major interpretive consideration, while the relative contributions of selection and transition-specific differences in conditional association remain unresolved.

Reviewer and Editor comments are shown in shaded boxes; our responses follow in plain text, and quotations from the revised manuscript are shown indented. Because several of Reviewer #1’s comments in this round are brief replies to our earlier responses, we first recap the original exchange in italics so that each point is self-contained. All references are to the revised manuscript with tracked changes.

**Summary of the changes in this revision**

In this revision, we made five main changes.

**First,** we revised the title from “Beyond years of schooling: Shifting genetic influences across educational milestones in two Norwegian cohorts” to “Beyond years of schooling: Genetic associations across educational milestones in two Norwegian cohorts,” and removed or softened language implying a “dynamic genetic architecture” or distinct genetic effects at different educational stages. Throughout the Abstract, Results, Discussion, and Methods we now emphasize genetic associations, milestone definitions, conditional progression, selection, case composition, and the limits of mechanistic interpretation.

**Second,** we adapted and expanded Reviewer #1’s simulation into a new Supplementary Note 4, with full annotated R code and two new figures (Supplementary Figs. S44–S45). The simulation demonstrates that increasing mean PGI among later at-risk groups, declining PGI variance, and attenuated transition-specific discrimination can arise even when the same simulated PGI contributes to every transition with the same coefficient. We also use the simulation to distinguish mean PGI enrichment, observed-scale discrimination measured by Tjur’s R^2^, and per-SD odds ratios, because these quantities respond differently to range restriction and case composition. We describe the simulation as an illustrative constant-coefficient scenario, not as a fitted generative model of educational attainment.

**Third,** we revised the Discussion to clarify that the observed attenuation is not uniquely interpretable within the present design. The simulation directly shows that transition-specific PGI discrimination can decline even when the simulated PGI coefficient is held constant. We also state that related conditioning and case-composition concerns bear on transition-specific h^2^_SNP_ and r_g_, while noting that the simulation does not directly evaluate those quantities. Finally, we distinguish repeated at-risk conditioning in the transition-specific framework from cumulative attained-status analyses in the full cohort.

**Fourth,** we clarified the interpretation of the EA4 and intelligence reference traits, noting that EduYears and intelligence are not cleanly separable and that the Lam et al. sensitivity analysis does not resolve education–intelligence entanglement.

**Fifth,** we updated the Supplementary Information, Supporting Information captions, Code Availability statement, and figure and table inventory to include Supplementary Note 4 and Supplementary Figs. S44–S45. We are grateful to Reviewer #1 for providing the original simulation script. In accordance with PLOS Genetics’ guidance, this contribution is acknowledged in the present response letter rather than in the manuscript or Supplementary Information.

**Additional Editor Comments**

***Editor:*** *While Reviewers #2 and #3 were satisfied with the authors’ responses and the revised manuscript, Reviewer #1 raised important issues with the results presented and provided a simulation to support his concerns regarding potential confounders due to selection and range restriction. I would like the authors to carefully consider this information, provide a detailed response, and update their manuscript accordingly.*

**Response:** We thank the Editor for this guidance. We agree that Reviewer #1’s simulation illustrates that transition-specific PGI discrimination can attenuate even when the simulated PGI coefficient is held constant. We incorporated this point directly through Supplementary Note 4 and Supplementary Figs. S44–S45, revised the Discussion to state that attenuation cannot by itself separate changing conditional associations from prior selection and case composition, removed the “dynamic genetic architecture” framing, and revised terminology throughout the Methods, Results, and Discussion.

**Reviewer #1**

We thank Reviewer #1 for the constructive second-round review and for providing the simulation script, which helped us identify places where the manuscript still overinterpreted attenuation across milestones.

**Comment 1 — Strength of the Abstract’s final sentence**

*Round 1 — Reviewer #1 noted that the final sentence of the Abstract was too strong, since the heritability of cognitive ability is known to change across the lifespan. We softened the Abstract but retained a “dynamic genetic architecture” framing.*

***Reviewer #1 (this round):*** *The quoted section of the abstract does not match what is currently found in the authors abstract. However, whilst the authors now appropriately acknowledge selection/range restriction, they still infer “dynamic genetic architecture” from patterns that may arise largely from conditioning on prior attainment. Their revised interpretation does soften the claim, but it still does not establish that the observed attenuation reflects distinct genetic influences rather than expected statistical consequence of selection.*

**Response:** We agree. We have removed the “dynamic genetic architecture” framing from the Abstract and softened the interpretation throughout the manuscript. The revised Abstract now describes the findings in terms of observed genetic associations, explicitly notes the role of selection at the doctoral transition, and emphasizes the measurement implications of collapsing credentials into a single EduYears scale. It now concludes:

*“At the doctoral transition, estimates were obtained among individuals who had attained at least a master’s degree, so attenuation there must be interpreted in light of selection and range restriction. Because EduYears converts distinct credentials and progression steps into a single numerical scale, estimates expressed per additional year should not be interpreted as though one year has the same meaning across the educational sequence. Milestone-preserving analyses make that hidden structure visible and sharpen the interpretation of education GWAS and PGIs.”*

This revised wording no longer interprets attenuation as evidence of distinct genetic influences across stages, while retaining the main descriptive contribution of the milestone-based framework.

We also revised the Discussion to clarify that the observed composition and magnitude patterns are not uniquely interpretable within the present design:

*“These composition and magnitude patterns can arise from several sources, including external phenotype coding, case composition, and sequential selection. Selection is particularly relevant to the transition-specific estimates because later contrasts are defined within progressively selected at-risk groups. However, the present design cannot quantify how much of the observed variation selection explains or exclude residual milestone-specific differences in conditional association.”*

The Discussion therefore no longer treats attenuation as direct evidence that distinct genetic effects operate at different educational stages. Instead, it presents external measurement, case composition, sequential selection, statistical precision, and residual transition-specific differences in conditional association as potentially contributing explanations. We also distinguish the repeated conditioning inherent in the transition-specific framework from cumulative attained-status analyses conducted in the full cohort, which are less directly exposed to this specific mechanism but remain sensitive to endpoint prevalence and case imbalance.

**Comment 2 — Selection and range restriction versus “dynamic architecture”; the simulation**

*Round 1 — Reviewer #1 cautioned that a biological reading of the attenuation (e.g., a “two-component architecture”) overinterprets a pattern that is expected under range restriction in increasingly selected groups. We reframed the results around selection, deleted the “two-component architecture” sentence, and added supplementary PGI analyses.*

***Reviewer #1 (this round):*** *The finding that the PGI rises for both advancers and non-advancers is, as the authors state, very likely due to the increasingly selected groups. It is consistent with my initial concern that, rather than a dynamic genetic architecture across education milestones, there is an increasingly smaller range of ability at the higher levels of educational attainment. This supplementary analysis reinforces a statistical explanation of the authors results more than the “dynamic architecture” interpretation.*

*To illustrate my point, I’ve created a script where every genetic predictor has the same statistical effect at every educational transition. Thus, any weakening seen later therefore cannot be because the PGI “matters less”; it must come from selection/range restriction. The simulation shows that attenuation in transition-specific PGI prediction or genetic correlations can arise simply from conditioning on prior educational success. Because the same genetic effect is imposed at every transition, the apparent weakening at later transitions cannot be interpreted as evidence that genetic influences are less important at higher educational levels. It is a consequence of sequential selection and range restriction.*

*From my simulations restriction alone produces highly similar effects to those found by the authors. I have included these simulations at the end of this review.*

**Response:** We agree with the reviewer’s central interpretive point, and we thank Reviewer #1 for providing the simulation script. We adapted and expanded the script into Supplementary Note 4, with full annotated R code and two new figures (Supplementary Figs. S44–S45). The simulation holds the coefficient of the simulated PGI constant across all four transitions. Despite this, sequential thresholding raises the mean PGI and reduces PGI variance in later at-risk groups. Together with endpoint case imbalance, this produces attenuation in transition-specific PGI discrimination.

We have also revised the manuscript to distinguish carefully between what the simulation demonstrates directly and the broader interpretive implications. The simulation directly evaluates PGI discrimination under a constant simulated coefficient. It does not directly model genetic correlations or SNP heritability. The revised Discussion now states:

*“Supplementary Note 4 illustrates this point directly for transition-specific PGI discrimination. With the simulated PGI coefficient held constant across transitions, sequential thresholding raises mean PGI and reduces PGI variance in later at-risk groups. Together with endpoint case imbalance, this lowers Tjur’s R^2^, while the per-SD odds ratio remains approximately stable (Supplementary Figs. S44-S45). The simulation is an illustrative scenario rather than a model fitted to the MoBa data, and it does not directly model genetic correlations or h^2^_SNP_. It shows that declining transition-specific PGI discrimination is compatible with a constant PGI coefficient and therefore cannot, by itself, be attributed to weaker conditional associations at later milestones. Related conditioning and case-composition concerns also bear on the interpretation of transition-specific genetic correlations and observed-scale h^2^_SNP_, although those quantities are not directly evaluated by the simulation.”*

We further clarified that mean PGI, Tjur’s R^2^, and per-SD odds ratios describe different aspects of the selected at-risk groups and should not be interpreted interchangeably. The revised Discussion states:

*“The changing composition of these at-risk sets is visible in the supplementary PGI analyses. For the intelligence-related, CogSub, and NonCogSub PGIs, mean scores increased across transitions among both advancers and non-advancers, indicating that later comparisons are drawn from groups increasingly enriched for education-related, cognitive, and non-cognitive genetic propensities (Supplementary Fig. S39). Mean PGI, Tjur’s R^2^, and the per-SD odds ratio capture different features of these selected groups. Mean PGI describes the composition of the at-risk set. Tjur’s R^2^ quantifies observed-scale discrimination between advancers and non-advancers within that set. The odds ratio estimates the conditional association of a one-SD higher PGI with advancement. These quantities should therefore not be interpreted interchangeably.”*

The empirical odds ratios for the intelligence-related and GWAS-by-subtraction PGIs tended to decline across later transitions, whereas the odds ratio remained approximately stable in the constant-coefficient simulation. We therefore do not attribute the empirical odds-ratio attenuation to range restriction alone.

The manuscript now interprets these trajectories as compatible with selection-induced dependence, residual transition-specific differences in conditional association, or both, while stating that the present design cannot separate these possibilities.

Finally, we removed language implying that weaker observed signal means that genetic factors matter less at later educational stages. The revised manuscript instead states that later transition-specific attenuation is compatible with sequential selection and case composition, while the relative contributions of selection and residual transition-specific differences remain unresolved. The milestone framework is therefore presented as revealing how observed genetic associations and PGI discrimination depend on the educational contrast and the population being compared, rather than as demonstrating a dynamic genetic architecture.

**Comment 3 — Framing transition-specific results as conditional associations**

*Round 1 — Reviewer #1 asked that transition-specific results be described as genetic associations with progression conditional on prior attainment, not as the genetics of educational level in general. We implemented this throughout.*

***Reviewer #1 (this round):*** *Great.*

**Response:** We are grateful the reviewer is satisfied. We have retained this conditional-progression framing throughout the Methods, Results, and Discussion.

**Comment 4 — Choice and entanglement of the intelligence reference**

*Round 1 — Reviewer #1 questioned the “IQ3” label and our use of Savage et al. (2018) rather than the larger Lam et al. (2021). We relabelled the trait, retained Savage et al. as the primary reference, and added Lam et al. (2021) as a sensitivity analysis.*

***Reviewer #1 (this round):*** *(a) Great. (b) & (c) The MTAG data set to which the authors refer has already been used to disentangle genetic influences on education from those of intelligence. The Lam et al data set consists of Savage + Davies. As such the effect of conditioning on ES is still present.*

**Response:** We thank the reviewer for this clarification and agree that the Lam et al. sensitivity analysis neither eliminates the strong coupling between education and intelligence nor provides a cleanly independent intelligence comparator. We therefore no longer present it as separating education-related from intelligence-related genetic signal; it now appears only as a sensitivity reference showing that the broad milestone trajectory is similar under an alternative intelligence GWAS. The revised Discussion states:

*“EA4 and intelligence should also be treated as overlapping rather than orthogonal reference traits, given their strong phenotypic and genetic coupling [36,37].”*

We further clarify that the GWAS-by-subtraction cognitive and non-cognitive components were constructed jointly from the EA3 educational-attainment and cognitive-performance GWASs, both reported by Lee et al. (2018), and therefore do not provide a comparison fully independent of the EduYears GWAS lineage underlying EA4. The cited references, Davies et al. (2019) and Anderson et al. (2020), have been added to the reference list.

**Comment 5 — Formal tests for differences in genetic correlation**

*Round 1 — Reviewer #1 asked us to test formally whether genetic correlations differ across transitions rather than relying on visual inspection. We added block-jackknife pairwise tests with within-trait FDR correction.*

***Reviewer #1 (this round):*** *Great stuff.*

**Response:** We are grateful the reviewer is satisfied. We have retained the block-jackknife pairwise tests of r_g_ differences (within-trait FDR correction; Supplementary Figs. S28, S30, and S32 and Supplementary Tables S4–S9) and now interpret the significant differences more cautiously. Statistically significant attenuation in r_g_ does not by itself identify distinct genetic effects across milestones, because the estimates may also be shaped by sequential selection, range restriction, case composition, and reduced residual variation in later at-risk groups.

**Comment 6 — Lay-audience FAQ**

*Round 1 — Reviewer #1 advised preparing a detailed lay-audience FAQ describing what the findings do and do not mean. We added this as Supplementary Note 3.*

***Reviewer #1 (this round):*** *Nice.*

**Response:** We are grateful the reviewer is satisfied. We have retained the FAQ (Supplementary Note 3) and revised it to match the updated framing. It now emphasizes that the results are associational, do not identify “genes for” particular degrees, are unsuitable for individual-level prediction or admissions decisions, and that milestone differences cannot by themselves distinguish changing conditional associations from attenuation related to prior selection, case composition, or measurement.

**Reviewers #2 and #3**

***Reviewer #2:*** *Thank you for your thorough response. The revisions have addressed my concerns and I have no further comments.*

***Reviewer #3:*** *Thank you for addressing my comments and reflecting on my suggestions.*

**Response:** We thank Reviewers #2 and #3 for confirming that their concerns have been addressed. We have made no changes that affect the analyses they reviewed.

**Additional consistency updates**

We also made consistency updates to align the manuscript and Supplementary Information with the revised framing. We revised the title from “Shifting genetic influences” to “Genetic associations,” described the transition-specific analyses as conditional progression and the cumulative analyses as attained status, and distinguished mean PGI composition from Tjur’s R^2^ and per-SD odds ratios. We also revised the lay FAQ to explain that weaker observed signal at the doctoral stage does not imply that genetics matters less for doctoral completion. Supplementary Note 4, Supplementary Figs. S44–S45, the Supporting Information captions, the Code Availability statement, and the figure and table inventory have all been updated accordingly. The reviewer’s contribution to the original simulation is documented in this response letter rather than in the manuscript or Supplementary Information.

We believe these revisions directly address the remaining concerns raised by the Editor and Reviewer #1. The manuscript no longer presents attenuation across milestones as evidence for a dynamic genetic architecture. Instead, it concludes that the milestone framework reveals variation in observed genetic association and PGI discrimination that a single EduYears phenotype compresses. It also states clearly that later transition-specific attenuation is compatible with sequential selection and should not be interpreted as showing that education-related or cognitive propensities matter less at higher stages.

Sincerely,

Eirik H. Kvalvik

on behalf of all authors
