# Supplementary material for: Beyond years of schooling: Genetic associations across educational milestones in two Norwegian cohorts
Source: PLoS Genet. 2026 Sep 17;22(9):e1012310. doi: 10.1371/journal.pgen.1012310 (PMC13596786; doi:10.1371/journal.pgen.1012310)
Supplement: Supplementary file 7 [file pgen.1012310.s009.docx]

Response to Reviewers – Minor Revision

**PGENETICS-D-25-01264R2**

Beyond years of schooling: Genetic associations across educational milestones in two Norwegian cohorts

Kvalvik, Wang, Walhovd, Lyngstad & Røgeberg

Oslo, 21 August 2026

Dear Editors and Reviewer #1,

We thank the Editors and Reviewer #1 for the further careful assessment of our manuscript. We made fourteen focused textual edits in response to the remaining comments: two in the Results, seven in the Discussion, and five in the Supplementary Information (three in the FAQ and two in Supplementary Note 4). These revisions report the empirical odds-ratio pattern and clarify the interpretation of the lower MSc→PhD genetic correlations in the Results; refine the Discussion’s treatment of sequential selection, odds-ratio attenuation, limitations, and future research, including why changing transition-specific associations do not by themselves imply changing underlying genetic effects; address the remaining concern regarding the intelligence reference GWASs; and align the corresponding FAQ and simulation text. No analyses or Methods were changed, and the title, Abstract, and Author Summary remain unchanged.

Reviewer comments are reproduced below as relevant excerpts in shaded boxes; our responses follow in plain text, and quotations from the revised manuscript are indented. Line numbers refer to the revised manuscript with track changes. Supplementary Information locations are given by section and page.

# Response to the Editor

*“While the manuscript has been significantly improved, few remaining comments from Reviewer #1 should be addressed.”*

We have addressed each remaining point through focused interpretive and reporting revisions. The changes are detailed below.

# Reviewer #1

## Comment 1 — Scope of the illustrative selection simulation

*Relevant excerpt: “I would say that the analyses do not only fail to separate selection from milestone-specific genetic effects but also that they do not separate simple selection from more realistic forms of selection. The current simulation represents one illustrative constant effect model. It does not establish that more complex constant effect models would necessarily fail to reproduce the observed attenuation. Consequently, the manuscript still leaves the impression that there may be a residual milestone-specific component once selection has been considered, whereas the evidence does not distinguish this possibility from other selection mechanisms.”*

Response: We agree. We removed the Discussion-roadmap sentence that introduced residual milestone-specific differences as a co-equal explanation before the selection argument was developed (deleted at lines 440–441). To keep the distinction between observed association patterns and possible mechanisms consistent in the Results, we also revised the interpretation of the lower MSc→PhD genetic correlations (lines 218–223):

Because this transition is estimated within an already highly selected MSc+ at-risk group, the lower correlations should not be interpreted as showing that education- or intelligence-related traits matter less for doctoral attainment. Selection into this at-risk group may restrict the residual variation in education-related characteristics available to distinguish further progression.

We also clarified the scope of the illustrative simulation in the Discussion (lines 513–518):

The approximately stable odds-ratio trajectory is a feature of this particular simulation and should not be interpreted as a general prediction of constant-coefficient selection processes; more realistic forms of sequential selection could alter conditional odds ratios without any change in the underlying PGI coefficient. The simulation’s central implication is therefore narrower: declining transition-specific PGI discrimination can occur even when the underlying PGI coefficient is constant across transitions.

Two further Discussion edits keep the same conceptual levels separate. The limitations paragraph now states that prior selection can alter later conditional associations by changing the composition and residual variation of at-risk groups, while the design cannot determine how much of the observed attenuation is attributable to this process (lines 589–593). The future-research paragraph now describes common-factor models as characterizing the broad shared component and any residual milestone-specific associations, rather than as distinguishing selection from residual association; any residual pattern would still need to be interpreted in light of selection (lines 610–617).

We made two corresponding, deliberately limited edits in Supplementary Note 4. In the Interpretation section, the fourth pattern now states only that PGI discrimination can attenuate while the simulated odds-ratio trajectory remains comparatively stable (p. 66); the earlier causal clause linking OR stability to the identical coefficient was removed. The code comment introducing the OR plot is now descriptive rather than predictive (p. 60). We did not add a claim that a particular alternative model must reproduce the empirical OR decline.

The same hierarchy is stated in lay terms in FAQ 2.2 (pp. 44–45):

The transition-specific attenuation is therefore compatible with sequential selection and does not, by itself, show that underlying genetic effects differ across stages. Such differences remain possible, but are not needed to explain the observed pattern.

## Comment 2 — Empirical odds ratios and the Discussion conclusion

*Relevant excerpt: “The empirical attenuation of the odds ratios is introduced in the Discussion as the principal reason why the illustrative simulation does not fully explain the observed data. However, these odds-ratio results are neither presented nor formally evaluated in the Results section. If the authors wish to rely on this observation as evidence that their empirical data differ from the illustrative simulation, then it should first be demonstrated formally. [...] I do not think it good evidence for the existence of milestone-specific genetic effects, but only evidence that the very simple selection modelled does not capture nuance. [...] The discussion could conclude something along the lines of: ‘The present analyses provide no direct evidence that transition-specific attenuation reflects milestone-specific genetic effects. Instead, the observed patterns are compatible with the sequential selection induced by educational progression. Although residual milestone-specific differences cannot be excluded, they are not required to explain the empirical observations.’”*

Response: We agree. The Results now report the empirical odds-ratio pattern directly (lines 302–312; the OR statement is at lines 306–309):

Mean PGI values nevertheless increased across transitions for both advancers and non-advancers, consistent with progressive selection into later at-risk sets, while the corresponding per-SD odds-ratio point estimates were generally lower at later transitions.

We no longer use the empirical-versus-simulated OR difference as evidence against a selection-based explanation. The sentence identified by the reviewer now reads (lines 496–499):

The empirical odds-ratio trajectories are therefore compatible with selection-induced dependence within later at-risk sets and do not, by themselves, imply that the underlying PGI effect changes across milestones.

We did not add a formal first-versus-last transition OR contrast. The reviewer made such a test conditional on retaining the empirical OR trajectory as counterevidence to the simulation, and we have withdrawn that inferential use. As the reviewer notes, even a significant contrast would not distinguish milestone-specific effects from richer selection processes. Supplementary Fig. S40 and Supplementary Table S14 therefore remain descriptive presentations of the existing OR estimates.

Finally, we revised the actual concluding paragraph of the Discussion (lines 620–634). Its central interpretive statement is (lines 626–628):

The attenuation observed in the transition-specific analyses is compatible with sequential selection induced by educational progression; milestone-specific changes in underlying genetic effects remain possible, but are neither demonstrated by nor necessary to explain this pattern.

FAQ 3.7 now distinguishes the transition-specific MSc→PhD comparison from the rare cumulative PhD outcome (p. 47), and FAQ 4.2 gives a concise general non-identification limitation (pp. 48–49).

## Comment 4 — Intelligence reference GWASs

*Relevant excerpt: “Second (b), the authors state they used Savage as it does not include education via MTAG. I argued that the Hill et al data [...] has subsequently been used to disentangle the effects of education and intelligence [...] so the author reluctance to use this data is unwarranted (but understandable). Third (c), Lam is a combination of Davies and Savage, as such the fluid intelligence data is conditioned phenotypically on the Townsend score. [...] If the findings are attributable to conditioning on Townsend in the Savage data then using the Lam data may very well result in the same confound producing the same results.”*

**Response (b):** We accept this correction. Our previous rationale for preferring Savage et al. because it did not incorporate educational-attainment information through MTAG was too categorical, and we withdraw it. As the reviewer notes, Davies et al. (2019) and Anderson et al. (2020) show that education and intelligence can be modeled jointly in multivariable Mendelian-randomization analyses to estimate their conditional contributions to downstream outcomes. We retain Savage et al. (2018) as the primary intelligence reference for continuity with the analyses reported here, not because MTAG-derived intelligence data are unsuitable. This rationale appeared only in our previous response, so no manuscript change was required.

**Response (c):** We agree that our previous response did not state the specific conditioning issue clearly enough. We retain Lam et al. (2021) as a sensitivity analysis to the choice of intelligence reference GWAS, but its overlapping inputs mean that Savage–Lam concordance is not an independent check on potential bias arising from the Townsend adjustment. The Discussion now states (lines 450–455):

A further limitation is that the UK Biobank fluid-intelligence component used in Savage et al. (2018) [26] was adjusted for socioeconomic status using the Townsend deprivation index. Because the Lam et al. (2021) [27] intelligence reference incorporates GWAS inputs overlapping with Savage et al., the similar trajectories obtained with Savage and Lam do not provide an independent check on potential bias arising from this phenotypic conditioning.

We thank the Editor and Reviewer #1 again for the constructive comments. We believe the revised manuscript now gives sequential selection the evidential priority supported by the simulation while retaining the paper’s descriptive contribution: milestone-preserving analyses reveal how genetic estimates depend on the educational contrast and comparison group, without treating transition-specific attenuation as evidence that underlying genetic effects must change across milestones.

Sincerely,

Eirik H. Kvalvik
on behalf of all authors
