# Supplementary material for: Beyond years of schooling: Genetic associations across educational milestones in two Norwegian cohorts
Source: PLoS Genet. 2026 Sep 17;22(9):e1012310. doi: 10.1371/journal.pgen.1012310 (PMC13596786; doi:10.1371/journal.pgen.1012310)
Supplement: Peer review history

# Peer review: Decision Letter 0

**DOI:** 10.1371/journal.pgen.1012310.r001

**Authors:** Hua Tang, Renato Polimanti

16 Feb 2026

PGENETICS-D-25-01264

Beyond years of schooling: Shifting genetic influences across educational milestones in two Norwegian cohorts

PLOS Genetics

Dear Dr. Kvalvik,

Thank you for submitting your manuscript to PLOS Genetics. After careful consideration, we feel that it has merit but does not fully meet PLOS Genetics's publication criteria as it currently stands. Therefore, we invite you to submit a revised version of the manuscript that addresses the points raised during the review process.

Please submit your revised manuscript by Apr 17 2026 11:59PM. If you will need more time than this to complete your revisions, please reply to this message or contact the journal office at plosgenetics@plos.org. When you're ready to submit your revision, log on to https://www.editorialmanager.com/pgenetics/ and select the 'Submissions Needing Revision' folder to locate your manuscript file.

Please include the following items when submitting your revised manuscript:

* A letter that responds to each point raised by the editor and reviewer(s). You should upload this letter as a separate file labeled 'Response to Reviewers'. This file does not need to include responses to any formatting updates and technical items listed in the 'Journal Requirements' section below.

* A marked-up copy of your manuscript that highlights changes made to the original version. You should upload this as a separate file labeled 'Revised Manuscript with Track Changes'.

* An unmarked version of your revised paper without tracked changes. You should upload this as a separate file labeled 'Manuscript'.

If you would like to make changes to your financial disclosure, competing interests statement, or data availability statement, please make these updates within the submission form at the time of resubmission. Guidelines for resubmitting your figure files are available below the reviewer comments at the end of this letter.

We look forward to receiving your revised manuscript.

Kind regards,

Renato Polimanti, Ph.D.

Academic Editor

PLOS Genetics

Hua Tang

Section Editor

PLOS Genetics

Aimée Dudley

Editor-in-Chief

PLOS Genetics

Anne Goriely

Editor-in-Chief

PLOS Genetics

**Additional Editor Comments:**

While most reviewers appreciated the overall goal of the study, they also highlighted several issues that should be addressed before the manuscript can be considered further. In particular, I would like to pay attention to Reviewer #1's comments about collider bias, the lack of formal comparison between the genetic correlations derived, and the need to more carefully discuss the meaning of the findings.

**Journal Requirements:**

If the reviewer comments include a recommendation to cite specific previously published works, please review and evaluate these publications to determine whether they are relevant and should be cited. There is no requirement to cite these works unless the editor has indicated otherwise.

1) We ask that a manuscript source file is provided at Revision. Please upload your manuscript file as a .doc, .docx, .rtf or .tex. If you are providing a .tex file, please upload it under the item type u2018LaTeX Source Fileu2019 and leave your .pdf version as the item type u2018Manuscriptu2019.

2) Please provide an Author Summary. This should appear in your manuscript between the Abstract (if applicable) and the Introduction, and should be 150-200 words long. The aim should be to make your findings accessible to a wide audience that includes both scientists and non-scientists. Sample summaries can be found on our website under Submission Guidelines:

https://journals.plos.org/plosgenetics/s/submission-guidelines#loc-parts-of-a-submission

3) Please upload all main figures as separate Figure files in .tif or .eps format. For more information about how to convert and format your figure files please see our guidelines:

https://journals.plos.org/plosgenetics/s/figures

4) We have noticed that you have uploaded Supporting Information files, but you have not included a list of legends. Please add a full list of legends for your Supporting Information files after the references list.

5) When completing the data availability statement of the submission form, you indicated that you will make your data available on acceptance. We strongly recommend all authors decide on a data sharing plan before acceptance, as the process can be lengthy and hold up publication timelines. Please note that, though access restrictions are acceptable now, your entire data will need to be made freely accessible if your manuscript is accepted for publication. This policy applies to all data except where public deposition would breach compliance with the protocol approved by your research ethics board. If you are unable to adhere to our open data policy, please kindly revise your statement to explain your reasoning and we will seek the editor's input on an exemption. Please be assured that, once you have provided your new statement, the assessment of your exemption will not hold up the peer review process.

6) Please amend your detailed Financial Disclosure statement. This is published with the article. It must therefore be completed in full sentences and contain the exact wording you wish to be published.

1) State the initials, alongside each funding source, of each author to receive each grant. For example: "This work was supported by the National Institutes of Health (####### to AM; ###### to CJ) and the National Science Foundation (###### to AM)."

2) State what role the funders took in the study. If the funders had no role in your study, please state: "The funders had no role in study design, data collection and analysis, decision to publish, or preparation of the manuscript."

3) If any authors received a salary from any of your funders, please state which authors and which funders..

If you did not receive any funding for this study, please simply state: u201cThe authors received no specific funding for this work.u201d

**Reviewers' comments:**

Reviewer's Responses to Questions

**Comments to the Authors:**

**Please note here if the review is uploaded as an attachment.**

Reviewer #1: Comment 1

The final sentence of the abstract is far too strong. I don’t believe any one has ever argued that genetic influences are uniform genetic influences across time or educational progression. There’s actually a lot of literature detailing how heritability of cognitive ability (which contributes substantially to the heritability of education) changes across the lifespan.

Comment 2

The authors seem very keen to frame their findings in terms of biology rather than an artifact of the methods that they have used. For example, in figure 2 we find what is described as transition-specific results comparing the genetic correlations between each level of education conditioned on the previous levels, with years of Education and intelligence. The paragraph above reads as follows “The pattern shifted at postgraduate levels. BSc→MSc+ showed diminished correlations with EduYears (rg ≈ 0.82 [0.69–0.94]) and GCA (rg ≈ 0.56 [0.45–0.66]), though these remained substantial. MSc→PhD marked the sharpest decline, with values far below our benchmarks for EduYears (rg ≈ 0.38 [0.13–0.64]) and GCA (rg ≈ 0.28 [0.07–0.50]), indicating reduced alignment with the genetic influences captured by EA4 and IQ3, though with wider confidence intervals at this transition.”.

This wording invites a biological interpretation of the results of their work (i.e. completing a PhD is less genetically related to education/ intelligence or) rather than the more simple explanation of the genetic correlation is attenuated because of range restriction (i.e. those who complete a PhD are typically of a higher level of intelligence than those who do not).

The population of those who complete a PhD are highly selected on intelligence and EA linked traits as well as corelated social factors. This analysis would induce a restriction in the range of intelligence and EA related genetic variance as well as induce collider bias. Under these conditions a lower genetic correlation with intelligence and education is expected even if the same genetic influences were acting because the variance in intelligence genetic signal is compressed due the restriction. The authors need to be very clear that these results are likely selection-induced attenuation rather than implying intelligence maters less for attaining a PhD.

Similarly, the phrasing of “This contrast points to a two-component architecture: a shared general component that persists across most milestones and stage-specific components that increasingly differentiate higher levels” is also misleading as this too can simply be attributed to restricting the variance as described above.

Comment 3

This is related to my prior comment. These results are best described as genetic associations with progression of education, conditional on having passed earlier grades/levels not as the genetics of education level in general.

Comment 4

The authors use the reference of Savage et al for their measure of IQ. I have a number of concerns with this data set. Specifically, the measure of intelligence used is the verbal numerical reasoning test in UK Biobank which is conditioned phenotypically on socioeconomic status. Conditioning on heritable phenotypes can result in collider bias but more worryingly for this study is that

Furthermore, the authors refer to this as IQ3. It’s not clear why they have selected this as the name of the trait as there multiple GWAS on intelligence. The first large scale GWAS of intelligence (using the VNR) being Hill et al 2018 (N = 248,482), the Savage GWAS (n = 269,867) used the Hill data to replicate their findings. However, the largest GWAS of intelligence is Lam et al 2021 with 373,617 participants. As such, I don’t really understand why the authors have selected this data set nor why they believe it is the third GWAS on intelligence.

Comment 5

Could the authors show if their estimates of the genetic correlations between educational milestones and intelligence and education differ significantly. At present the authors primarily rely on visual inspection and descriptive language to interpret differences across traits or transitions (Figures 2 and 5). As such, claims of lower rgs or of distinct genetic architectures should be interpreted cautiously.

Comment 6

The authors make strong claims regarding the role of genetics in education. As is common in such papers, I strongly advise the authors write a detailed FAQ document that details what they found, what it means and importantly what it does not mean, and draft this for a lay audience. Manuscripts such as these are likely to picked up by the media where they can be (sometimes wilfully) misinterpreted.

Reviewer #2: see attachment

Reviewer #3: This paper analyses the genetic architecture of educational achievement in Norway taking different approaches to see whether SNP-derived and twin-derived genetic effects vary according to the achieved academic degree, rather than based on year of schooling as in previous reports. They find genetic influences tend to be stronger for reaching BSc stage and diminish at later stages. Polygenic score prediction also improves for the transition from high-school to BSc, compared to the other contrasts.

This is a very well-written paper that addresses an important research question with rigourous theoretical and methodological approaches. I only have a few suggestions for clarifying some points, illustrated below.

Results

Figure 1 is really useful and clear. The caption for Panel A might need to add that EduYears-achievement correspondance was derived from the Norwegian school system, as it might be different in other counties. I also wonder whether the coloured areas for MSc+ and PhD in panel C were supposed to be smaller than the corresponding “Advanced” areas in panel B. If so, this is counterintuitive.

We report observed scale h2 SNP estimates for transition phenotypes to avoid assumptions about conditional population prevalences -> might be useful to clarify “We report LDSC observed scale, rather than liability-scale, h2 SNP estimates…”

“Using probit regression adjusted for sex, birth cohort, and ten ancestry principal components…” do the authors mean they controlled for birth cohort bins? If not, were different cohorts used in this analyses?

I find Figure 4 a bit misleading as it is not immediately clear they two figures represent exactly the same data, just on a different scale. To me it would be preferable (more intuitive and more transparent) to only report Figure 4b

I would be curious to see MiXer analyses complement LDSC, as we would know whether shared genetic signal is concordant or discordant in effects across transitions.

Methods

“The cohort includes extensive family relationships: 287 monozygotic twin pairs, 22,884 full siblings, 117,004 parent-offspring pairs, and thousands of second- and third-degree relative pairs.” -> how were relationships assessed, based on parent report or genetics? It would be important to clarify whether monozygotic twins were included in the GWAS analyses and how they were distinguished from duplicate samples at the genotype level.

Figure 7 I might be wrong, but I understand that “Cases” and “Controls” are named “Advanced” and “Not advanced” in Figure 1. If this is correct it would be worth using the wording consistently across all figures (as well as the colours?) Also in figure 4 they are called “Advanced” and “Did not advance”, and this term could be made consistent with the other figures too.

In the Statistical Analyses part or in the Methods, the authors should explain what the “Birth Cohort” variable represents.

I appreciate the Manhattan plots from the GWAS are in the Supplementary data, but as a reader I would want to know the lead independent significant hits at least for the GWAS with genome-wide significant results, and whether they align with EA or IQ GWAS hits.

Reviewer #4: It is uploaded as an attachment.

**********

**Have all data underlying the figures and results presented in the manuscript been provided?**

Large-scale datasets should be made available via a public repository as described in the *PLOS Genetics*  data availability policy, and numerical data that underlies graphs or summary statistics should be provided in spreadsheet form as supporting information.

Reviewer #1: Yes

Reviewer #2: Yes

Reviewer #3: Yes

Reviewer #4: Yes

**********

PLOS authors have the option to publish the peer review history of their article (what does this mean?). If published, this will include your full peer review and any attached files.

If you choose “no”, your identity will remain anonymous but your review may still be made public.

**Do you want your identity to be public for this peer review?**  For information about this choice, including consent withdrawal, please see our Privacy Policy.

Reviewer #1: No

Reviewer #2: No

Reviewer #3: No

Reviewer #4: No

[NOTE: If reviewer comments were submitted as an attachment file, they will be attached to this email and accessible via the submission site. Please log into your account, locate the manuscript record, and check for the action link "View Attachments". If this link does not appear, there are no attachment files.]

**Figure resubmission:**

While revising your submission, we strongly recommend that you use PLOS’s NAAS tool (https://ngplosjournals.pagemajik.ai/artanalysis) to test your figure files. NAAS can convert your figure files to the TIFF file type and meet basic requirements (such as print size, resolution), or provide you with a report on issues that do not meet our requirements and that NAAS cannot fix.

After uploading your figures to PLOS’s NAAS tool - https://ngplosjournals.pagemajik.ai/artanalysis, NAAS will process the files provided and display the results in the "Uploaded Files" section of the page as the processing is complete. If the uploaded figures meet our requirements (or NAAS is able to fix the files to meet our requirements), the figure will be marked as "fixed" above. If NAAS is unable to fix the files, a red "failed" label will appear above. When NAAS has confirmed that the figure files meet our requirements, please download the file via the download option, and include these NAAS processed figure files when submitting your revised manuscript.

**Reproducibility:**

To enhance the reproducibility of your results, we recommend that authors of applicable studies deposit laboratory protocols in protocols.io, where a protocol can be assigned its own identifier (DOI) such that it can be cited independently in the future. Additionally, PLOS ONE offers an option to publish peer-reviewed clinical study protocols. Read more information on sharing protocols at https://plos.org/protocols?utm_medium=editorial-email&utm_source=authorletters&utm_campaign=protocols

Attachment Submitted filename: PLOS Genetics _Beyond Years of Schooling_ review.pdf

Attachment Submitted filename: PLOS Genetics review.pdf

# Author comment: Author response to Decision Letter 1

**DOI:** 10.1371/journal.pgen.1012310.r002

23 Apr 2026

Attachment Submitted filename: Response_to_Reviewers.docx

# Peer review: Decision Letter 1

**DOI:** 10.1371/journal.pgen.1012310.r003

**Authors:** Hua Tang, Renato Polimanti

24 May 2026

PGENETICS-D-25-01264R1

Beyond years of schooling: Shifting genetic influences across educational milestones in two Norwegian cohorts

PLOS Genetics

Dear Dr. Kvalvik,

Thank you for submitting your manuscript to PLOS Genetics. After careful consideration, we feel that it has merit but does not fully meet PLOS Genetics's publication criteria as it currently stands. Therefore, we invite you to submit a revised version of the manuscript that addresses the points raised during the review process.

Please submit your revised manuscript by Jul 23 2026 11:59PM. If you will need more time than this to complete your revisions, please reply to this message or contact the journal office at plosgenetics@plos.org. When you're ready to submit your revision, log on to https://www.editorialmanager.com/pgenetics/ and select the 'Submissions Needing Revision' folder to locate your manuscript file.

Please include the following items when submitting your revised manuscript:

* A letter that responds to each point raised by the editor and reviewer(s). You should upload this letter as a separate file labeled 'Response to Reviewers'. This file does not need to include responses to any formatting updates and technical items listed in the 'Journal Requirements' section below.

* A marked-up copy of your manuscript that highlights changes made to the original version. You should upload this as a separate file labeled 'Revised Manuscript with Track Changes'.

* An unmarked version of your revised paper without tracked changes. You should upload this as a separate file labeled 'Manuscript'.

If you would like to make changes to your financial disclosure, competing interests statement, or data availability statement, please make these updates within the submission form at the time of resubmission. Guidelines for resubmitting your figure files are available below the reviewer comments at the end of this letter.

As the corresponding author, your ORCID iD is verified in the submission system and will appear in the published article. PLOS supports the use of ORCID, and we encourage all coauthors to register for an ORCID iD and use it as well. Please encourage your coauthors to verify their ORCID iD within the submission system before final acceptance, as unverified ORCID iDs will not appear in the published article. Only the individual author can complete the verification step; PLOS staff cannot verify ORCID iDs on behalf of authors.

We look forward to receiving your revised manuscript.

Kind regards,

Renato Polimanti, Ph.D.

Academic Editor

PLOS Genetics

Hua Tang

Section Editor

PLOS Genetics

Aimée Dudley

Editor-in-Chief

PLOS Genetics

Anne Goriely

Editor-in-Chief

PLOS Genetics

**Additional Editor Comments:**

While Reviewers #2 and #3 were satisfied with the authors' responses and the revised manuscript, Reviewer #1 raised important issues with the results presented and provided a simulation to support his concerns regarding potential confounders due to selection and range restriction. I would like the authors to carefully consider this information, provide a detailed response, and update their manuscript accordingly.

**Journal Requirements:**

If the reviewer comments include a recommendation to cite specific previously published works, please review and evaluate these publications to determine whether they are relevant and should be cited. There is no requirement to cite these works unless the editor has indicated otherwise.

1) We notice that your supplementary figures are uploaded with the file type 'Figure'. Please amend the file type to 'Supporting Information'. Please ensure that each Supporting Information file has a legend listed in the manuscript after the references list.

2) In the online submission form, you indicated that we are happy to provide the GWAS summary statistics generated in this study via a private Figshare link upon request. All PLOS journals now require all data underlying the findings described in their manuscript to be freely available to other researchers, either 1. In a public repository 2. Within the manuscript itself 3. Uploaded as supplementary information. This policy applies to all data except where public deposition would breach compliance with the protocol approved by your research ethics board. If your data cannot be made publicly available for ethical or legal reasons (e.g., public availability would compromise patient privacy), please explain your reasons by return email and your exemption request will be escalated to the editor for approval. Your exemption request will be handled independently and will not hold up the peer review process, but will need to be resolved should your manuscript be accepted for publication. One of the Editorial team will then be in touch if there are any issues.

3) In the online submission form, you indicated that your data will be submitted to a repository upon acceptance. We strongly recommend all authors deposit their data before acceptance, as the process can be lengthy and hold up publication timelines. Please note that, though access restrictions are acceptable now, your entire minimal dataset will need to be made freely accessible if your manuscript is accepted for publication. This policy applies to all data except where public deposition would breach compliance with the protocol approved by your research ethics board. If you are unable to adhere to our open data policy, please kindly revise your statement to explain your reasoning and we will seek the editor's input on an exemption.

**Reviewers' comments:**

Reviewer's Responses to Questions

**Comments to the Authors:**

**Please note here if the review is uploaded as an attachment.**

Reviewer #1: I've uploaded my review as an attachment.

Reviewer #2: Thank you for your thorough response. The revisions have addressed my concerns and I have no further comments.

Reviewer #3: Thank you for addressing my comments and reflecting on my suggestions.

**********

**Have all data underlying the figures and results presented in the manuscript been provided?**

Large-scale datasets should be made available via a public repository as described in the *PLOS Genetics*  data availability policy, and numerical data that underlies graphs or summary statistics should be provided in spreadsheet form as supporting information.

Reviewer #1: Yes

Reviewer #2: Yes

Reviewer #3: Yes

**********

PLOS authors have the option to publish the peer review history of their article (what does this mean?). If published, this will include your full peer review and any attached files.

If you choose “no”, your identity will remain anonymous but your review may still be made public.

**Do you want your identity to be public for this peer review?**  For information about this choice, including consent withdrawal, please see our Privacy Policy.

Reviewer #1: No

Reviewer #2: No

Reviewer #3: No

[NOTE: If reviewer comments were submitted as an attachment file, they will be attached to this email and accessible via the submission site. Please log into your account, locate the manuscript record, and check for the action link "View Attachments". If this link does not appear, there are no attachment files.]

**Figure resubmission:**

While revising your submission, we strongly recommend that you use PLOS’s NAAS tool (https://ngplosjournals.pagemajik.ai/artanalysis) to test your figure files. NAAS can convert your figure files to the TIFF file type and meet basic requirements (such as print size, resolution), or provide you with a report on issues that do not meet our requirements and that NAAS cannot fix.

After uploading your figures to PLOS’s NAAS tool - https://ngplosjournals.pagemajik.ai/artanalysis, NAAS will process the files provided and display the results in the "Uploaded Files" section of the page as the processing is complete. If the uploaded figures meet our requirements (or NAAS is able to fix the files to meet our requirements), the figure will be marked as "fixed" above. If NAAS is unable to fix the files, a red "failed" label will appear above. When NAAS has confirmed that the figure files meet our requirements, please download the file via the download option, and include these NAAS processed figure files when submitting your revised manuscript.

**Reproducibility:**

To enhance the reproducibility of your results, we recommend that authors of applicable studies deposit laboratory protocols in protocols.io, where a protocol can be assigned its own identifier (DOI) such that it can be cited independently in the future. Additionally, PLOS ONE offers an option to publish peer-reviewed clinical study protocols. Read more information on sharing protocols at https://plos.org/protocols?utm_medium=editorial-email&utm_source=authorletters&utm_campaign=protocols

Attachment Submitted filename: Review_revision_2.docx

Attachment Submitted filename: Simulation.r

# Author comment: Author response to Decision Letter 2

**DOI:** 10.1371/journal.pgen.1012310.r004

16 Jul 2026

Attachment Submitted filename: Response_to_Reviewers_auresp_2.docx

# Peer review: Decision Letter 2

**DOI:** 10.1371/journal.pgen.1012310.r005

**Authors:** Hua Tang, Renato Polimanti

11 Aug 2026

PGENETICS-D-25-01264R2

Beyond years of schooling: Genetic associations across educational milestones in two Norwegian cohorts

PLOS Genetics

Dear Dr. Kvalvik,

Thank you for submitting your manuscript to PLOS Genetics. While the manuscript has been significantly improved, few remaining comments from Reviewer #1 should be addressed. Therefore, we invite you to submit a revised version of the manuscript that addresses these points.

Please submit your revised manuscript within by Sep 10 2026 11:59PM. If you will need more time than this to complete your revisions, please reply to this message or contact the journal office at plosgenetics@plos.org. When you're ready to submit your revision, log on to https://www.editorialmanager.com/pgenetics/ and select the 'Submissions Needing Revision' folder to locate your manuscript file.

Please include the following items when submitting your revised manuscript:

* A letter that responds to each point raised by the editor and reviewer(s). You should upload this letter as a separate file labeled 'Response to Reviewers'. This file does not need to include responses to formatting updates and technical items listed in the 'Journal Requirements' section below.

* A marked-up copy of your manuscript that highlights changes made to the original version. You should upload this as a separate file labeled 'Revised Manuscript with Track Changes'.

* An unmarked version of your revised paper without tracked changes. You should upload this as a separate file labeled 'Manuscript'.

If you would like to make changes to your financial disclosure, competing interests statement, or data availability statement, please make these updates within the submission form at the time of resubmission. Guidelines for resubmitting your figure files are available below the reviewer comments at the end of this letter.

As the corresponding author, your ORCID iD is verified in the submission system and will appear in the published article. PLOS supports the use of ORCID, and we encourage all coauthors to register for an ORCID iD and use it as well. Please encourage your coauthors to verify their ORCID iD within the submission system before final acceptance, as unverified ORCID iDs will not appear in the published article. Only the individual author can complete the verification step; PLOS staff cannot verify ORCID iDs on behalf of authors.

We look forward to receiving your revised manuscript.

Kind regards,

Renato Polimanti, Ph.D.

Academic Editor

PLOS Genetics

Hua Tang

Section Editor

PLOS Genetics

Aimée Dudley

Editor-in-Chief

PLOS Genetics

Anne Goriely

Editor-in-Chief

PLOS Genetics

**Reviewers' comments:**

Reviewer's Responses to Questions

**Comments to the Authors:**

**Please note here if the review is uploaded as an attachment.**

Reviewer #1: Reviewer #1

We thank Reviewer #1 for the constructive second-round review and for providing the simulation script, which helped us identify places where the manuscript still overinterpreted attenuation across milestones.

Comment 1 — Strength of the Abstract’s final sentence

Round 1 — Reviewer #1 noted that the final sentence of the Abstract was too strong, since the heritability of cognitive ability is known to change across the lifespan. We softened the Abstract but retained a “dynamic genetic architecture” framing.

Reviewer #1): The quoted section of the abstract does not match what is currently found in the authors abstract. However, whilst the authors now appropriately acknowledge selection/range restriction, they still infer “dynamic genetic architecture” from patterns that may arise largely from conditioning on prior attainment. Their revised interpretation does soften the claim, but it still does not establish that the observed attenuation reflects distinct genetic influences rather than expected statistical consequence of selection.

Response: We agree. We have removed the “dynamic genetic architecture” framing from the Abstract and softened the interpretation throughout the manuscript. The revised Abstract now describes the findings in terms of observed genetic associations, explicitly notes the role of selection at the doctoral transition, and emphasizes the measurement implications of collapsing credentials into a single EduYears scale. It now concludes:

“At the doctoral transition, estimates were obtained among individuals who had attained at least a master’s degree, so attenuation there must be interpreted in light of selection and range restriction. Because EduYears converts distinct credentials and progression steps into a single numerical scale, estimates expressed per additional year should not be interpreted as though one year has the same meaning across the educational sequence. Milestone-preserving analyses make that hidden structure visible and sharpen the interpretation of education GWAS and PGIs.”

This revised wording no longer interprets attenuation as evidence of distinct genetic influences across stages, while retaining the main descriptive contribution of the milestone-based framework.

We also revised the Discussion to clarify that the observed composition and magnitude patterns are not uniquely interpretable within the present design:

“These composition and magnitude patterns can arise from several sources, including external phenotype coding, case composition, and sequential selection. Selection is particularly relevant to the transition-specific estimates because later contrasts are defined within progressively selected at-risk groups. However, the present design cannot quantify how much of the observed variation selection explains or exclude residual milestone-specific differences in conditional association.”

The Discussion therefore no longer treats attenuation as direct evidence that distinct genetic effects operate at different educational stages. Instead, it presents external measurement, case composition, sequential selection, statistical precision, and residual transition-specific differences in conditional association as potentially contributing explanations. We also distinguish the repeated conditioning inherent in the transition-specific framework from cumulative attained-status analyses conducted in the full cohort, which are less directly exposed to this specific mechanism but remain sensitive to endpoint prevalence and case imbalance.

Reviewer 1# response

I’m happy that the authors have toned down the language used. I’m happy with the abstract and the discussion change. I would say that the analyses do not only fail to separate selection from milestone-specific genetic effects but also that they do not separate simple selection from more realistic forms of selection. The current simulation represents one illustrative constant effect model. It does not establish that more complex constant effect models would necessarily fail to reproduce the observed attenuation.

Consequently, the manuscript still leaves the impression that there may be a residual milestone-specific component once selection has been considered, whereas the evidence does not distinguish this possibility from other selection mechanisms.

Comment 2 — Selection and range restriction versus “dynamic architecture”; the simulation

Round 1 — Reviewer #1 cautioned that a biological reading of the attenuation (e.g., a “two-component architecture”) overinterprets a pattern that is expected under range restriction in increasingly selected groups. We reframed the results around selection, deleted the “two-component architecture” sentence, and added supplementary PGI analyses.

Reviewer #1: The finding that the PGI rises for both advancers and non-advancers is, as the authors state, very likely due to the increasingly selected groups. It is consistent with my initial concern that, rather than a dynamic genetic architecture across education milestones, there is an increasingly smaller range of ability at the higher levels of educational attainment. This supplementary analysis reinforces a statistical explanation of the authors results more than the “dynamic architecture” interpretation.

To illustrate my point, I’ve created a script where every genetic predictor has the same statistical effect at every educational transition. Thus, any weakening seen later therefore cannot be because the PGI “matters less”; it must come from selection/range restriction. The simulation shows that attenuation in transition-specific PGI prediction or genetic correlations can arise simply from conditioning on prior educational success. Because the same genetic effect is imposed at every transition, the apparent weakening at later transitions cannot be interpreted as evidence that genetic influences are less important at higher educational levels. It is a consequence of sequential selection and range restriction.

From my simulations restriction alone produces highly similar effects to those found by the authors. I have included these simulations at the end of this review.

Response: We agree with the reviewer’s central interpretive point, and we thank Reviewer #1 for providing the simulation script. We adapted and expanded the script into Supplementary Note 4, with full annotated R code and two new figures (Supplementary Figs. S44–S45). The simulation holds the coefficient of the simulated PGI constant across all four transitions. Despite this, sequential thresholding raises the mean PGI and reduces PGI variance in later at-risk groups. Together with endpoint case imbalance, this produces attenuation in transition-specific PGI discrimination.

We have also revised the manuscript to distinguish carefully between what the simulation demonstrates directly and the broader interpretive implications. The simulation directly evaluates PGI discrimination under a constant simulated coefficient. It does not directly model genetic correlations or SNP heritability. The revised Discussion now states:

“Supplementary Note 4 illustrates this point directly for transition-specific PGI discrimination. With the simulated PGI coefficient held constant across transitions, sequential thresholding raises mean PGI and reduces PGI variance in later at-risk groups. Together with endpoint case imbalance, this lowers Tjur’s R2, while the per-SD odds ratio remains approximately stable (Supplementary Figs. S44-S45). The simulation is an illustrative scenario rather than a model fitted to the MoBa data, and it does not directly model genetic correlations or h2SNP. It shows that declining transition-specific PGI discrimination is compatible with a constant PGI coefficient and therefore cannot, by itself, be attributed to weaker conditional associations at later milestones. Related conditioning and case-composition concerns also bear on the interpretation of transition-specific genetic correlations and observed-scale h2SNP, although those quantities are not directly evaluated by the simulation.”

We further clarified that mean PGI, Tjur’s R2, and per-SD odds ratios describe different aspects of the selected at-risk groups and should not be interpreted interchangeably. The revised Discussion states:

“The changing composition of these at-risk sets is visible in the supplementary PGI analyses. For the intelligence-related, CogSub, and NonCogSub PGIs, mean scores increased across transitions among both advancers and non-advancers, indicating that later comparisons are drawn from groups increasingly enriched for education-related, cognitive, and non-cognitive genetic propensities (Supplementary Fig. S39). Mean PGI, Tjur’s R2, and the per-SD odds ratio capture different features of these selected groups. Mean PGI describes the composition of the at-risk set. Tjur’s R2 quantifies observed-scale discrimination between advancers and non-advancers within that set. The odds ratio estimates the conditional association of a one-SD higher PGI with advancement. These quantities should therefore not be interpreted interchangeably.”

The empirical odds ratios for the intelligence-related and GWAS-by-subtraction PGIs tended to decline across later transitions, whereas the odds ratio remained approximately stable in the constant-coefficient simulation. We therefore do not attribute the empirical odds-ratio attenuation to range restriction alone.

The manuscript now interprets these trajectories as compatible with selection-induced dependence, residual transition-specific differences in conditional association, or both, while stating that the present design cannot separate these possibilities.

Finally, we removed language implying that weaker observed signal means that genetic factors matter less at later educational stages. The revised manuscript instead states that later transition-specific attenuation is compatible with sequential selection and case composition, while the relative contributions of selection and residual transition-specific differences remain unresolved. The milestone framework is therefore presented as revealing how observed genetic associations and PGI discrimination depend on the educational contrast and the population being compared, rather than as demonstrating a dynamic genetic architecture.

Reviewer 1 comment

The authors have substantially addressed my principal concern. They now acknowledge that attenuation of PGI discrimination can arise entirely through sequential selection and range restriction and explicitly state that their design cannot distinguish selection-induced attenuation from genuine transition-specific differences in genetic association.

As such, I no longer view the manuscript as overinterpreting its findings. However, readers should understand that the analyses remain fundamentally descriptive. That is, they demonstrate changing observed genetic associations across educational transitions, but do not identify whether these differences reflect selection, altered variance, or changes in underlying genetic effects (i.e. a dynamic genetic architecture).

However, I would like to highlight an asymmetry in the discussion on line 489 reading “The empirical odds-ratio trajectories are therefore compatible with selection-induced dependence and residual milestone-specific differences in conditional association, which the present design cannot separate”. This is a scientifically safe statement; however, they have demonstrated that selection alone can reproduce the qualitative patterns in their PGRS results. They have not demonstrated that any residual milestone-specific effects actually exist. I fully accept there may very well be milestone specific genetic effects however, this manuscript does not provide evidence for their existence, nor are they required to explain the observed patterns of association.

The sole piece of evidence the authors use to push back against the simulations explaining their observations is that the odds ratios are not consistent in their real data, but are consistent in the simulations. The empirical attenuation of the odds ratios is introduced in the Discussion as the principal reason why the illustrative simulation does not fully explain the observed data. However, these odds-ratio results are neither presented nor formally evaluated in the Results section. If the authors wish to rely on this observation as evidence that their empirical data differ from the illustrative simulation, then it should first be demonstrated formally (e.g. by testing whether OR(Comp→HS+) differs significantly from OR(MSc→PhD)).

If this difference is significant, I do not think it good evidence for the existence of milestone-specific genetic effects, but only evidence that the very simple selection modelled does not capture nuance such as inducing dependence between the PGRS and other factors (e.g. motivation), assuming one latent liability, one constant regression coefficient, sequential selection, and independent residual error at each transition.

Importantly, I don’t think any of this is a problem. This is a well conducted piece of science, testing a well thought through, and interesting, hypotheses based on the observation that the genetic architecture of education appears to change across education mile-stones. My issue is the discussion. The authors make the argument that they cannot distinguish between the consequences of selection and mile-stone specific genetic effects, but the simulations have shown that selection reproduces the pattern of the PGRS results.

The above leads me to believe there is a potentially much more interesting way to frame these results. Although the revised Discussion appropriately emphasises selection, it continues to give considerable weight to the possibility of milestone-specific genetic effects, but this seems forced in light of their empirical observations. Specifically, the authors have shown that educational transitions appear to show progressively weaker genetic prediction. The authors then, quite rightly, postulate that this pattern may be interpreted as a changing genetic architecture. However, we now see that a very simple sequential selection model produces, almost, the same observed results. The discussion could conclude something along the lines of “The present analyses provide no direct evidence that transition-specific attenuation reflects milestone-specific genetic effects. Instead, the observed patterns are compatible with the sequential selection induced by educational progression. Although residual milestone-specific differences cannot be excluded, they are not required to explain the empirical observations.”. I think this much more fair.

Comment 4 — Choice and entanglement of the intelligence reference

Round 1 — Reviewer #1 questioned the “IQ3” label and our use of Savage et al. (2018) rather than the larger Lam et al. (2021). We relabelled the trait, retained Savage et al. as the primary reference, and added Lam et al. (2021) as a sensitivity analysis.

Reviewer #1): (a) Great. (b) & (c) The MTAG data set to which the authors refer has already been used to disentangle genetic influences on education from those of intelligence. The Lam et al data set consists of Savage + Davies. As such the effect of conditioning on ES is still present.

Response: We thank the reviewer for this clarification and agree that the Lam et al. sensitivity analysis neither eliminates the strong coupling between education and intelligence nor provides a cleanly independent intelligence comparator. We therefore no longer present it as separating education-related from intelligence-related genetic signal; it now appears only as a sensitivity reference showing that the broad milestone trajectory is similar under an alternative intelligence GWAS. The revised Discussion states:

“EA4 and intelligence should also be treated as overlapping rather than orthogonal reference traits, given their strong phenotypic and genetic coupling [36,37].”

We further clarify that the GWAS-by-subtraction cognitive and non-cognitive components were constructed jointly from the EA3 educational-attainment and cognitive-performance GWASs, both reported by Lee et al. (2018), and therefore do not provide a comparison fully independent of the EduYears GWAS lineage underlying EA4. The cited references, Davies et al. (2019) and Anderson et al. (2020), have been added to the reference list.

Reviewer 1 comment

Some thing has been lost here. I don’t think the authors have responded to my comment. First, (or a as it is recorded in prior responses) the authors have now correctly labelled IQ3 as Savage et al. I think this is good as Savage is not the third IQ GWAS study. It uses the UKBiobank data first analysed by Hill et al. and subsequently by Davies et al and so replicates most of these known loci.

Second (b), the authors state they used Savage as it does not include education via MTAG. I argued that the Hill et al data (which did use MTAG and an education data set) has subsequently been used to disentangle the effects of education and intelligence (in the Davies et al. (2019) and Anderson et al. (2020) papers) so the author reluctance to use this data is unwarranted (but understandable).

Third (c), Lam is a combination of Davies and Savage, as such the fluid intelligence data is conditioned phenotypically on the Townsend score (a measure of SES). Conditioning on heritable traits prior to GWAS can result in some odd findings. If the findings are attributable to conditioning on townsend in the Savage data then using the Lam data may very well result in the same confound producing the same results.

**********

**Have all data underlying the figures and results presented in the manuscript been provided?**

Large-scale datasets should be made available via a public repository as described in the *PLOS Genetics*  data availability policy, and numerical data that underlies graphs or summary statistics should be provided in spreadsheet form as supporting information.

Reviewer #1: Yes

**********

PLOS authors have the option to publish the peer review history of their article (what does this mean?). If published, this will include your full peer review and any attached files.

If you choose “no”, your identity will remain anonymous but your review may still be made public.

**Do you want your identity to be public for this peer review?**  For information about this choice, including consent withdrawal, please see our Privacy Policy.

Reviewer #1: No

[NOTE: If reviewer comments were submitted as an attachment file, they will be attached to this email and accessible via the submission site. Please log into your account, locate the manuscript record, and check for the action link "View Attachments". If this link does not appear, there are no attachment files.]

**Figure resubmission:**

While revising your submission, we strongly recommend that you use PLOS’s NAAS tool (https://ngplosjournals.pagemajik.ai/artanalysis) to test your figure files. NAAS can convert your figure files to the TIFF file type and meet basic requirements (such as print size, resolution), or provide you with a report on issues that do not meet our requirements and that NAAS cannot fix.

After uploading your figures to PLOS’s NAAS tool - https://ngplosjournals.pagemajik.ai/artanalysis, NAAS will process the files provided and display the results in the "Uploaded Files" section of the page as the processing is complete. If the uploaded figures meet our requirements (or NAAS is able to fix the files to meet our requirements), the figure will be marked as "fixed" above. If NAAS is unable to fix the files, a red "failed" label will appear above. When NAAS has confirmed that the figure files meet our requirements, please download the file via the download option, and include these NAAS processed figure files when submitting your revised manuscript.

**Reproducibility:**

To enhance the reproducibility of your results, we recommend that authors deposit laboratory protocols in protocols.io, where a protocol can be assigned its own identifier (DOI) such that it can be cited independently in the future. Additionally, PLOS ONE offers an option to publish peer-reviewed clinical study protocols. Read more information on sharing protocols at https://plos.org/protocols?utm_medium=editorial-email&utm_source=authorletters&utm_campaign=protocols

# Author comment: Author response to Decision Letter 3

**DOI:** 10.1371/journal.pgen.1012310.r006

24 Aug 2026

Attachment Submitted filename: Response_to_Reviewers_auresp_3.docx

# Editor report: Decision Letter 3

**DOI:** 10.1371/journal.pgen.1012310.r007

**Authors:** Hua Tang, Renato Polimanti

31 Aug 2026

Dear Dr Kvalvik,

We are pleased to inform you that your manuscript entitled "Beyond years of schooling: Genetic associations across educational milestones in two Norwegian cohorts" has been editorially accepted for publication in PLOS Genetics. Congratulations!

Before your submission can be formally accepted and sent to production you will need to complete our formatting changes, which you will receive in a follow up email. Please be aware that it may take several days for you to receive this email; during this time no action is required by you. Please note: the accept date on your published article will reflect the date of this provisional acceptance, but your manuscript will not be scheduled for publication until the required changes have been made.

Once your paper is formally accepted, an uncorrected proof of your manuscript will be published online ahead of the final version, unless you’ve already opted out via the online submission form. If, for any reason, you do not want an earlier version of your manuscript published online or are unsure if you have already indicated as such, please let the journal staff know immediately at plosgenetics@plos.org.

In the meantime, please log into Editorial Manager at https://www.editorialmanager.com/pgenetics/, click the "Update My Information" link at the top of the page, and update your user information to ensure an efficient production and billing process. Note that PLOS requires an ORCID iD for all corresponding authors. Therefore, please ensure that you have an ORCID iD and that it is validated in Editorial Manager. To do this, go to ‘Update my Information’ (in the upper left-hand corner of the main menu), and click on the Fetch/Validate link next to the ORCID field.  This will take you to the ORCID site and allow you to create a new iD or authenticate a pre-existing iD in Editorial Manager.

If you have a press-related query, or would like to know about making your underlying data available (as you will be aware, this is required for publication), please see the end of this email. If your institution or institutions have a press office, please notify them about your upcoming article at this point, to enable them to help maximise its impact. Inform journal staff as soon as possible if you are preparing a press release for your article and need a publication date.

Thank you again for supporting open-access publishing; we are looking forward to publishing your work in PLOS Genetics!

Yours sincerely,

Renato Polimanti, Ph.D.

Academic Editor

PLOS Genetics

Hua Tang

Section Editor

PLOS Genetics

Aimée Dudley

Editor-in-Chief

PLOS Genetics

Anne Goriely

Editor-in-Chief

PLOS Genetics

www.plosgenetics.org

BlueSky: @plos.bsky.social

----------------------------------------------------

Comments from the reviewers (if applicable):

----------------------------------------------------

**Data Deposition**

If you have submitted a Research Article or Front Matter that has associated data that are not suitable for deposition in a subject-specific public repository (such as GenBank or ArrayExpress), one way to make that data available is to deposit it in the Dryad Digital Repository. As you may recall, we ask all authors to agree to make data available; this is one way to achieve that. A full list of recommended repositories can be found on our website.

The following link will take you to the Dryad record for your article, so you won't have to re‐enter its bibliographic information, and can upload your files directly:

http://datadryad.org/submit?journalID=pgenetics&manu=PGENETICS-D-25-01264R3

More information about depositing data in Dryad is available at http://www.datadryad.org/depositing. If you experience any difficulties in submitting your data, please contact help@datadryad.org for support.

Additionally, please be aware that our data availability policy requires that all numerical data underlying display items are included with the submission, and you will need to provide this before we can formally accept your manuscript, if not already present.

----------------------------------------------------

**Press Queries**

If you or your institution will be preparing press materials for this manuscript, or if you need to know your paper's publication date for media purposes, please inform the journal staff as soon as possible so that your submission can be scheduled accordingly. Your manuscript will remain under a strict press embargo until the publication date and time. This means an early version of your manuscript will not be published ahead of your final version. PLOS Genetics may also choose to issue a press release for your article. If there's anything the journal should know or you'd like more information, please get in touch via plosgenetics@plos.org.

# Editor report: Acceptance letter

**DOI:** 10.1371/journal.pgen.1012310.r008

**Authors:** Hua Tang, Renato Polimanti

PGENETICS-D-25-01264R3

Beyond years of schooling: Genetic associations across educational milestones in two Norwegian cohorts

Dear Dr Kvalvik,

We are pleased to inform you that your manuscript entitled "Beyond years of schooling: Genetic associations across educational milestones in two Norwegian cohorts" has been formally accepted for publication in PLOS Genetics! Your manuscript is now with our production department and you will be notified of the publication date in due course.

The corresponding author will soon be receiving a typeset proof for review, to ensure errors have not been introduced during production. Please review the PDF proof of your manuscript carefully, as this is the last chance to correct any errors. Please note that major changes, or those which affect the scientific understanding of the work, will likely cause delays to the publication date of your manuscript.

Soon after your final files are uploaded, unless you have opted out or your manuscript is a front-matter piece, the early version of your manuscript will be published online. The date of the early version will be your article's publication date. The final article will be published to the same URL, and all versions of the paper will be accessible to readers.

For Research Articles, you will receive an invoice from PLOS for your publication fee after your manuscript has reached the completed accept phase. If you receive an email requesting payment before acceptance or for any other service, this may be a phishing scheme. Learn how to identify phishing emails and protect your accounts at https://explore.plos.org/phishing.

Thank you again for supporting PLOS Genetics and open-access publishing. We are looking forward to publishing your work!

With kind regards,

Sharmila Kamatchi

PLOS Genetics

On behalf of:

The PLOS Genetics Team

Carlyle House, Carlyle Road, Cambridge CB4 3DN | United Kingdom

plosgenetics@plos.org | +44 (0) 1223-442823

plosgenetics.org | Twitter: @PLOSGenetics
